# Principled multi-omic analysis reveals gene regulatory mechanisms of phenotype variation

**DOI:** 10.1101/gr.227066.117

**Published:** 2018-08

**Authors:** Casey Hanson, Junmei Cairns, Liewei Wang, Saurabh Sinha

**Affiliations:** 1 Department of Computer Science, University of Illinois at Urbana-Champaign, Urbana, Illinois 61801, USA;; 2 Division of Clinical Pharmacology, Department of Molecular Pharmacology and Experimental Therapeutics, Mayo Clinic, Rochester, Minnesota 55905, USA;; 3 Department of Computer Science and Institute of Genomic Biology, University of Illinois at Urbana-Champaign, Urbana, Illinois 61801, USA

## Abstract

Recent studies have analyzed large-scale data sets of gene expression to identify genes associated with interindividual variation in phenotypes ranging from cancer subtypes to drug sensitivity, promising new avenues of research in personalized medicine. However, gene expression data alone is limited in its ability to reveal *cis*-regulatory mechanisms underlying phenotypic differences. In this study, we develop a new probabilistic model, called pGENMi, that integrates multi-omic data to investigate the transcriptional regulatory mechanisms underlying interindividual variation of a specific phenotype—that of cell line response to cytotoxic treatment. In particular, pGENMi simultaneously analyzes genotype, DNA methylation, gene expression, and transcription factor (TF)-DNA binding data, along with phenotypic measurements, to identify TFs regulating the phenotype. It does so by combining statistical information about expression quantitative trait loci (eQTLs) and expression-correlated methylation marks (eQTMs) located within TF binding sites, as well as observed correlations between gene expression and phenotype variation. Application of pGENMi to data from a panel of lymphoblastoid cell lines treated with 24 drugs, in conjunction with ENCODE TF ChIP data, yielded a number of known as well as novel (TF, Drug) associations. Experimental validations by TF knockdown confirmed 41% of the predicted and tested associations, compared to a 12% confirmation rate of tested nonassociations (controls). An extensive literature survey also corroborated 62% of the predicted associations above a stringent threshold. Moreover, associations predicted only when combining eQTL and eQTM data showed higher precision compared to an eQTL-only or eQTM-only analysis using pGENMi, further demonstrating the value of multi-omic integrative analysis.

There is great interest today in understanding why certain drugs are effective in some individuals but less so in others. Many studies have sought to identify mechanisms of action of specific drugs (Iorio et al. 2010; Gregori-Puigjane et al. 2012; Schenone et al. 2013) as well as genotypic variations that are predictive of an individual's drug response (Madian et al. 2012; Moen et al. 2012; Nelson et al. 2016). A major class of drugs of interest today are cytotoxic drugs that may be used in cancer treatment. Large-scale data generation efforts, including genotypic and molecular profiling of panels of cell lines (Barretina et al. 2012; Forbes et al. 2015) along with drug response (cytotoxicity) measurement on those cell lines (Barretina et al. 2012; Yang et al. 2013; Rees et al. 2016), are expected to facilitate future advances in cancer pharmacogenomics. As the diversity of such data sets increases, it is important to devise rigorous computational methods that can combine these diverse data in a principled manner to help scientists answer mechanistic as well as therapeutic questions pertaining to drug response. For instance, correlating gene expression and drug response in a panel of cell lines helps identify cytotoxicity-related genes (Rees et al. 2016), but it is not clear how one might extend the approach to additionally exploit genotype (SNP) and epigenotype data (e.g., CpG methylation marks) to maximum effect. We need a statistically sound approach capable of modeling phenotypic variation while integrating several heterogeneous genomic and epigenomic data types.

One of the key mechanistic questions related to drug response variation, or indeed for any phenotypic variation under study, is the role of gene regulatory networks (GRNs) in shaping such variation. A major step in characterizing GRNs mediating drug response variation is to identify functionally important transcription factors (TFs), as TFs are the main actors in any GRN (Gariboldi et al. 2007; Gomes et al. 2013). Identified TFs may then be experimentally validated by showing that their knockdown leads to a change in chemosensitivity (Hanson et al. 2015; Long et al. 2015; Faiao-Flores et al. 2017). Mechanistically speaking, a TF may influence drug response by regulating one or more target genes whose expression levels are in turn linked to the strength of cytotoxic response, e.g., if the target genes are involved in apoptotic pathways. In such a case, one expects evidence of the TF's regulatory influence on the target gene(s) in the form of binding sites revealed as ChIP peaks (Hanson et al. 2015). Thus, if one finds substantial evidence that TF binding sites harbor genotype or epigenotype variations that are, in turn, correlated with gene expression and the phenotype, it should be possible to statistically implicate that TF in drug response variation. This is the key insight pursued in this work, to identify major transcriptional regulators of drug response variation across individuals.

There have been a number of studies linking specific phenotypes such as disease status to elements of the noncoding genome (Schaub et al. 2012; Ward and Kellis 2012; Corradin and Scacheri 2014), many utilizing the NHGRI Genome-Wide Association (GWA) Catalog SNPs (Welter et al. 2014). These studies have been facilitated by large-scale efforts such as the ENCODE Project (The ENCODE Project Consortium 2012) and the Epigenomics Roadmap Project (Bernstein et al. 2010) that provide a guide to identifying noncoding elements of the genome, which can then be used as a regulatory context to interpret GWA SNPs. Additionally, there have been studies that quantify the impact of noncoding genetic variation on molecular profiles such as TF-DNA binding or DNA accessibility (Lee et al. 2015; Zhou and Troyanskaya 2015), often utilizing the NHGRI GWA catalog to assess the phenotypic consequences of variants. Despite numerous efforts to connect phenotype with genotype and regulatory elements, there has not been a systematic effort to aggregate such connections to learn major regulatory mechanisms underlying the genotype-phenotype relationship and its variation across individuals. Furthermore, the regulatory impact of epigenetic sources of variation (for instance, CpG methylation) are usually assessed in isolation from genetic variants (SNPs). Recent studies have shown that there may be a complex interplay between genetic, epigenetic, and transcriptional variation in relation to disease (Jones et al. 2013), arguing for a more integrative approach to their analysis.

We present here a novel, statistically principled approach to aggregating data on genetic as well as epigenetic variations, along with genome-wide profiles of regulatory function, to derive associations between a transcription factor and individual variation in cytotoxic response to a drug; this permits a mechanistic interpretation of the impact of molecular variants on drug response. There have been new insights into how TFs may be regulated by small molecules (Fontaine et al. 2015; Papavassiliou and Papavassiliou 2016), and there is no doubt as to their significance in human diseases such as cancer (Redell and Tweardy 2006; Redmond and Carroll 2009; Yeh et al. 2013). Our aim therefore is to push the frontier of knowledge regarding the relationship of TFs and drug response for putative therapeutic benefit. Our new computational method is based on a statistical formalism called probabilistic graphical models (Koller and Friedman 2009), which are among the most flexible and principled ways available today for inference from heterogeneous and noisy data. Using a rigorous data preprocessing pipeline in conjunction with this powerful framework, we demonstrate that the (TF, Drug) associations predicted by the method are accurate by showing that knockdown of the TF affects sensitivity to the drug. The new method is also applicable to other studies where one seeks mechanistic factors underlying individual variation in a quantitative phenotype in the presence of genotype and epigenotype information.

## Results

### A new probabilistic model to integrate genotype, gene expression, and phenotype data

In our previous work, we presented a proof-of-principle method that identifies TFs associated with individual variation in drug response or any other quantitative phenotype (Hanson et al. 2015). We searched for cases where a SNP in the *cis*-regulatory region of a gene is correlated with the gene's expression (*cis*-eQTL analysis) and the gene's expression is correlated with phenotype—the latter being referred to as a transcriptome-wide association study (TWAS) (Cloney 2016); if significantly many cases like this were identified involving SNPs within ChIP peaks of a TF, then the TF was considered associated with individual variation in phenotype. Here, we constructed a rigorous probabilistic model that builds upon this idea to identify phenotype-related TFs. The new method is called “pGENMi” (the previous tool was named “GENMi” for “gene expression in the middle” and the “p” denotes a probabilistic model). It integrates information from many genes whose expression correlates with the phenotype and for which it can find evidence supporting regulatory influence of a specific TF. The probabilistic formulation of pGENMi offers the following important features:

1. It integrates gene expression-phenotype association without relying on strict thresholds.
2. As evidence for a TF's role in gene expression variation, it can utilize information about different types of expression-linked *cis*-variants (genetic as well as epigenetic) located in the TF's binding sites near the gene.
3. In incorporating multiple sources of evidence for a TF's regulatory role, it can weight the contribution of each type of evidence differently, learning these relative weights automatically.

**Figure 1.**
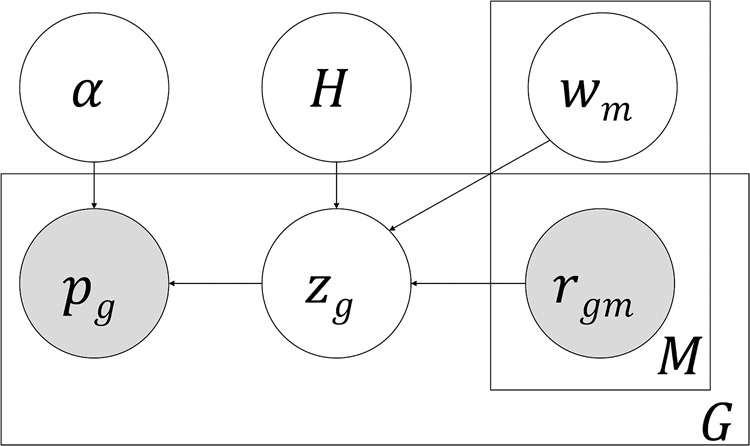
Plate diagram of pGENMi model. A latent variable *z*_*g*_ represents whether a gene *g* mediates the influence of a TF on phenotype, and its enclosing rectangle denotes *G* such genes. *p*_*g*_ denotes the TWAS *P*-value between the gene's expression and phenotype variation for each of the *G* genes. If *z*_*g*_ = 1, we expect an enrichment for significant TWAS *P*-values, and *p*_*g*_ is modeled by a Beta distribution parameterized by *α*; otherwise, *p*_*g*_ is modeled as being distributed uniformly in [0,1]. We also observe one or more lines of evidence supporting the TF's influence on the expression of each gene *g*, such as the existence of a *cis*-eQTL within a ChIP peak of the TF near that gene. These “regulatory evidences” are denoted by the binary variables *r*_*gm*_, and there exist *M* such types of evidence (*m* = 1 … *M*), with relative weights *w*_*m*_. These evidences combine in a logistic function to determine Pr(*z*_*g*_ = 1). The weights *w*_*m*_ are learned over all genes, and as such, are shown *outside* of the rectangle enclosing *G*. The *H* variable indicates whether *w*_*m*_ is free or restricted to zero (null model) for hypothesis testing (see “Probabilistic graphical model” section in Methods).

The probabilistic model of pGENMi is described in Figure 1 and in Methods, but we present the main ideas here. The model is evaluated separately for each TF and provides a log-likelihood ratio score to quantify the TFs’ role in regulating phenotypic variation, considering all available data. It assigns to each gene *g* a “hidden” variable *z*_*g*_ that takes a value of 1 if the gene *g* is a mediator of the TF's influence on the phenotype, and 0 otherwise. The case of *z*_*g*_ = 1 is supported if the gene's expression is correlated with the phenotype (TWAS *P*-value close to 0), and one or more lines of evidence support the TF's role in regulating that gene. Such “regulatory evidence” may include the existence of a significant *cis*-eQTL within the TF's ChIP peak located near the gene. Specifically, the probability of *z*_*g*_ = 1 is determined by a linear combination of one or more regulatory evidences, using a single free parameter as the relative weight of each type of evidence. Intuitively, a TF is considered as a potential regulator of the phenotype if the data supports the existence of several genes mediating its influence on the phenotype, as reflected in their *z*_*g*_ variables.

### pGENMi integrates multiple types of regulatory evidence to elucidate the role of TFs in drug response variation

We applied the pGENMi algorithm to identify TFs that putatively regulate individual variation in drug response. We considered drug response data of 24 cytotoxic drugs (or treatments) assayed on 284 lymphoblastoid cell lines (LCLs). This phenotypic data was analyzed in conjunction with genotype (SNP), CpG methylation, and gene expression data (see Methods) on the same panel of 284 LCLs, along with ENCODE ChIP-seq data on TF-DNA binding. To assess the regulatory evidence for a TF's role on a gene's expression variation, we first identified the strongest *cis*-eQTL SNP located near the gene (50 kb upstream of the transcription start site) and within the TF's ChIP peaks; an eQTL *P*-value ≤ 0.05 was considered as evidence for the TF's role in regulating the gene. We similarly tallied “eQTM” evidence, i.e., a methylation mark significantly correlated with gene expression (see Methods), located near the gene (*cis*-eQTM) and within the TF's ChIP peaks. The eQTL and eQTM evidences for the TF's regulatory influence on genes were then integrated with gene expression-phenotype associations (TWAS) using the pGENMi model. The result of this analysis is a ranking (by LLR score) of all TFs by their predicted role in regulating each drug response phenotype. We refer to this mode of analysis as “eQTL + M” analysis; we also repeated the entire procedure using eQTL-only evidence and eQTM-only evidence.

In computing the various correlations (TWAS, eQTL, and eQTM) used in pGENMi analyses, we included as covariates all information for which we had data per individual, i.e., sex, age, and batch, as well as subpopulation axes of variation inferred from ethnic labels and genotype information using EIGENSTRAT (Price et al. 2006); see Supplemental Note S3. Also, we relied on clusters of TF ChIP peaks from many different cell lines, as computed by the ENCODE project, rather than peaks from LCLs exclusively. We believed this would make the regulatory inferences more generalizable across cell lines and the ChIP peaks themselves more likely to be functional. (See Supplemental Note S3; Supplemental Tables S4–S7; and Supplemental Fig. S14 for a comparison to the alternative strategy of using TF peaks exclusively from LCLs).

### Literature evidence in support of pGENMi predicted (TF, Drug) associations

In the absence of a gold standard benchmark, we performed an extensive literature search for experimental results corroborating pGENMi-predicted (TF, Drug) associations, using two criteria. The first, and most convincing, is “direct” experimental evidence demonstrating that knockdown of the TF affects chemosensitivity to the corresponding drug. The second is “indirect” experimental evidence demonstrating differential expression or DNA binding of the TF induced by the drug.

Initially, we restricted our literature search to eQTL + M associations with LLR ≥ 4.5, roughly corresponding to a *P*-value of 0.05 (χ^2^ test). Of the 19 reported associations at this stringent criterion, 14 were validated by the literature (eight directly and six indirectly), as shown in Table 1 (see detailed survey in Supplemental Note S1). For example, pGENMi reported FOXM1 as a top scoring association for the drug temozolomide; this prediction was validated by a study where siRNA knockdown of *FOXM1* was shown to sensitize recurrent glioblastoma multiforme (GBM) tumors to this drug (Zhang et al. 2012). pGENMi also predicted STAT3 as being associated with response to oxaliplatin. Indeed, siRNA silencing of *STAT3* combined with oxaliplatin therapy, in mouse models of metastatic colorectal cancer (HCT116), was previously found to reduce tumor size better than either drug separately (Shahzad et al. 2011). An example of indirect validation is that of the predicted association between GATA1 and rapamycin. Treatment of hexamethylene bisacetamide (HMBA), which commits cells to cessation of growth and differentiation, to Friend erythroleukemia cells increased DNA binding of GATA1, an important TF for erythroid-specific genes. When treated with the S6-kinase inhibitor, rapamycin, HMBA cells induced at 18 h showed markedly lower binding of GATA1 to the DNA (Bavelloni et al. 2000).

**Table 1.**
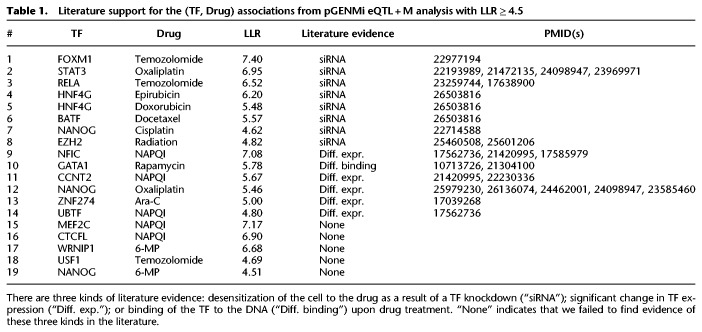
Literature support for the (TF, Drug) associations from pGENMi eQTL + M analysis with LLR ≥ 4.5

**Figure 2.**
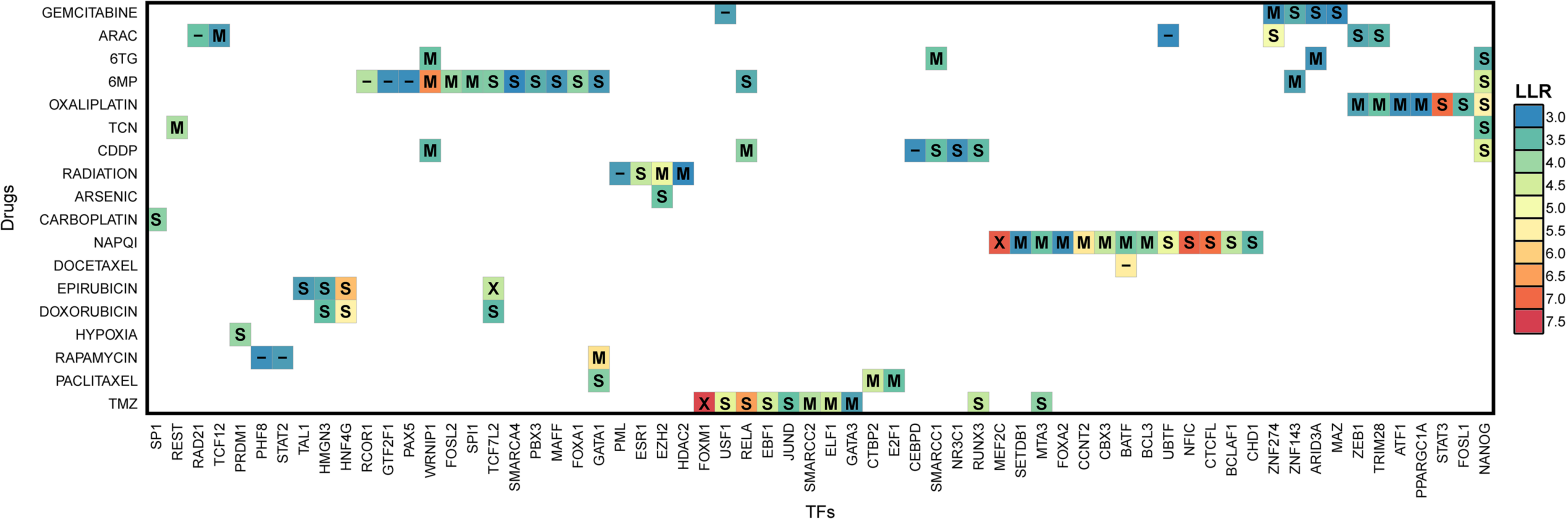
The 90 (TF, Drug) associations predicted by eQTL + M with LLR ≥ 3, colored by LLR. Labels indicate whether eQTL-only or eQTM-only analysis corroborated the prediction at LLR ≥ 1.74 (labels “S” and “M,” respectively). An “X” indicates both eQTL and eQTM analysis supported the prediction, while “–” indicates the prediction is unique to eQTL + M.

Some drug responses may be easier to model using gene expression than others, due to variation in the quality of experiments and different mechanisms of action. As such, we sought to examine how literature-based validations of pGENMi associations segregate by drug. To do this, we relaxed the LLR threshold to 3, which resulted in 90 (TF, Drug) associations (Fig. 2). We further restricted our literature survey to, at most, the top seven associations for each drug (to limit the burden of manual validation), amounting to 73 associations. The precision (fraction of positive predictions supported by literature evidence) of these top associations varies widely across drugs (Table 2). For certain drugs, such as cisplatin, all seven predictions were confirmed by direct or indirect literature evidence. We also observed 100% precision for the following drugs for which a single TF was predicted: arsenic, carboplatin, docetaxel, and hypoxia. On the other hand, TFs associated with certain drugs, e.g., 6-MP and 6-TG, were rarely supported by the literature. Overall, our literature survey (detailed in Supplemental Note S2) resulted in validation of 45 of 73 (62%) predictions made at the modest threshold of LLR ≥ 3 but pointed to substantial interdrug variability in pGENMi's ability to identify TF determinants of cytotoxicity.

**Table 2.**
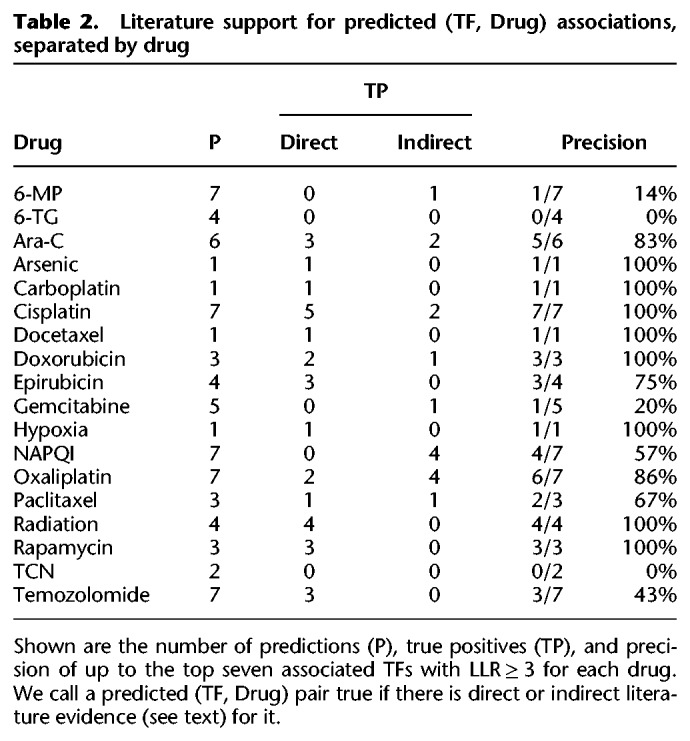
Literature support for predicted (TF, Drug) associations, separated by drug

We also assessed the benefit of modeling multiple types of regulatory evidence simultaneously (i.e., eQTL + M) rather than simply taking the union of associations reported by running pGENMi for each evidence (eQTL or eQTM) separately. To answer this question, we ran eQTL-only and eQTM-only analyses and applied an LLR threshold of 1.74 (roughly corresponding to χ^2^ *P*-value of 0.05) for their reported associations. We then categorized the 90 eQTL + M associations with LLR ≥ 3 (Fig. 2) based on their recapitulation in eQTL- and eQTM-only analysis (Table 3). The eQTL + M associations were rarely supported by both analyses. Despite this, 11 associations were identified by the eQTL + M model that a simple intersection would not produce; we further investigated these associations in our experimental validation. In looking at the top 500 (TF, drug) associations reported by each analysis, we noted that the eQTL + M analysis and eQTL-only analysis showed strong concordance (309 common associations), while the eQTM-only analysis showed much less concordance with results from the other two analyses (Supplemental Tables S1–S3).

**Table 3.**
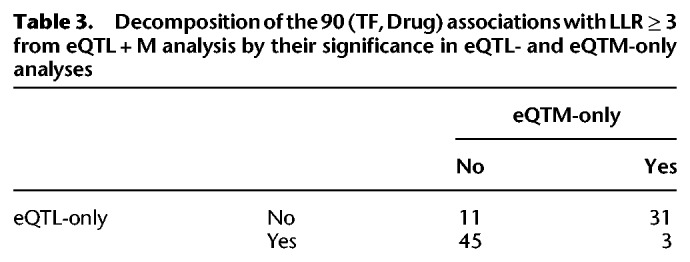
Decomposition of the 90 (TF, Drug) associations with LLR ≥ 3 from eQTL + M analysis by their significance in eQTL- and eQTM-only analyses

### Experimental validation

We sought to experimentally verify whether TFs associated with drug response variation (by pGENMi) could be linked in vivo to significant changes in drug-induced cytotoxicity. We selected predicted (TF, Drug) pairs that reflect a diversity of regulatory support, shown in Table 4, A and B. For instance, we selected 10 of the 11 (TF, Drug) pairs identified uniquely by eQTL + M analysis (we omitted GTF2F1*)* and two of the three (TF, Drug) pairs predicted by all three analyses: eQTL + M, eQTL, and eQTM. Additionally, we selected the top four pairs by LLR in the eQTL + M analysis that were also supported by eQTM-only analysis. We also included a high-scoring association from eQTM-only analysis that scored poorly in the eQTL + M analysis (i.e., LLR < 3). We did not experimentally pursue eQTL-only predictions, as such validations were reported in our previous work (Hanson et al. 2015) and our focus here was primarily on the eQTL + M mode.

**Table 4.**
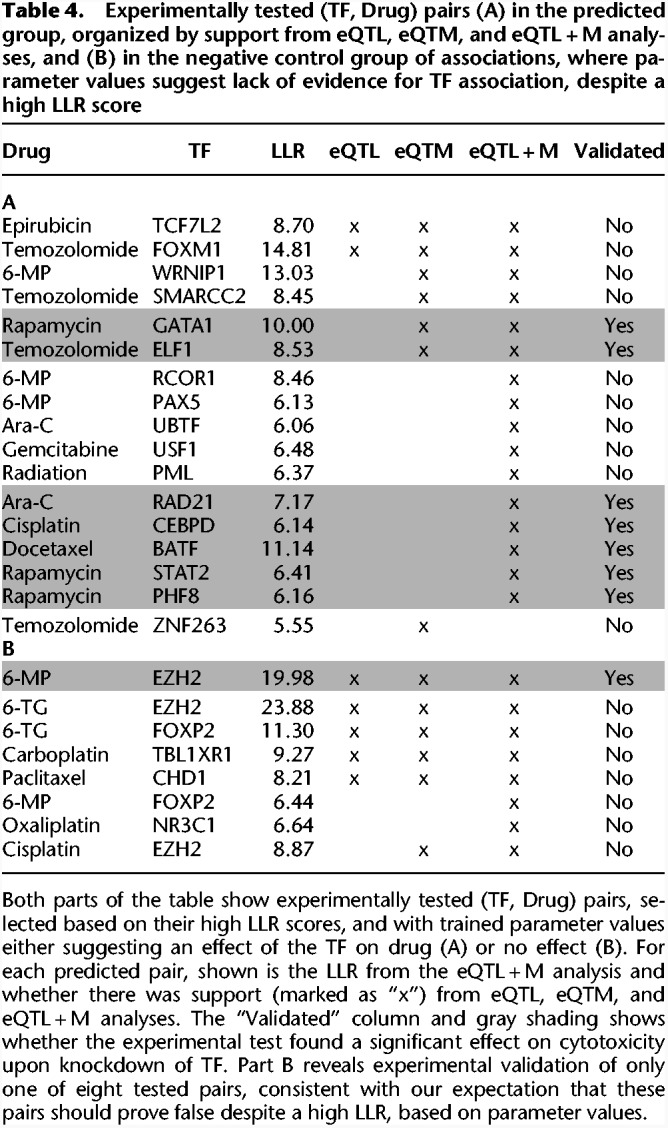
Experimentally tested (TF, Drug) pairs (A) in the predicted group, organized by support from eQTL, eQTM, and eQTL + M analyses, and (B) in the negative control group of associations, where parameter values suggest lack of evidence for TF association, despite a high LLR score

The 17 selected (TF, Drug) pairs were found by pGENMi using learned parameters consistent with our expectation, i.e., where presence of regulatory evidence relating a TF to a gene makes the gene more likely to be associated with the drug (parameters *w*_*i*_ > 0, for *i* ≠ 0) (see Methods). For some (TF, Drug) pairs, the maximum likelihood estimation assigned negative values to both of the *w*_*i*_ parameters, while yielding a high LLR score, marking a departure from the null model. However, such negative weights indicate that the *absence* of regulatory evidence relating the TF to the gene is a better marker for linking genes to phenotype, meaning that the TF is unlikely to be a regulator of the phenotype. We included eight such (TF, Drug) associations as a negative control group, pushing the total number of experimental pairs to 25. We expected these eight predictions to prove false, since pGENMi considered them as statistically interesting but in a manner inconsistent with its mechanistic underpinnings.

**Figure 3.**
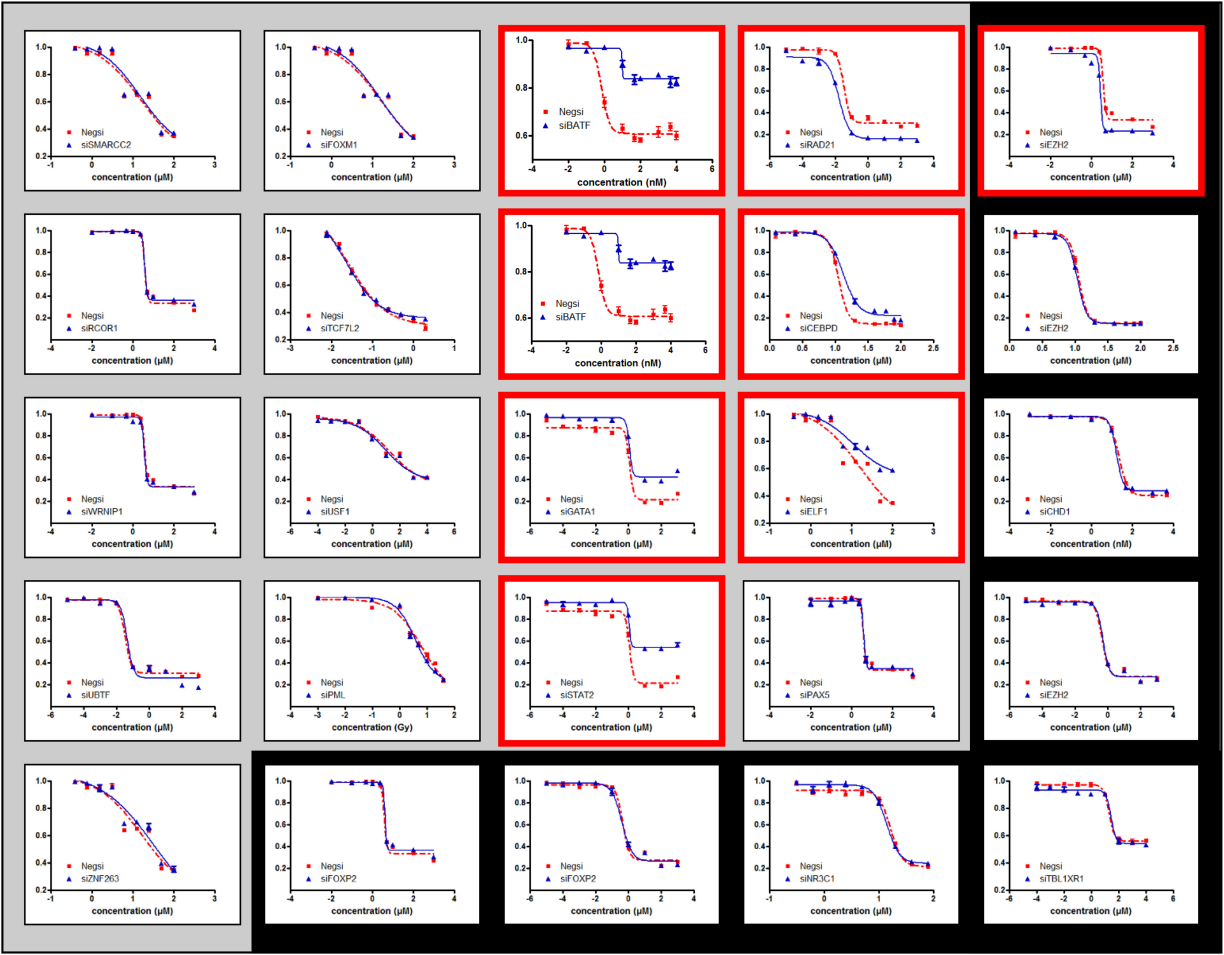
All 25 experimentally tested (TF, Drug) dosage-response curves. Red outlines indicate significant shifts in cytotoxicity between siRNA negative and siRNA TF conditions. Curves with a gray background are eQTL + M predictions, while those with a black background are negative controls. We confirmed seven out of 17 predictions and only one out of eight negative controls.

Though we largely utilized lymphoblastoid cell lines data for our association analysis, we performed siRNA knockdown experiments in several different cell lines to demonstrate the generalizability of our results beyond LCLs. Based on clinical relevance, the human triple negative breast cancer MDA-MB-231 cells were chosen to test anthracyclines, taxanes, platinums, gemcitabine, radiation, and rapamycin cytotoxicity, because these drugs are typically administered as first-line therapy for triple negative breast cancer. We used a human leukemia Jurkat cell line to test 6-MP, 6-TG, and Ara-C since these drugs are used to treat leukemia. Temozolomide is the first-line therapy for glioblastoma multiforme; we therefore used human glioma U251 cells to validate the TFs associated with temozolomide response. The siRNA knockdowns were performed for the 25 (TF, Drug) pairs shown in Table 4, A and B for the eQTL + M predicted and negative control associations, respectively. Cytotoxicity graphs of all knockdowns are shown in Figure 3, while those separated by drug are shown in Supplemental Figures S1–S13. Overall, seven of 17 (∼41%) predicted associations (Table 4A) were validated in this way, compared to one of eight (∼12%) negative control associations (Table 4B). This overall precision of 41% in our own validations is similar to the corresponding precision observed across all drugs (Table 2, “Direct” evidence). Additionally, the precision on the 10 unique eQTL + M predicted associations was 50% (five of 10), compared to 0% (zero of two) of negative control associations unique to eQTL + M mode; this indicates that pGENMi is able to combine multiple lines of regulatory evidence to make novel regulatory predictions that may be unreported when considering each line of evidence in isolation. With respect to the (TF, Drug) pair predicted uniquely from eQTM analysis (and not predicted eQTL- or eQTLM-only), we failed to validate it in our experiments. Nevertheless, we believe that our experimental validation demonstrates the utility of pGENMi predictions overall and especially when combining multiple sources of regulatory information to learn novel associations.

### pGENMi produces associations distinct from simple baseline methods

The pGENMi eQTL + M analysis, with results restricted to those with an LLR ≥ 3, produced 90 (TF, Drug) associations. As no such algorithm other than GENMi (Hanson et al. 2015) exists for associating TF *cis*-regulatory influence with drug response, and even GENMi is incapable of handling both genotype and methylation information simultaneously, there were no obvious reported baseline methods to which we could compare pGENMi, so we constructed two simple baselines. In the first, a TF is considered associated with a drug if the TF's expression correlates significantly with cytotoxicity, an approach recently used for identifying genes associated with drug response (Rees et al. 2016). This baseline approach reported 121 associations at FDR 0.1 (chosen so that the number of reported associations is similar to that of pGENMi). However, these associations were mostly distinct from those reported by pGENMi; between these 121 associations and the 90 associations reported by pGENMi, only five associations (hypergeometric *P*-value = 0.19) were shared. Similar observations of complementarity between results were made when using other thresholds of significance (see Supplemental Note S3).

In a second baseline method, a TF is associated with a drug if the top drug response genes are enriched for strong nearby ChIP peaks of that TF. Performing a hypergeometric test between the top 500 target genes of a TF, based on the maximum ChIP score in the 50-kb upstream region of each gene, and the top 500 drug response genes from a TWAS analysis yielded 126 (TF, Drug) pairs at an FDR of 0.1 (correction performed per drug). Similar to the first baseline, only two were shared with the pGENMi approach. The results of both baseline analyses demonstrate that pGENMi reveals novel insights into regulatory mechanisms of drug response, based on *cis*-regulatory analysis, that may not be obtained from the current approach of expression-phenotype correlations or from analyses that identify gene targets purely on the basis of the distribution of strong ChIP peaks.

### Top associations of pGENMi significantly overlap with GENMi, but the majority are novel

pGENMi formalizes the statistical procedure of GENMi (Hanson et al. 2015) and extends it to handle multiple regulatory evidences. As such, it is fair to ask to what extent these two related procedures produce similar results. We therefore ran GENMi to test the enrichment between genes ranked by their TWAS *P*-value pertaining to a given drug and genes with either *cis*-eQTL or *cis*-eQTM evidence for that TF. Comparing the top 90 (TF, Drug) pairs by *P*-value produced by GENMi and the top 90 (TF, Drug) pairs produced by pGENMi using both eQTL and eQTM evidence reveals an overlap of 24 (TF, Drug) pairs (hypergeometric test *P*-value 1.1 × 10^−17^), corresponding to a Jaccard coefficient of 15.4%, shown in Table 5. Of these 24 pairs, 10 showed direct validation either through our experiments or in the literature. While a strong overlap between the top associations from these two related methods is expected, it also underscores that pGENMi finds several associations—66 of the top 90—that were not reported by GENMi using the same rank threshold (47 were ranked below 300, out of 3000, by GENMi) and of which 21 were validated via siRNA. This complementarity arises due to the difference of models used in the two analyses, since the same data were utilized.

**Table 5.**
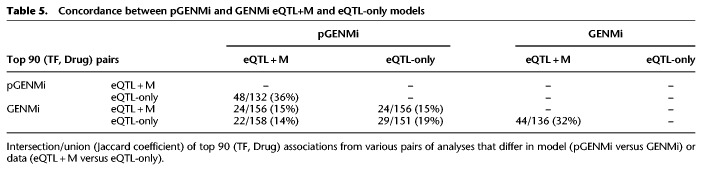
Concordance between pGENMi and GENMi eQTL+M and eQTL-only models

Comparison of GENMi and pGENMi results (top 90 pairs) obtained from eQTL-only or eQTL + M data, in all four combinations, revealed an overlap of 22–29 pairs (Table 5), and substantial complementarity. The commonalities were much greater when results from eQTL + M analysis were compared to those from eQTL-only analysis, using the same method, than when pGENMi and GENMi were compared using the same data (Table 5). For example, the top 90 predictions by pGENMi with eQTL + eQTM data and by pGENMi with eQTL data share 48 associations, while the top 90 predictions by pGENMi with eQTL data and by GENMi with eQTL data share only 29 associations. Thus, roughly speaking, a greater difference in predicted associations arises in changing the model (from GENMi to pGENMi) than in adding eQTM data to pGENMi analysis (from pGENMi eQTL-only to pGENMi eQTL + M).

In the absence of a reliable benchmark of true associations and given the impracticality of extensive literature-based assessment of each of the analyses reported in Table 5, it is difficult to argue empirically if one analysis is more accurate than another. However, it is fair to conclude that the majority of the top associations reported by pGENMi are exclusive to it in comparison with results from GENMi, regardless of the mode of analysis.

### Database of (TF, Drug) associations

We made available as an online resource all (TF, Drug) associations validated using siRNA or overexpression experiments in this study as well as those found to be similarly validated in our survey of the literature (Supplemental Note S1–S2), the GENMi study (Hanson et al. 2015), and a related work that performs the same experimental validations (Emad et al. 2017). This resource is available at veda.cs.uiuc.edu/pgenmi. We believe this to be the first such catalog of experimentally validated (TF, Drug) pairs.

## Discussion

In this work, we developed a principled probabilistic graphical model for inferring TFs that regulate drug response variation among individuals. We demonstrated a high rate of success in validating our associations through a comprehensive literature survey and in vivo experimental validation and have collated the validations from our and other studies into a (TF, Drug) association database.

While we view the pGENMi model as a major improvement over our previous proof-of-principle approach, GENMi (Hanson et al. 2015), the significance of the performance differences between the models on this data are difficult to assess objectively. While there is a strong concordance between the two algorithms (24 out of the top 90 pairs from pGENMi and GENMi eQTL + M models) and a high percentage validate (10/24, ∼ 42%), the complementary pairs exclusive to pGENMi also validate similarly (21/66, ∼ 31%). Additionally, it is difficult to ascertain estimates of the validation rate of the complementary GENMi pairs as the database was predominantly curated using the top 90 pairs from pGENMi eQTL + M models. Those entries in the database corresponding to GENMi were cultivated from a literature analysis of only the top 20 GENMi pairs and experimental data exclusive to only four drugs. As a result, quantifying the difference in overall effectiveness of these two approaches will require further experimentation, which we leave to future analyses.

Though pGENMi is similar to its predecessor, it also bears similarity to existing models for data integration. pGENMi somewhat resembles the GPA model (Chung et al. 2014), except that GPA, by modeling SNPs instead of genes as latent variables, applies primarily to GWAS data analysis (see Supplemental Note S3). Furthermore, we use an empirical Bayesian approach similarly used to prioritize SNPs using annotations (Gaffney et al. 2012) and Battle and coworkers’ latent regulatory variant model (LRVM) (Battle et al. 2014). The pGENMi model, although unique in the way that it combines *cis*-regulatory information with interindividual covariation in gene expression and drug response, is not wholly unique in identifying TFs relevant to phenotype. There exist categories of algorithms dedicated to implicating transcriptional mechanisms or regulatory networks specific to certain phenotypes (Geeven et al. 2012; Qi and Michoel 2012; Maier et al. 2015). The main distinction of the pGENMi approach is that it uses data on population-level variations in genotype, epigenotype, gene expression, and phenotype to uncover those mechanisms. Its findings are thus expected to be more relevant to what underlies individual differences in phenotype, as opposed to a more general catalog that includes TFs that may be relevant to the phenotype but not so much to its individual-level variations. On a relatively minor note, the above methods for reconstructing context-specific transcriptional regulatory networks require data on discrete cellular conditions (disease versus nondisease state, for instance) and are thus distinct from pGENMi, which focuses on interindividual continuous variation in cellular phenotype. TF associations with drugs can also be inferred from general studies that prioritize genes related to drug response based on prior functional networks (Morrison et al. 2005; Chen et al. 2012; Emad et al. 2017) or based on observed or imputed gene expression alone (Barretina et al. 2012; Basu et al. 2013; Gusev et al. 2016; Rees et al. 2016). These approaches are not focused on identifying regulators of phenotype based on *cis*-regulatory evidence.

Validation rates of pGENMi predictions, based on a literature search or our own experiments, were around 40% overall, which is far from perfect, and highly variable across drugs. Exploring possible reasons for false positives, we noted great disparity among drugs in terms of the number of significant TWAS genes (i.e., whose expression is associated with phenotypic variation) and that pGENMi predicts more associations, with lower validation rates, for drugs with more TWAS genes (Supplemental Note S3; Supplemental Tables S6, S7). In some cases, extensive cobinding of a pair of TFs may also have led to false positives, with peaks of the true regulator mistakenly providing evidence in favor of the cobinding TF's influence on several TWAS genes (Supplemental Note S3). With regard to experimental validation, some (TF, Drug) pairs did not validate in vivo because of the assay design; (FOXM1, temozolomide) and (PML, radiation) were validated directly in tissues according to the literature, but both failed to validate in vivo in cell lines. Because (TF, Drug) pairs may be context (cell line)-specific, failure to validate in certain cell lines cannot be interpreted necessarily as a rejection of the relationship altogether.

pGENMi can be improved in a number of ways; for instance, our model makes the implicit assumption that a latent variable *z*_*g*_ representing a gene's involvement in mediating the TF's influence on phenotype is independent across genes; however, coregulated gene sets break this assumption, and it would be more reflective of the underlying biology to model a network of latent variables *z*_*g*_ instead of treating each gene independently. Our choice of modeling the *P*-values of gene expression-phenotype correlation as arising from a Beta distribution avoids the use of arbitrary thresholds on the strength of this relationship, but we believe there may be less restrictive ways to explicitly model phenotype-expression relationships. Although we integrated more types of data in this work than typical studies that directly relate gene expression to drug response data (Gusev et al. 2016; Rees et al. 2016), the model allows further extensions. For instance, incorporating the impact of various histone modifications and other epigenetic marks will be an interesting endeavor if such variants are profiled in the cell line panel. Additionally, it may be wise to consider in vivo assays to validate the mediators of drug response, in addition to the TFs themselves, in the future. Despite these various areas of improvement, the pGENMi methodology was successful in integrating both genetic and epigenetic variation to elucidate the regulatory association between a TF and drug, as evidenced by our 50% precision in validating those predictions only eQTL + M reported. We hope this methodology can serve as a blueprint for future endeavors that aim to elucidate the regulatory basis of disease etiology using multiple molecular profiles of variation.

The pGENMi framework need not be restricted to the analysis of the variation of drug response. It may be easily adapted for use in other situations where one seeks to link a phenotype to a set of gene properties, e.g., regulation by a TF, membership in a pathway, involvement in a biological process, etc., while using gene expression as an intermediate variable. The relationship between gene expression and phenotype may be quantified by differential expression *P*-values if, for instance, the phenotype is binary, as in case versus control studies or in before versus after treatment studies.

## Methods

### Data acquisition

We obtained data on single nucleotide polymorphisms (SNPs), 3′ mRNA probe expression, and CpG methylation status across 284 Epstein-Barr Virus (EBV)-transformed lymphoblastoid cell lines (LCL) from the Coriell Cell Repository. The ethnicities of the cohort decomposed along the following three broad ethnic lines: 95 Han-Chinese American (HCA), 96 Caucasian American (CA), and 93 African American (AA). The sequenced genotype data resulted in 1,362,404 germ line SNPs, each with minor allele frequency (MAF) < 5%, genotype rate > 95%, and in Hardy-Weinberg equilibrium. Imputation analysis (see Supplemental Note S4) of this initial set of genotyped SNPs resulted in 11,256,504 SNPs. Gene expression data consisted of 54,613 Affymetrix U133 Plus 2.0 GeneChip probes, transformed using log_2_ GC Robust Multi-Array Averaging (GC-RMA). Genotyped SNP and gene expression data are available in the NCBI Gene Expression Omnibus (GEO) (http://www.ncbi.nlm.nih.gov/geo) under SuperSeries accession no. GSE24277 and were originally published in a study by Niu et al. (2010).

We used data on 444,797 methylation marks, originally published by Heyn et al. (2013), available in NCBI GEO under SuperSeries accession no. GSE36369. As a representation of methylation status, we used the “beta” value, which encodes methylation status within [0, 1], where 0 and 1 correspond to total absence and presence of the mark, respectively. Information on preprocessing and analysis of this data, including SNP imputation, population stratification, gene expression processing, and regression design, among others, resides in Supplemental Note S4.

Drug response data was derived from dosage-response curves of 24 cytotoxic drugs: 6-MP, 6-TG, Ara-C, arsenic, carboplatin, cisplatin, cladribine, docetaxel, doxorubicin, epirubicin, everolimus, fludarabine, gemcitabine, hypoxia, metformin, MPA, methotrexate, NAPQI, oxaliplatin, paclitaxel, radiation, rapamycin, triciribine, and temozolomide. Each response curve was summarized by an EC50 value (drug concentration at which half the original LCL population survived treatment). These data were most recently analyzed by Hanson et al. (2015) and are available at the following location: veda.cs.uiuc.edu/cytotoxicity. Full information regarding the experimental validation design, data, methodology, and statistical analysis is in Supplemental Note S5.

Experimental data on TF binding were obtained from the ENCODE project (The ENCODE Project Consortium 2011). The union of clustered ChIP (v3) tracks of 161 TFs across 91 cell lines formed a single track for each TF, representing a TF composite regulatory profile reflective of activity across a wide variety of cellular contexts. The data for these clustered ENCODE cell lines are located at the following URL: http://hgdownload.cse.ucsc.edu/goldenpath/hg19/encodeDCC/wgEncodeRegTfbsClustered. We remove clustered peaks that were likely the artifact of high occupancy target (HOT) regions, as in the GENMi analysis (Hanson et al. 2015). Contrary to that work, we exempted the following 13 TFs from the full 161: general TFs *(*POLR2A, POLR3A, POLR3G, TBP) and those for which no eQTL or eQTM SNPs (*P* < 0.05) were detected within ChIP peaks (BDP1, BRCA1, BRF1, ELK1, ELK4, ESRRA, HSF1, KDM5A, NELFE). Further processing of this data, including the removal of high occupancy target (HOT) regions, is described in Supplemental Note S4.

### TWAS, eQTL, and eQTM analysis

Each of the following regression analyses controlled for the following covariates: sex, age, batch, and population axes of variation derived from EIGENSTRAT. Transcript-wide association analysis, or TWAS (Cloney 2016), and eQTL analysis were performed following Hanson et al. (2015) (Supplemental Note S4). To perform TWAS, we computed partial regression coefficient *P*-values between gene expression and drug EC50 values across the 284 LCLs, for each (gene, drug) pair. For eQTL analysis, we calculated partial regression coefficient *P*-values between each gene's expression and the genotype (measured by allelic dosing of 0,1,2) of each SNP in its *cis*-regulatory (50-kb upstream) region. To avoid the statistical artifacts of linkage disequilibrium, we preserved only the most significant eQTL-SNP in each gene's *cis*-regulatory region. For eQTM analysis, we repeated the eQTL analysis while substituting SNP genotypes with methylation status, a continuous variable between 0 and 1 representing the probability of the presence of a CpG methylation, and thus computed eQTM (methylation to gene expression regression) *P*-values, again retaining only the most significant eQTM in the *cis*-regulatory region of a gene.

### Model structure and learning

#### Probabilistic graphical model

The pGENMi “plate diagram” is shown in Figure 1, which models the association between a specific TF and a phenotype. There are four variable types (*p*,*z*_*g*_,*r*_*gm*_,*H*) and two parameter types (*α*,*w*_*m*_). The variable *r*_*gm*_ is a binary indicator variable representing whether a gene *g* has a particular kind of evidence (e.g., *cis*-eQTL, *cis*-eQTM, etc.) of regulation by the TF. Here, a *cis*-eQTL evidence is defined, following Hanson et al. (2015), as the presence of an eQTL within a ChIP peak of the TF, in the 50-kb upstream region of the gene. Likewise, a *cis*-eQTM evidence is the presence of an eQTM within a TF ChIP peak in the gene's 50-kb upstream region. Thus, if *r*_*gm*_ = 1 for some gene *g* and “m” represents *cis*-eQTM evidence, we interpret this as evidence that a change of TF binding at the ChIP peak is brought about by or related to the observed individual variation in methylation status at the eQTM, which in turn explains the latter's correlation with gene expression.

A rectangle labeled with an *M* engulfs *r*, indicating that there are *M* such kinds of regulatory evidence for each gene *g*: *r*_*g*1_, *r*_*g*2_, … , *r*_*gM*_. Each *r*_*gm*_ variable in a plate connects to the same *z*_*g*_ variable. This latter variable is binary and latent (unshaded) and indicates whether expression of a gene *g* is related to the phenotype. A rectangle labeled *G* encapsulates *z*_*g*_ and *r*_*gm*_, indicating that there is a latent variable *z*_*g*_ and regulatory evidence vector *r*_*gm*_ for each of *G* genes. The state of *z*_*g*_ is determined probabilistically as

$$P(Z_{g}=1|r_{g1}\ldots r_{gM},w_{0}\ldots w_{M})=\frac{1}{1+\exp(-(w_{0}+w_{1}r_{g1}+w_{2}r_{g2}+\cdots +w_{M}r_{gM}))}.$$

Here, the various evidences *r*_*gm*_ for the regulation of gene *g* by the TF are combined in a logistic function, each weighted by a coefficient *w*_*m*_. Thus, a weighting of separate regulatory evidences determines whether a gene *g* is related to the phenotype. The variable *p* is a continuous variable in the range [0,1], representing the observed TWAS *P*-value (details above) of the association between gene expression and phenotype. If a gene is related to the phenotype (*z*_*g*_ = 1), then we model *p* to follow a *Beta*(*α*,1) distribution biased toward small (significant) *P*-values; *α* is a shape parameter indicating the strength of the bias, constrained to the range (0,1], with *α* = 1 equivalent to a uniform distribution over *P*-values. However, if a gene is unrelated to phenotype (*z*_*g*_ = 0), we expect its expression-phenotype correlation *P*-value to be uniformly distributed over [0,1]. These modeling assumptions are summarized below:

$$p_{g}∼\{\begin{array}{cc} Unif\;(0,1) & \mathrm{if}\;z_{g}=0\\ \beta (\alpha ,1) & if\;z_{g}=1 \end{array}.$$

The binary variable *H* indicates the hypothesis to be tested. When *H* = 1, the model tries to find the best assignments to **w** and *α*; this is the alternative hypothesis or *H*_1_. When *H* = 0, the model only trains parameters *w*_0_ and *α*, and the weights *w*_1_, …, *w*_*M*_ are removed from the model entirely. In this case, the model tries to explain the observed TWAS *P*-values without any regulatory evidences at all; this is the null hypothesis, or *H*_0_. We derive a score for how much better the alternative hypothesis explains the data **y**:

$$LLR=\log_{2}P(y|A,H=1)-\log_{2}P(y|A,H=0).$$

The pGENMi method uses the log likelihood ratio (LLR), computed for each TF separately, to rank TFs by their predicted association with the phenotype.

#### Parameter estimation

The model uses the expectation maximization algorithm to find assignments to the parameters, **W** = [*w*_0_, *w*_1_, …, *w*_*M*_] and *α*, such that the likelihood of the observed TWAS *P*-values, ***p*** = [*p*_1_, …, *p*_*G*_], is maximized (Supplemental Note S6). The optimization imposes no constraints on the parameters, and thus the weights of regulatory evidences (**w**) may be trained to negative values, in effect rewarding genes that have regulatory evidence but no expression-phenotype correlation. As this is not consistent with our goals, pGENMi invokes a post-processing step to disregard such spurious TF-phenotype associations.

### Software availability

Source code for pGENMi is available in Supplemental Code S1 and at the following: https://github.com/knoweng/pgenmi.

## Supplementary Material

Supplemental Material

## References

[GR227066HANC1] Barretina J, Caponigro G, Stransky N, Venkatesan K, Margolin AA, Kim S, Wilson CJ, Lehar J, Kryukov GV, Sonkin D, 2012 The Cancer Cell Line Encyclopedia enables predictive modelling of anticancer drug sensitivity. Nature 483: 603–607. 22460905 10.1038/nature11003 PMC3320027

[GR227066HANC2] Basu A, Bodycombe NE, Cheah JH, Price EV, Liu K, Schaefer GI, Ebright RY, Stewart ML, Ito D, Wang S, 2013 An interactive resource to identify cancer genetic and lineage dependencies targeted by small molecules. Cell 154: 1151–1161. 23993102 10.1016/j.cell.2013.08.003 PMC3954635

[GR227066HANC3] Battle A, Mostafavi S, Zhu X, Potash JB, Weissman MM, McCormick C, Haudenschild CD, Beckman KB, Shi J, Mei R, 2014 Characterizing the genetic basis of transcriptome diversity through RNA-sequencing of 922 individuals. Genome Res 24: 14–24. 24092820 10.1101/gr.155192.113 PMC3875855

[GR227066HANC4] Bavelloni A, Faenza I, Aluigi M, Ferri A, Toker A, Maraldi NM, Marmiroli S. 2000 Inhibition of phosphoinositide 3-kinase impairs pre-commitment cell cycle traverse and prevents differentiation in erythroleukaemia cells. Cell Death Differ 7: 112–117. 10713726 10.1038/sj.cdd.4400591

[GR227066HANC5] Bernstein BE, Stamatoyannopoulos JA, Costello JF, Ren B, Milosavljevic A, Meissner A, Kellis M, Marra MA, Beaudet AL, Ecker JR, 2010 The NIH Roadmap Epigenomics Mapping Consortium. Nat Biotechnol 28: 1045–1048. 20944595 10.1038/nbt1010-1045 PMC3607281

[GR227066HANC6] Chen X, Jiang W, Wang Q, Huang T, Wang P, Li Y, Chen X, Lv Y, Li X. 2012 Systematically characterizing and prioritizing chemosensitivity related gene based on Gene Ontology and protein interaction network. BMC Med Genomics 5: 43. 23031817 10.1186/1755-8794-5-43 PMC3532125

[GR227066HANC7] Chung D, Yang C, Li C, Gelernter J, Zhao H. 2014 GPA: a statistical approach to prioritizing GWAS results by integrating pleiotropy and annotation. PLoS Genet 10: e1004787. 25393678 10.1371/journal.pgen.1004787 PMC4230845

[GR227066HANC8] Cloney R. 2016 Complex traits: integrating gene variation and expression to understand complex traits. Nat Rev Genet 17: 194. 26900024 10.1038/nrg.2016.18

[GR227066HANC9] Corradin O, Scacheri PC. 2014 Enhancer variants: evaluating functions in common disease. Genome Med 6: 85. 25473424 10.1186/s13073-014-0085-3 PMC4254432

[GR227066HANC10] Emad A, Cairns J, Kalari KR, Wang L, Sinha S. 2017 Knowledge-guided gene prioritization reveals new insights into the mechanisms of chemoresistance. Genome Biol 18: 153. 28800781 10.1186/s13059-017-1282-3 PMC5554409

[GR227066HANC32] The ENCODE Project Consortium. 2011 A user's guide to the encyclopedia of DNA elements (ENCODE). PLoS Biol 9: e1001046. 21526222 10.1371/journal.pbio.1001046 PMC3079585

[GR227066HANC11] The ENCODE Project Consortium. 2012 An integrated encyclopedia of DNA elements in the human genome. Nature 489: 57–74. 22955616 10.1038/nature11247 PMC3439153

[GR227066HANC12] Faiao-Flores F, Alves-Fernandes DK, Pennacchi PC, Sandri S, Vicente ALSA, Scapulatempo-Neto C, Vazquez VL, Reis RM, Chauhan J, Goding CR, 2017 Targeting the hedgehog transcription factors GLI1 and GLI2 restores sensitivity to vemurafenib-resistant human melanoma cells. Oncogene 36: 1849–1861. 27748762 10.1038/onc.2016.348 PMC5378933

[GR227066HANC13] Fontaine F, Overman J, Francois M. 2015 Pharmacological manipulation of transcription factor protein-protein interactions: opportunities and obstacles. Cell Regen (Lond) 4: 2. 25848531 10.1186/s13619-015-0015-x PMC4365538

[GR227066HANC14] Forbes SA, Beare D, Gunasekaran P, Leung K, Bindal N, Boutselakis H, Ding M, Bamford S, Cole C, Ward S, 2015 COSMIC: exploring the world's knowledge of somatic mutations in human cancer. Nucleic Acids Res 43: D805–D811. 25355519 10.1093/nar/gku1075 PMC4383913

[GR227066HANC15] Gaffney DJ, Veyrieras JB, Degner JF, Pique-Regi R, Pai AA, Crawford GE, Stephens M, Gilad Y, Pritchard JK. 2012 Dissecting the regulatory architecture of gene expression QTLs. Genome Biol 13: R7. 22293038 10.1186/gb-2012-13-1-r7 PMC3334587

[GR227066HANC16] Gariboldi MB, Ravizza R, Molteni R, Osella D, Gabano E, Monti E. 2007 Inhibition of Stat3 increases doxorubicin sensitivity in a human metastatic breast cancer cell line. Cancer Lett 258: 181–188. 17920763 10.1016/j.canlet.2007.08.019

[GR227066HANC17] Geeven G, van Kesteren RE, Smit AB, de Gunst MC. 2012 Identification of context-specific gene regulatory networks with GEMULA—gene expression modeling using LAsso. Bioinformatics 28: 214–221. 22106333 10.1093/bioinformatics/btr641

[GR227066HANC18] Gomes AR, Zhao F, Lam EW. 2013 Role and regulation of the forkhead transcription factors FOXO3a and FOXM1 in carcinogenesis and drug resistance. Chin J Cancer 32: 365–370. 23706767 10.5732/cjc.012.10277 PMC3845605

[GR227066HANC19] Gregori-Puigjane E, Setola V, Hert J, Crews BA, Irwin JJ, Lounkine E, Marnett L, Roth BL, Shoichet BK. 2012 Identifying mechanism-of-action targets for drugs and probes. Proc Natl Acad Sci 109: 11178–11183. 22711801 10.1073/pnas.1204524109 PMC3396511

[GR227066HANC20] Gusev A, Ko A, Shi H, Bhatia G, Chung W, Penninx BWJH, Jansen R, de Geus EJC, Boomsma DI, Wright FA, 2016 Integrative approaches for large-scale transcriptome-wide association studies. Nat Genet 48: 245–252. 26854917 10.1038/ng.3506 PMC4767558

[GR227066HANC21] Hanson C, Cairns J, Wang L, Sinha S. 2015 Computational discovery of transcription factors associated with drug response. Pharmacogenomics J 16: 573–582. 26503816 10.1038/tpj.2015.74 PMC4848185

[GR227066HANC22] Heyn H, Moran S, Hernando-Herraez I, Sayols S, Gomez A, Sandoval J, Monk D, Hata K, Marques-Bonet T, Wang L, 2013 DNA methylation contributes to natural human variation. Genome Res 23: 1363–1372. 23908385 10.1101/gr.154187.112 PMC3759714

[GR227066HANC23] Iorio F, Bosotti R, Scacheri E, Belcastro V, Mithbaokar P, Ferriero R, Murino L, Tagliaferri R, Brunetti-Pierri N, Isacchi A, 2010 Discovery of drug mode of action and drug repositioning from transcriptional responses. Proc Natl Acad Sci 107: 14621–14626. 20679242 10.1073/pnas.1000138107 PMC2930479

[GR227066HANC24] Jones MJ, Fejes AP, Kobor MS. 2013 DNA methylation, genotype and gene expression: who is driving and who is along for the ride? Genome Biol 14: 126. 23899167 10.1186/gb-2013-14-7-126 PMC4054606

[GR227066HANC25] Koller D, Friedman N. 2009 Probabilistic graphical models: principles and techniques. Adaptive computation and machine learning series. The MIT Press, Cambridge, MA.

[GR227066HANC26] Lee D, Gorkin DU, Baker M, Strober BJ, Asoni AL, McCallion AS, Beer MA. 2015 A method to predict the impact of regulatory variants from DNA sequence. Nat Genet 47: 955–961. 26075791 10.1038/ng.3331 PMC4520745

[GR227066HANC27] Long MD, van den Berg PR, Russell JL, Singh PK, Battaglia S, Campbell MJ. 2015 Integrative genomic analysis in K562 chronic myelogenous leukemia cells reveals that proximal NCOR1 binding positively regulates genes that govern erythroid differentiation and Imatinib sensitivity. Nucleic Acids Res 43: 7330–7348. 26117541 10.1093/nar/gkv642 PMC4551916

[GR227066HANC28] Madian AG, Wheeler HE, Jones RB, Dolan ME. 2012 Relating human genetic variation to variation in drug responses. Trends Genet 28: 487–495. 22840197 10.1016/j.tig.2012.06.008 PMC3448823

[GR227066HANC29] Maier EJ, Haynes BC, Gish SR, Wang ZA, Skowyra ML, Marulli AL, Doering TL, Brent MR. 2015 Model-driven mapping of transcriptional networks reveals the circuitry and dynamics of virulence regulation. Genome Res 25: 690–700. 25644834 10.1101/gr.184101.114 PMC4417117

[GR227066HANC30] Moen EL, Godley LA, Zhang W, Dolan ME. 2012 Pharmacogenomics of chemotherapeutic susceptibility and toxicity. Genome Med 4: 90. 23199206 10.1186/gm391 PMC3580423

[GR227066HANC31] Morrison JL, Breitling R, Higham DJ, Gilbert DR. 2005 GeneRank: using search engine technology for the analysis of microarray experiments. BMC Bioinformatics 6: 233. 16176585 10.1186/1471-2105-6-233 PMC1261158

[GR227066HANC33] Nelson MR, Johnson T, Warren L, Hughes AR, Chissoe SL, Xu C-F, Waterworth DM. 2016 The genetics of drug efficacy: opportunities and challenges. Nat Rev Genet 17: 197–206. 26972588 10.1038/nrg.2016.12

[GR227066HANC34] Niu N, Qin Y, Fridley BL, Hou J, Kalari KR, Zhu M, Wu TY, Jenkins GD, Batzler A, Wang L. 2010 Radiation pharmacogenomics: a genome-wide association approach to identify radiation response biomarkers using human lymphoblastoid cell lines. Genome Res 20: 1482–1492. 20923822 10.1101/gr.107672.110 PMC2963812

[GR227066HANC35] Papavassiliou KA, Papavassiliou AG. 2016 Transcription factor drug targets. J Cell Biochem 117: 2693–2696. 27191703 10.1002/jcb.25605

[GR227066HANC36] Price AL, Patterson NJ, Plenge RM, Weinblatt ME, Shadick NA, Reich D. 2006 Principal components analysis corrects for stratification in genome-wide association studies. Nat Genet 38: 904–909. 16862161 10.1038/ng1847

[GR227066HANC37] Qi J, Michoel T. 2012 Context-specific transcriptional regulatory network inference from global gene expression maps using double two-way *t*-tests. Bioinformatics 28: 2325–2332. 22962443 10.1093/bioinformatics/bts434

[GR227066HANC38] Redell MS, Tweardy DJ. 2006 Targeting transcription factors in cancer: challenges and evolving strategies. Drug Discov Today Technol 3: 261–267. 24980527 10.1016/j.ddtec.2006.09.010

[GR227066HANC39] Redmond AM, Carroll JS. 2009 Defining and targeting transcription factors in cancer. Genome Biol 10: 311. 19664186 10.1186/gb-2009-10-7-311 PMC2728525

[GR227066HANC40] Rees MG, Seashore-Ludlow B, Cheah JH, Adams DJ, Price EV, Gill S, Javaid S, Coletti ME, Jones VL, Bodycombe NE, 2016 Correlating chemical sensitivity and basal gene expression reveals mechanism of action. Nat Chem Biol 12: 109–116. 26656090 10.1038/nchembio.1986 PMC4718762

[GR227066HANC41] Schaub MA, Boyle AP, Kundaje A, Batzoglou S, Snyder M. 2012 Linking disease associations with regulatory information in the human genome. Genome Res 22: 1748–1759. 22955986 10.1101/gr.136127.111 PMC3431491

[GR227066HANC42] Schenone M, Dancik V, Wagner BK, Clemons PA. 2013 Target identification and mechanism of action in chemical biology and drug discovery. Nat Chem Biol 9: 232–240. 23508189 10.1038/nchembio.1199 PMC5543995

[GR227066HANC43] Shahzad MM, Mangala LS, Han HD, Lu C, Bottsford-Miller J, Nishimura M, Mora EM, Lee JW, Stone RL, Pecot CV, 2011 Targeted delivery of small interfering RNA using reconstituted high-density lipoprotein nanoparticles. Neoplasia 13: 309–319. 21472135 10.1593/neo.101372 PMC3071079

[GR227066HANC44] Ward LD, Kellis M. 2012 Interpreting noncoding genetic variation in complex traits and human disease. Nat Biotechnol 30: 1095–1106. 23138309 10.1038/nbt.2422 PMC3703467

[GR227066HANC45] Welter D, MacArthur J, Morales J, Burdett T, Hall P, Junkins H, Klemm A, Flicek P, Manolio T, Hindorff L, 2014 The NHGRI GWAS catalog, a curated resource of SNP-trait associations. Nucleic Acids Res 42: D1001–D1006. 24316577 10.1093/nar/gkt1229 PMC3965119

[GR227066HANC46] Yang W, Soares J, Greninger P, Edelman EJ, Lightfoot H, Forbes S, Bindal N, Beare D, Smith JA, Thompson IR, 2013 Genomics of Drug Sensitivity in Cancer (GDSC): a resource for therapeutic biomarker discovery in cancer cells. Nucleic Acids Res 41: D955–D961. 23180760 10.1093/nar/gks1111 PMC3531057

[GR227066HANC47] Yeh JE, Toniolo PA, Frank DA. 2013 Targeting transcription factors: promising new strategies for cancer therapy. Curr Opin Oncol 25: 652–658. 24048019 10.1097/01.cco.0000432528.88101.1a

[GR227066HANC48] Zhang N, Wu X, Yang L, Xiao F, Zhang H, Zhou A, Huang Z, Huang S. 2012 FoxM1 inhibition sensitizes resistant glioblastoma cells to temozolomide by downregulating the expression of DNA-repair gene *Rad51*. Clin Cancer Res 18: 5961–5971. 22977194 10.1158/1078-0432.CCR-12-0039 PMC3639123

[GR227066HANC49] Zhou J, Troyanskaya OG. 2015 Predicting effects of noncoding variants with deep learning-based sequence model. Nat Methods 12: 931–934. 26301843 10.1038/nmeth.3547 PMC4768299

