# Supplementary material for: Principled multi-omic analysis reveals gene regulatory mechanisms of phenotype variation
Source: Genome Res. 2018 Aug;28(8):1207–16. doi: 10.1101/gr.227066.117 (PMC6071639; doi:10.1101/gr.227066.117)
Supplement: Supplemental Material [file supp_28_8_1207__index.html]

Principled multi-omic analysis reveals gene regulatory mechanisms of phenotype variation — Supplemental Material 

# Principled multi-omic analysis reveals gene regulatory mechanisms of phenotype variation

## Supplemental Material

- Supplemental\_Material.pdf
- Supplemental\_Code\_S1.zip
