# Supplementary material for: Principled multi-omic analysis reveals gene regulatory mechanisms of phenotype variation
Source: Genome Res. 2018 Aug;28(8):1207–16. doi: 10.1101/gr.227066.117 (PMC6071639; doi:10.1101/gr.227066.117)
Supplement: Supplemental Material [file supp_gr.227066.117_Supplemental_Material.pdf]

Supplemental Material: This file contains supplemental text, figures, and tables in the following order.

1. **Supplemental Note S1**: Literature Validation of eQTL+M (TF, Drug) pairs with LLR >= 4.5
2. **Supplemental Note S2**: Literature Validation of eQTL+M (TF, Drug) pairs with LLR >= 3 by Drug.
3. **Supplemental Note S3**: Supplemental Results and Discussion
4. **Supplemental Note S4**: Data pre-processing and statistical methodology
5. **Supplemental Note S5**: Experimental validation design, data, methodology, and statistical analysis
6. **Supplemental Note S6**: Expectation Maximization Formulation for PGM and Implementation Details
7. **Supplemental Figs. S1 - S14** with Captions
8. **Supplemental Table S1**: Decomposition of the LLR between eQTL, eQTM, and eQTL+M
9. **Supplemental Table S2:** EIGENSTRAT sub-populations
10. **Supplemental Table S3:** Concentration of administered drugs applied during experimental validation.
11. **Supplemental Table S4**: Common Top TFs b/t GM12878 and Composite ChIP Results
12. **Supplemental Table S5**: Common Top Drugs b/t GM12878 and Composite ChIP Results
13. **Supplemental Table S6**: TWAS genes per drug with eQTL+M associations
14. **Supplemental Table S7**: Median/Mean TWAS genes for top and bottom eQTL+M Drugs
15. **References**

Supplemental Note S1: Here, we list and discuss the top (TF, Drug) associations predicted by eQTL+M with an LLR >= 4.5 ranked by strength of evidence and LLR score.

For each (TF, Drug) association, we report several fields. The first is the “Validation Status” field, which indicates whether we consider this association to be direct validation (exhibited usually by TF knockdown) or indirect (exhibited usually by TF mRNA differential expression or binding in response to the drug treatment). The second field, “Evidence”, summarizes the evidence we found for the association. The next field, “LLR”, contains the pGENMi eQTL+M LLR for the association. The following “High Scoring Analyses” field contains the experiments that yielded a significant LLR; for eQTL and eQTM, the LLR threshold was 1.74. Since we only examine significant eQTL+M pairs in this analysis, every association should at least have eQTL+M in this field. The last field is “Observation” which is a paragraph describing literature evidence we found corroborating the (TF, Drug) association or at least providing some evidence as to its validity.

1. **Association: FOXM1 with Temozolomide**

   Validation Status: Direct

   Evidence: TF siRNA and Differential TF Concentration

   LLR: 7.40

   High Scoring Analyses: eQTL+M, eQTL, eQTM

   Observation: Recurrent glioblastoma multiforme (GBM) tumors are characterized by a resistance to chemo and radiotherapy. Recurrent GBM tumors resistant to temozolomide showed higher levels of FOXM1 than primary tumors. Treating GBM cell lines with temozolomide increased expression of FOXM1 and the DNA damage repair gene, RAD51 and showed resistance to temozolomide treatment. Knockdown of FOXM1 via siRNA assays inhibited RAD51 expression and sensitized recurrent GBM to temozolomide. The regulatory relationship between FOXM1 and *RAD51* was further corroborated by ChIP analysis showing a preponderance of FOXM1 binding in the *RAD51* promoter (Zhang et al. 2012).

2. **Association: MEF2C with NAPQI**

   Validation Status: Not Validated (Some Evidence)

   Evidence: Regulatory Partner of Differentially Expressed Drug Target mRNA

   LLR: 7.17

   High Scoring Analyses: eQTL+M, eQTL, eQTM

   Observation: Yeast 2 hybrid experiments showed an interaction between MEF2C and HABP4; further investigation showed that HABP4 inhibits the DNA binding potential of MEF2C. This interaction was confirmed in-vitro using GST-pull down assays and in-vivo rat heart cells by ChIP (Kobarg et al. 2005). A separate study on hepatotoxicity demonstrated that acetaminophen treatment affected *HABP4* expression (Beyer et al. 2007). Given the

interaction between MEF2C and HABP4 and *HABP4's* sensitivity to acetaminophen, we concluded that though there may be an association between MEF2C and NAPQI, we did not have sufficient experimental evidence to call the relationship direct or indirect.

3 - 5. Association: *NFIC*, *CCNT2*, and *UBTF* with NAPQI

Validation Status: Indirect

Evidence: Differential Expression of TF mRNA

LLR: 7.08, 5.67, and 4.80 respectively

High Scoring Analyses: eQTL+M for all, eQTL for *NFIC*, and eQTM for *CCNT2* and *UBTF*

Observation: A multi-center study of the effect of acetaminophen (APAP) toxicity to liver cells showed differential expression of the TFs *NFIC* and *UBTF* to acetaminophen treatment (Beyer et al. 2007). In a separate gene expression analysis of human liver slices, treatment of acetaminophen also induced differential expression *NFIC* and *CCNT2* (Elferink et al. 2011). The association between *NFIC* and APAP toxicity was also established in mouse liver samples (Moffit et al. 2007). Finally, a transcriptomic, proteomic, and metabolomics profile of hepatoma cells with and without APAP demonstrated the differential *CCNT2* expression (Prot et al. 2012).

***6.* Association: STAT3 with Oxaliplatin**

Validation Status: Direct

Evidence: TF siRNA and Differential TF Concentration

LLR: 6.95

High Scoring Analyses: eQTL+M, eQTL

Observation: In a study exploring the immune response of oxaliplatin treatment, researchers showed that oxaliplatin decreased TLR-induced STAT1 and STAT3 expression in human T

cells via Western Blotting (Tel et al. 2012). In mouse models of metastatic colorectal cancer (HCT116), siRNA silencing of *STAT3* combined with oxaliplatin therapy reduced tumor size by 96%, better than either treatment separately (77% and 57% respectively) (Shahzad et al. 2011). This interaction between oxaliplatin and *STAT3* was articulated in another study of HCT116 cells, where in vitro treatment of oxaliplatin was accompanied with IL-6 mediated activation of STAT3 as well as phosphorylation of Raf kinase inhibitor protein (RKIP) (Cross-Knorr et al. 2013). Work by Hua et al. in SKOV3 ovarian cancer cell lines recapitulated this relationship in a different cell line, with the conclusion being that oxaliplatin treatment upregulated STAT3β (Sheng et al. 2013).

7. **Association: RELA with Temozolomide**

Validation Status: Direct

Evidence: TF siRNA and Differential Expression of TF mRNA Expression

LLR: 6.52

High Scoring Analyses: eQTL+M, eQTL

Observation: A previous study demonstrated that the DNA - methylating drug temozolomide (temozolomide) activates the positive modulator of NF - κB, AKT, in a mismatch repair (MMR) system dependent manner. Mismatch repair systems are either proficient in the repair of DNA double strands breaks (DSBs), or deficient, conferring chemosensitivity to the former and chemoresistance to the latter. A subsequent investigation into whether NF – κB is activated by temozolomide and whether AKT is involved in the molecular biology of this event revealed several interactions between the NF – κB family member, RELA, and temozolomide. Treatment of temozolomide to proficient MMR systems enhanced NF – κB transcriptional activity, activated AKT, and induced RELA nuclear translocation in only MMR-proficient cells. Upregulation of NF – κB transcription and RELA translocation were impaired

in KD12 cells treated with temozolomide and transfected with siRNA targeting AKT. Additionally, RELA silencing in deficient MMR systems increased temozolomide-induced growth suppression (Caporali et al. 2012). An examination of temozolomide inhibition of NF – κB activity revealed that O6-methylguanine inhibits RELA DNA binding; Another study showed differential *RELA* expression in response to temozolomide treatment in glioma cells (Yamini et al. 2007).

8 - 9. Association: HNF4G with Epirubicin and Doxorubicin

Validation Status: Direct

Evidence: TF siRNA

LLR: 6.20 and 5.48 respectively

High Scoring Analyses: eQTL+M and eQTL for both

Observation: Our previous work demonstrated the link between the anthracyclines, epirubicin and doxorubicin, with HNF4G by using siRNA knockdown of HNF4G in two triple negative breast cancer cell lines: MDA-MB-231 and BTF459. The cytotoxicity curve of cell population survivability versus the log concentration of epirubicin or doxorubicin treated was significantly altered in both MDA-MB-231 and BTF459 when transfected with *HNF4G* siRNA (Hanson et al. 2015) and compared to DMSO control experiments.

10. **Association: GATA1 and Rapamycin**

Validation Status: Indirect

Evidence: Differential TF DNA Binding

LLR: 5.78

High Scoring Analyses: eQTL+M, eQTM

Observation: Research on the involvement of phosphoinosite 3-kinases (PI3K) in cellular differentiation was investigated in the context of friend murine erythroleukaemia cells. The early hours of dimethyl sulfoxide (DMSO) or hexamethylenebisacetamide (HMBA) exposure to these cells commits them to a cessation of growth and differentiation. Treatment of these inducers to friend erythroleukaemia cells increased DNA binding of GATA1, an important transcription factor for erythroid specific genes. When treated with the S6-kinase inhibitor, rapamycin, HMBA cells induced at 18 hours showed markedly lower binding of GATA1 to the DNA (Bavelloni et al. 2000). Together, this indicated that rapamycin may inhibit binding of GATA1 to DNA via the PI3K dependent AKT/p70 S6-kinase pathway. Another study concluded that mammalian target of rapamycin (mTOR), which is related to the aforementioned AKT pathway, tightly regulates GATA1 protein expression at the post-transcriptional level (Liu et al. 2011).

11. **Association: BATF with Docetaxel**

Validation Status: Direct

Evidence: TF siRNA

LLR: 5.57

High Scoring Analyses: eQTL+M

In the study by Hanson et al., the effect of BATF on the cytotoxicity of paclitaxel and docetaxel on the triple negative breast cancer cell lines, BTF549 and MDA-MB-231. Silencing BATF shifted both the cytotoxicity curves of paclitaxel and docetaxel in MDA-MB-231 significantly with respect to DMSO controls, but not in BTF549 (Hanson et al. 2015).

***12.* Association: NANOG with Oxaliplatin**

Validation Status: Indirect

Evidence: Differential Expression of TF mRNA and Differential TF Protein Concentration

LLR: 5.46

High Scoring Analyses: eQTM only

Observation: Ex vivo models derived from human colorectal liver metastases were treated with oxaliplatin, 5-flurouracil, and curcumin. Compared to DMSO controls, at 72 hours of treatment, the triplicate treatment significantly downregulated expression of *NANOG* (James et al. 2015). NANOG protein concentration was also shown to be decreased in colorectal cancer stem cells (CRSCs) when treated with thiostrepton, which acts synergistically with oxaliplatin in killing CRSCs (Ju et al. 2015). Additionally, research has shown that NANOG amplifies *STAT3* activation, a regulator shown to be associated with oxaliplatin cellular response (Stuart et al. 2014) (Cross-Knorr et al. 2013). Finally, the Notch signaling pathway, which targets NANOG (Capaccione and Pine 2013), was shown to be an important mediator of CRSC self-renewal and proliferation (http://ecommons.luc.edu/luc_diss/87).

***13.* Association: *ZNF274* with Ara-C**

Validation Status: Indirect

Evidence: Differential Expression of TF mRNA

LLR: 5.00

High Scoring Analyses: eQTL+M, eQTL

Observation: While no literature evidence was found that corroborates this finding, evidence does exist for the association between gemcitabine, which, like Ara-C, is a deoxycytidine analog. The link between gemcitabine and ZNF274 was reported by eQTL+M analysis with

an LLR of 3.11. A gene expression profile of gemcitabine treatment to breast cancer cells revealed that gemcitabine upregulates *ZNF274* expression (Hernandez-Vargas et al. 2007).

14. **Association: EZH2 with Radiation**

Validation Status: Direct

Evidence: TF siRNA, Differential Expression of TF mRNA and Regulatory Information

LLR: 4.83

High Scoring Analyses: eQTL+M, eQTM

Observation: In a study of epigenetic aberration in radioresistant OML1-R cells, researchers identified hypermethylation of the *FHIT* promoter by H3K27me3 and low *FHIT* expression. Further analysis revealed *EZH2* overexpression in these cells. EZH2 is a known transcriptional silencer via H3K27me3 and so the investigators knocked down EZH2 via shRNA to assess its impact on *FHIT*. Knockdown of EZH2 increased *FHIT* expression, decreased H3K27me3 in the *FHIT* promoter by 2-fold, increased H3K4me3 in the *FHIT* promoter by 2-fold, and reduced *FHIT* promoter methylation by 10%. An inhibitor of EZH2 H3K27me3, GSK343, resulted in increased expression of *FHIT;* combination therapy with the DNA methyltransferase inhibitor (DNMT) 5-Aza demonstrated the greatest increase of *FHIT* expression among all epigenetic silencers (Lin et al. 2015). In a study of glioblastoma (GBM) – derived tumorigenic stem-like cells (GSCs), Kim et al. looked at the role that the catalytic subunit of Polycomb repressive complex 2, EZH2, and the MELK-FOXM1 complex play in radiosensitivity. It was shown that not only are both EZH2 and MELK co-expressed in GBM and upregulated following radiation treatment, but that MELK mediated EZH2 signaling is required for GSC resistance to radiation. They further show that this function is evolutionarily conserved in Caenorhabditis elegans (C. elegans). The researchers further detailed the mechanisms by which EZH2, MELK, and FOXM1 may be interacting. Luciferase

assays in overexpression and knockout experiments showed increased *EZH2* promoter activity in the presence of MELK; this finding was corroborated by flow cytometry experiments. The conclusion of these findings was that MELK transcriptionally regulated *EZH2*, at least in GBM spheres. Given the lack of a MELK DNA binding domain, the researchers searched for a cofactor that MELK teamed with to regulate the expression of *EZH2*. The paper concluded that EZH2 is a direct target of the MELK/FOXM1 transcriptional complex (Kim et al. 2015).

15. **Association: NANOG with Cisplatin**

Validation Status: Direct

Evidence: TF siRNA

LLR: 4.62

High Scoring Analyses: eQTL+M, eQTL

Observation: The role of NANOG in esophageal squamous cell carcinoma (ESCC) was investigated, particularly its interaction with cisplatin. ESCCs were confirmed to have high expression of *NANOG* via RT-PCR. Several other squamous cell lines have been characterized by *NANOG* expression, which was hypothesized to be the reason for these cell lines chemosensitivity to cisplatin. When NANOG was silenced via siRNA in two ESCC cell lines, the growth inhibitory effect of cisplatin was significantly enhanced (Du et al. 2012).

Supplemental Note S2: Here, we list and discuss the top (TF, Drug) associations predicted by eQTL+M with an LLR >= 3, decomposed by treatment, and ranked by LLR. For each treatment, only up to seven TFs were examined. In parenthesis is the fraction of pairs validated for each drug, where validation is either direct (knockdown or overexpression) or indirect (differential expression, differential binding, etc…). Observations are also provided for pairs where we observed some evidence, but not enough to be considered either direct or indirect validation.

For each (TF, Drug) association, we report several fields. The first is the “Validation Status” field, which indicates whether we consider this association to be direct validation (exhibited usually by TF knockdown) or indirect (exhibited usually by TF mRNA differential expression or binding in response to the treatment). The second field, “Evidence”, summarizes the evidence we found for the association. The next field, “LLR”, contains the pGENMi eQTL+M LLR for the association. The following “High Scoring Analyses” field contains the experiments that yielded a significant LLR; for eQTL and eQTM, the LLR threshold was 1.74. Since we only examine significant eQTL+M pairs in this analysis, every association should at least have eQTL+M in this field. The last field is “Observation” which is a paragraph describing literature evidence we found corroborating the (TF, Drug) association or at least providing some evidence as to its validity.

6-MP: (1/7)

1. *Association:* WRNIP1 with 6-MP

   Validation Status: Not Validated

   Evidence: None

   LLR: 6.68

   High Scoring Analyses: eQTL+M, eQTM

2. *Association:* NANOG with 6-MP

   Validation Status: Not Validated (Not included as “some” evidence in **Table 2**)

   Evidence: None

   LLR: 4.51

   High Scoring Analyses: eQTL+M, eQTL

   Observation: Though we failed to corroborate this finding in the literature, we have found NANOG to be associated with at least 4 other drugs. Knockdown of NANOG has been shown to increase sensitivity in cancer cells to cytotoxic agents like cisplatin (Du et al. 2012) and reduce malignancy potential (Kawamura et al. 2015). Overexpression of *NANOG* has also been shown to increase resistance to docetaxel (Jeter et al. 2011). Thus, while we failed to corroborate this association, NANOG appears to be important in cancer and drug resistance more broadly.

3. *Association: RCOR1* with 6-MP

   Validation Status: Indirect

   Evidence: Regulated by Drug Target

   LLR: 4.23

   High Scoring Analyses: eQTL+M

Observation: The nuclear receptor NR4A2 has been shown to be activated by 6-MP in CV1 and HEK293 cells (Ordentlich et al. 2003). In microglia cells and astrocytes, NRF4A2 recruited COREST (RCOR1) in clearing RELA via transcriptional repression (Saijo et al. 2009). Given that NRFA2 is a target of 6-MP and interacts with RCOR1 in transcriptional repression of RELA, it stands to reason that *RCOR1* is associated with 6-MP, albeit indirectly through *NRFA2*.

4. *Association:* FOSL2 with 6-MP

Validation Status: Not Validated

Evidence: None

LLR: 3.99

High Scoring Analyses: eQTL+M, eQTM

5. *Association:* FOXA1 with 6-MP

Validation Status: Not Validated

Evidence: None

LLR: 3.89

High Scoring Analyses: eQTL+M, eQTL

6. *Association:* TCF7L2 with 6-MP

Validation Status: Not Validated (Some Evidence)

Evidence: Interaction with Drug Target

LLR: 3.77

High Scoring Analyses: eQTL+M, eQTL

Observation: TCF7L2 is known to be involved in drug resistance (Nishimoto et al. 2014). In investigating the effect of *NR4A3* variant (coding SNP rs12686676) in insulin gene regulation and insulin secretion in β-cells, researchers found a gene-gene interaction between the NR4A3 allele and a variant of TCF7L2 (SNP rs7903146). The NR4A3 allele is known to increase insulin secretion, while the TCF7L2 allele decreases secretion. Haplotypes with wild type *TCF7L2* and variant *NRFA3* do not exhibit significant increase or decrease in insulin secretion compared to haplotypes that are wildtype for both genes. However, haplotypes with variant *TCF7L2* and variant *NR4A3* show much higher secretion of insulin compared to haplotypes with wildtype *NR4A3* and variant *TCF7L2*, indicating that the NR4A3 can restore insulin secretion in comprised systems with variant TCF7L2. It is widely known that the *NR4A* subgroup can be activated by 6-MP via the AF-1 domain (Ordelheide et al. 2013). Given the interaction between TCF7L2 and NR4A3 as well as NR4A3's activation by 6-MP, we conclude that there is some evidence that *TCF7L2* is associated with 6-MP, but not enough to warrant indirect or direct validation status. In a separate study examining the effect of azathioprine, a purine anti-metabolite akin to 6-MP, on carcinogenesis in mice found frameshift mutations in *TCF7L2* and seven other genes involved in carcinogenesis when at least one copy of the DNA repair protein MSH2 in the mice was absent (Chalastanis et al. 2010).

7. *Association:* SPI1 with 6-MP

   Validation Status: Not Validated

   Evidence: None

   LLR: 3.74

   High Scoring Analyses: eQTL+M, eQTM

6-TG: (0/4)

1. *Association:* SMARCC1 with 6-TG

   Validation Status: Not Validated

   Evidence: None

   LLR: 7.26

   High Scoring Analyses: eQTL+M, eQTM

2. *Association:* WRNIP1 with 6-TG

   Validation Status: Not Validated

   Evidence: None

   LLR: 7.20

   High Scoring Analyses: eQTL+M, eQTM

3. *Association:* NANOG with 6-TG

   Validation Status: Not Validated

   Evidence: None

   LLR: 6.81

   High Scoring Analyses: eQTL+M, eQTL

4. *Association:* ARID3A with 6-TG

   Validation Status: Not Validated

   Evidence: None

   LLR: 6.24

   High Scoring Analyses: eQTL+M, eQTM

Ara-C: (5/6)

1. *Association:* ZNF274 with Ara-C

   Validation Status: Indirect

   Evidence: Reported in **Supplemental Note S1**

   LLR: 5.00

   High Scoring Analyses: eQTL+M, eQTL

2. *Association:* RAD21 with Ara-C

   Validation Status: Direct

   Evidence: TF siRNA and Cancer Remission

   LLR: 3.59

   High Scoring Analyses: eQTL+M

   Observation: In our study, siRNA knockdown of RAD21 in Jurkat cell lines shifted the cytotoxicity curve of Cytarabine significantly compared to DMSO controls. Additionally, somatic mutations in *RAD21* have been identified as a key driver in acute myeloid leukemia (AML) development; treatment of cytarabine and indarubicin has been shown to achieve remission in patients with cohesin mutations in *STAG2, SMC3, and RAD21* (Thota et al. 2014).

3. *Association:* TRIM28 with Ara-C

   Validation Status: Indirect

   Evidence: Differential Phosphorylation of TF by Gemcitabine

   LLR: 3.44

   High Scoring Analyses: eQTL+M, eQTL

Observation: TRIM28 is an ataxia telangiectasia mutated (ATM) substrate activated by DNA double strand breaks. In exposing multiple myeloma cells with a combination of gemcitabine (a pyrimidine analog akin to cytarabine) and a purine analog, clofarabine, ATM kinase substrates such as histone 2AX, TRIM28, and p53 were phosphorylated (Valdez et al. 2013), indicating that the ATM pathway (including TRIM28) is activated by this combination. Another study demonstrated that depletion of ATM in HeLa and A549 cells sensitized them to gemcitabine therapy – further supporting the importance of this pathway in gemcitabine treatment (Karnitz et al. 2005).

4. *Association:* ZEB1 with Ara-C

   Validation Status: Direct

   Evidence: TF Knockdown

   LLR: 3.31

   High Scoring Analyses: eQTL+M, eQTL

   Observation: In a study investigating the role of ZEB1 in differential response to chemotherapy in mantle cell lymphoma (MCL), the investigators knocked down ZEB1 by introducing salinomycin to MCL cells; ZEB1 depends on WNT signaling for expression and salnomycin is a WNT blocker. After ZEB1 knockdown, the researchers noticed increased chemosensitivity of MCL cells to the cytotoxic effects of doxorubicin, cytarabine and gemcitabine (Sanchez-Tillo et al. 2014).

5. *Association: TCF12* with Ara-C

   Validation Status: Indirect

   Evidence: Differential Expression of TF mRNA

   LLR: 3.20

High Scoring Analyses: eQTL+M, eQTM

Observation: In a work detailing the effects of cytarabine on ectoderm and mesoderm development in human embryonic stem cells (hESCs), the investigators performed a differential gene expression assay on hESCs. They demonstrated that cytarabine upregulates *TCF12* expression in this cell type (Jagtap et al. 2011).

6. *Association:* UBTF with Ara-C

   Validation Status: Not Validated

   Evidence: None

   LLR: 3.03

   High Scoring Analyses: eQTL+M

Arsenic: (1/1)

1. *Association:* EZH2 with Arsenic

   Validation Status: Direct

   Evidence: Kinase siRNA and miRNA siRNA and Overexpression

   LLR: 3.62

   High Scoring Analyses: eQTL+M, eQTL

   Observation: An analysis of the involvement of JNK and STAT3 in AKT-mediated phosphorylation of EZH2 revealed a critical insight into the role of arsenic in bronchial epithelial cells. It was shown that treating bronchial epithelial cells (BEAS-2B) with arsenic induces phosphorylation of EZH2. This was demonstrated by first transfecting cells with siRNA targeting JNK1; such cells showed a loss of phosphorylation of STAT3, diminished AKT activity, and thus a loss of phosphorylation on serine 21 of EZH2. The researchers also illustrated that arsenic targets the AKT pathway by inducing *miRNA-21*, a miRNA regulated by STAT3. Silencing this miRNA in transfected arsenic-induced cells diminished phosphorylation of EZH2, while ectopic overexpression of miRNA-21 resulted in EZH2 phosphorylation (Chen et al. 2013a). With respect to cell cycle arrest generally, EZH2 has also been implicated as a significant player in SWI/SNF, a family of proteins that consume ATP to remodel nucleosomes, deficient cells; this was shown via inhibition of EZH2 in SMARCB1 (a member of the SWI/SNF family) deficient rhabdoid cells, which led to alterations of H3K27 trimethylation and cytotoxicity (Masliah-Planchon et al. 2015)

Carboplatin: (1/1)

1. *Association:* SP1 with Carboplatin

   Validation Status: Direct

   Evidence: ChIP and TF Overexpression

   LLR: 3.85

   High Scoring Analyses: eQTL+M, eQTL

   Observation: It has been established via chromatin immunoprecipitation (ChIP) assays that, in tumor copper deficient cells, SP1 regulates SLC31A1 by binding to the *SLC31A1* promoter. The SLC31A1 protein is a membrane protein that transports copper into the cell, and, more importantly, platinum drugs such as cisplatin and carboplatin. Low expressing individuals of SLC31A1 are typically resistant to the chemotherapy of platinum drugs. It has been shown that SLC31A1 can be induced in copper deficient environments through upregulation of SP1, thereby leading to greater chemosensitivity to platinum agents. Such experiments have already been performed with cisplatin and preliminary studies are encouraging for carboplatin therapy (Chen and Kuo 2013). Additionally, a separate study showed that inhibition of SP1 binding to the promoter of *BIRC5* (survivin), an antiapoptotic protein highly expressed in cancer and linked to drug resistance, decreased expression of *BIRC5* (Chun et al. 2007), implicating SP1 more broadly in chemotherapy.

Cisplatin: (7/7)

1. *Association:* NANOG with Cisplatin

   Validation Status: Direct

   Evidence: Reported in **Supplemental Note S1**

   LLR: 4.62

   High Scoring Analyses: eQTL+M, eQTL

2. *Association:* RELA with Cisplatin

   Validation Status: Direct

   Evidence: TF Reporter Assay and TF siRNA

   LLR: 3.88

   High Scoring Analyses: eQTL+M, eQTM

   Observation: It was shown in HeLa 57A cells that treatment of cisplatin reduced transcriptional expression of *RELA*, via luciferase reporter assays. Additionally, further investigation found that cisplatin treatment downregulated *BCL2L1*. A separate study by the same group demonstrated that RELA is required for activation of *BCL2L1* through siRNA knockdown of RELA (Campbell et al. 2006). Another study examining the interaction of *RELA* and cisplatin focused on the differential phosphorylation of the T505 residue of RELA by CHEK1 in response to cisplatin treatment in mouse embryonic fibroblasts. The researchers found that T505 phosphorylation induced a proapoptotic form of RELA that could facilitate cell death through transcriptional repression of antiapoptotic target genes such as *BCL2L1* (Msaki et al. 2011).

3. *Association:* SMARCC1 with Cisplatin

   Validation Status: Direct

   Evidence: Indirect shRNA and Homolog deletion

   LLR: 3.53

   High Scoring Analyses: eQTL+M, eQTL

   Observation: SMARCC1 is a member of the SWI/SNF family of proteins that remodel nucleosomes. A study of the cytotoxicity of cisplatin in head and neck squamous cell carcinoma revealed that SMARCA4 and SMARCA1 increase sensitivity to cisplatin, via shRNA knockdown experiments (Kothandapani et al. 2012). Another study showed that the suppression of SWI/SNF factors (SMARCB1, SMARCC1, SMARCC2, SMARCD1, SMARCD3) via siRNA sensitized cells to cisplatin at the same level as ARID1A and ARID1B suppression in U2OS cells (Watanabe et al. 2014). Furthermore, in yeast, the *YJL175W* open reading frame deletion that confers cisplatin resistance overlaps with *SWI3*, the yeast ortholog of *SMARCC1* (Rabik and Dolan 2007).

4. *Association:* RUNX3 with Cisplatin

   Validation Status: Direct

   Evidence: TF siRNA and Differential Expression of TF mRNA

   LLR: 3.51

   High Scoring Analyses: eQTL+M, eQTL

   Observation: Examining the role of RUNX3 in gastric cancer chemotherapy, siRNA knockdown of RUNX3 sensitized immortalized stomach mucosal cells (GES-1) and gastric cancer cells (SGC7901) to cisplatin (Guo et al. 2005). Another study in hepatocellular carcinoma (HCC) looking at the role of miR-130a/RUNX3/WNT signaling in cisplatin treatment demonstrated through siRNA knockdown of *miR-130a* and RUNX3 that *miR-130a* inhibits

RUNX3 which activates WNT signaling and results in cellular resistance to cisplatin treatment (Xu et al. 2012). Further studies have shown differential expression of *RUNX3* in cells treated with cisplatin (Biswal et al. 2012) (Dadarkar et al. 2010).

5. *Association:* WRNIP1 with Cisplatin

   Validation Status: Indirect

   Evidence: Cooperators siRNA

   LLR: 3.49

   High Scoring Analyses: eQTL+M, eQTM

   Observation: In an investigation of ZRANB3, siRNA knockout in U2OS cells resulted in dramatic sensitization to camptothecin (CPT) and moderate sensitization to cisplatin. The study also elucidated cooperativity between ZRNAB3 and WRNIP1 (Ciccia et al. 2012). In a study of chicken DT40 cells, knockdowns of WRNIP1 did not desensitize the cell to cisplatin, but moderately to CPT; however, negative cells of RAD18, which interacts with WRNIP1, did exhibit high sensitivity to both cisplatin and CPT (Yoshimura et al. 2006).

6. *Association: NR3C1* with Cisplatin

   Validation Status: Indirect

   Evidence: Differential Expression of TF mRNA

   LLR: 3.10

   High Scoring Analyses: eQTL+M,eQTL

   Observation: In OC-k3 mouse hairs cells, researchers showed that cisplatin treatment upregulated expression of *NR3C1* in vitro (Low et al. 2010). However, in a transcriptomic profile of human cellular response to cytotoxic agents, *NR3C1* was found to be downregulated by cisplatin treatment (Limonciel et al. 2015). This contradiction may be due to the differing

regulatory architectures between the species, as it is likely cisplatin does not interact directly with NR3C1 Additionally, NR3C1 is a glucocorticoid receptor (GR) and glucocorticoids (GC) have been shown to be enhance cytotoxicity (Lu et al. 2006).

7. *Association:* CEBPD with Cisplatin

   Validation Status: Direct

   Evidence: TF Transfection and Differential Expression of TF mRNA and Concentration of TF

   LLR: 3.07

   High Scoring Analyses: None

   Observation: In analysis of human urothelial carcinoma cell line NTUB1, researchers examined the following cell sublines resistant to particular drugs to analyze the differences in CEBPD expression: cisplatin (NTUB1/P(14)), gemcitabine (NTUB1/G(1.5)), arsenic trioxide (NTUB1/As(0.5)), and paclitaxel (NTUB1/T(0.017)). CEBPD was only expressed in the cisplatin resistant cell subline, NTUB1/P(14), and in none of the parental cell lines. When treating NTUB1 cells with cisplatin, CEBPD protein and mRNA expression levels were unilaterally elevated across the cell lines. In determining its role in chemoresistance, CEBPD was overexpressed in NTUB1 cells treated with increasing amounts of cisplatin. Compared to controls, CEBPD overexpressed cells were significantly more resistant to cisplatin-induced apoptosis. The paper further articulates that CEBPD reduces cisplatin-induced reactive oxygen species (ROS) production by inducing the expression of Cu/Zn-superoxide dismutase (SOD1) (Hour et al. 2010). In a study of ototoxicity in mouse cells, *CEBPD* expression was also affected by administration of cisplatin (Low et al. 2010). Another study in human found that cisplatin upregulated *CEBPD* (Tardito et al. 2009), which is consistent with Hour et al.

Docetaxel: (1/1)

1. *Association:* BATF with Docetaxel

   Validation Status: Direct

   Evidence: Reported in **Supplemental Note S1**

   LLR: 5.57

   High Scoring Analyses: eQTL+M

Doxorubicin: (3/3)

1. *Association:* HNF4G with Doxorubicin

   Validation Status: Direct

   Evidence: Reported in **Supplemental Note S1**

   LLR: 5.48

   High Scoring Analyses: eQTL+M, eQTL

2. *Association:* HMGN3 with Doxorubicin

   Validation Status: Direct

   Evidence: TF siRNA

   LLR: 3.55

   High Scoring Analyses: eQTL+M, eQTL

   Observations: In the paper by Hanson et al., HMGN3 was silenced via siRNA in the negative breast cancer cell line, MDA-MB-231. The knocked down cell lines were treated with either doxorubicin or epirubicin. Compared to control, HMGN3 knockdowns were more resistant to anthracycline-induced apoptosis in MDA-MB-231 cells, but not BTF459 (Hanson et al. 2015). In a study profiling gene expression in 30 different cell lines treated with 11 different drugs, *HMGN3* expression level was predictive of doxorubicin sensitivity across cells (Gyorffy et al. 2006).

3. *Association: TCF7L2* with Doxorubicin

   Validation Status: Indirect

   Evidence: Indirect Pathway Evidence and Differential Concentration of TF

   LLR: 3.50

   High Scoring Analyses: eQTL+M, eQTL

Observations: TCF7L2 is a member of the WNT/β-Catenin pathway. In a recent paper by Vangipuram et al., the activity of this pathway was investigated in the context of chemoresistance in cancer stem-like cells in a neuroblastoma cell line. To start, the investigators segmented the SK-N-SH into resistant (CD133+) and sensitive groups (CD133-) to doxorubicin treatment. Pathway activity scoring showed suppression of the WNT pathway in doxorubicin treated CD133- cells. WNT pathway genes were more differentially expressed in CD133+ cells compared to CD133- cells. When treated with WNT agonists, doxorubicin was very effective in reducing the population of CD133+ cells. This indicates that doxorubicin efficacy is related to the pathway activity of WNT/β-Catenin (Vangipuram et al. 2012); however, this doesn't necessarily link the drug directly to *TCF7L2*. A study in the mouse intestinal track demonstrated that doxorubicin affects the expression TCF7L2 (de Koning et al. 2007).

Epirubicin: (3/4)

1. *Association:* HNF4G with Epirubicin

   Validation Status: Direct

   Evidence: Reported in **Supplemental Note S1**

   LLR: 5.48

   High Scoring Analyses: eQTL+M, eQTL

2. *Association:* TCF7L2 with Epirubicin

   Validation Status: Not Validated

   Evidence: None Found

   LLR: 3.50

   High Scoring Analyses: eQTL+M, eQTL

3. *Association:* HMGN3 with Epirubicin

   Validation Status: Direct

   Evidence: Reported in **Doxorubicin Section**

   LLR: 3.55

   High Scoring Analyses: eQTL+M, eQTL

4. *Association:* TAL1 with Epirubicin

   Validation Status: Direct

   Evidence: TF Overexpression

   LLR: 3.2.1

   High Scoring Analyses: eQTL+M, eQTL

Observation: TAL1 is expressed frequently in human T-cell acute lymphoblastic leukemia. However, there are aberrations in the expression of TAL1 among leukemia cells. In investigating the cytotoxic effect of TAL1, Bernard et al. transfected human immature T-cell lymphoid cell lines with TAL1. After treating with various cytotoxic agents including doxorubicin, the researchers concluded that the transfectants were more resistant to the cytotoxic effects of these drugs. TAL1 transfectants lacking a DNA binding domain did not show altered sensitivity, revealing that TAL1 binding to DNA is important for this to occur. The researchers, therefore, concluded that TAL1 acts at a late stage of the apoptotic cascade (Bernard et al. 1998). While doxorubicin is not epirubicin, the two are very similar in structure, belonging to the same drug class (anthracyclines). Additionally, the eQTL+M (TAL1, Doxorubicin) association yielded an LLR score of 2.81, which barely missed our threshold of 3. Therefore, we feel confident generalizing TAL1's association with doxorubicin to epirubicin.

Gemcitabine: (3/5)

1. *Association:* ZNF143 with Gemcitabine

   Validation Status: Not Validated (Some Evidence)

   Evidence: Differential Expression of TF mRNA and TF siRNA (with Cisplatin)

   LLR: 3.37

   High Scoring Analyses: eQTL+M, eQTL

   Observations: In looking at respiratory deficient mitochondrial cells, it was shown that cell lines with respiratory dysfunction were more resistant to death via gemcitabine treatment than their normal counterparts. It was also shown that such cells had higher *ZNF143* mRNA levels compared to normal respiratory cells. While gemcitabine treatment with ZNF143 knockdown was not reported, dysfunctional cells showed greater sensitivity to cisplatin after ZNF143 was knocked down (Lu et al. 2012). Thus, some evidence exists that ZNF143 can elicit chemoresistance under certain circumstances.

2. *Association:* USF1 with Gemcitabine

   Validation Status: Not Validated (Some Evidence)

   Evidence: Differential Expression of Drug Target and Regulatory Information

   LLR: 3.24

   High Scoring Analyses: eQTL+M

   Observation: Both gemcitabine and ara-C rely on deoxycytidine kinase (DCK) in the first rate limiting steps of the activation of these nucleoside agents in solid tumors and leukemia, respectively (Ge et al. 2005). In vivo ChIP assays of HepG2 cells showed the presence of USF1/2 and SP1/2 bound factors to the *DCK* promoter. Co-transfections in HepG2 showed activation properties of USF1/2 binding and repressive properties of SP1 binding to a *DCK*-luciferase reporter construct (Ge et al. 2003). A separate study showed that *DCK* expression

was predictive of ara-C IC50 in acute myeloid leukemia (AML) cell lines; among the aforementioned regulators of *DCK*, only *USF1* expression was variable and correlated with *DCK* (Ge et al. 2005). Given that USF1 has been shown to regulate a gene important in the pharmacokinetics of both gemcitabine and cytarabine and is predictive of cytarabine IC50 in AML cell lines, we conclude that there is some evidence to suggest USF1 is associated with gemcitabine.

3. *Association: ZNF274* with Gemcitabine

   Validation Status: Indirect

   Evidence: Differential Expression of TF mRNA

   LLR: 3.11

   High Scoring Analyses: eQTL+M, eQTM

   Observation: A gene expression profiling of breast cancer cell lines in response to gemcitabine treatment revealed numerous differentially expressed genes; *ZNF274* was upregulated in response to gemcitabine treatment (Hernandez-Vargas et al. 2007).

4. *Association:* ARID3A with Gemcitabine

   Validation Status: Not Validated

   Evidence: None Found

   LLR: 3.06

   High Scoring Analyses: eQTL+M, eQTL

5. *Association:* MAZ with Gemcitabine

   Validation Status: Not Validated

   Evidence: None Found

LLR: 3.03

High Scoring Analyses: eQTL+M, eQTL

Hypoxia: (1/1)

1. *Association:* PRDM1 with Hypoxia

   Validation Status: Direct

   Evidence: Regulator of Treatment Target and Differential Expression of TF mRNA

   LLR: 3.96

   High Scoring Analyses: eQTL+M, eQTL

   Observation: PRDM1 is one of two major transcription factors critical for *XBP1* expression; it does so by repressing *PAX5*, itself a repressor of *XBP1,* thereby enhancing *XBP1* expression (He et al. 2010). As it turns out, XBP1 is essential for hypoxia survival and is required for tumor growth (Romero-Ramirez et al. 2004). Though not TF siRNA evidence, we consider regulation of a gene essential for treatment survival to be direct evidence of PRDM1's association with Hypoxia. In multiple myeloma (MM) cells, researchers found that hypoxia induces the downregulation of plasma specific TFs and upregulated stem-cell associated TFs. Among those TFs downregulated in hypoxic MM cells compared to normoxic MM cells was *PRMD1* (Kawano et al. 2013). Paradoxically, transcriptomic profiling in other cells in hypoxic and normoxic conditions revealed upregulation of *PRDM1* mRNA in response to oxygen deprivation (Limonciel et al. 2015) (Fiedler et al. 2015). This suggests PRDM1 is affected differently depending on the pathways induced by hypoxia.

NAPQI: (4/7)

1. *Association:* MEF2C with NAPQI

   Validation Status: Not Validated (Some Evidence)

   Evidence: Reported in **Supplemental Note S1**

   LLR: 7.17

   High Scoring Analyses: eQTL+M, eQTL, eQTM

2. *Association: NFIC* with NAPQI

   Validation Status: Indirect

   Evidence: Reported in **Supplemental Note S1**

   LLR: 7.08

   High Scoring Analyses: eQTL+M, eQTL

3. *Association:* CTCFL with NAPQI

   Validation Status: Not Validated

   Evidence: None Found

   LLR: 6.90

   High Scoring Analyses: eQTL+M, eQTL

4. *Association: CCNT2* with NAPQI

   Validation Status: Indirect

   Evidence: Reported in **Supplemental Note S1**

   LLR: 5.67

   High Scoring Analyses: eQTL+M, eQTM

5. *Association: UBTF* with NAPQI

   Validation Status: Indirect

   Evidence: Reported in **Supplemental Note S1**

   LLR: 4.90

   High Scoring Analyses: eQTL+M, eQTL

6. *Association:* CBX3 with NAPQI

   Validation Status: Not Validated

   Evidence: None Found

   LLR: 4.37

   High Scoring Analyses: eQTL+M, eQTM

7. *Association:* BCLAF1 with NAPQI

   Validation Status: Indirect

   Evidence: Differential Concentration of TF

   LLR: 4.31

   High Scoring Analyses: eQTL+M, eQTL

   Observation: In a gene expression analysis of precision cut human liver slices, treatment of APAP-induced upregulation of BCLAF1 (Elferink et al. 2011). BCLAF1 has also been shown to promote cell death generally (Kasof et al. 1999).

Oxaliplatin: (5/7)

1. *Association:* STAT3 with Oxaliplatin

   Validation Status: Direct

   Evidence: Reported in **Supplemental Note S1**

   LLR: 6.95

   High Scoring Analyses: eQTL+M, eQTL

2. *Association: NANOG* with Oxaliplatin

   Validation Status: Indirect

   Evidence: Reported in **Supplemental Note S1**

   LLR: 5.46

   High Scoring Analyses: eQTL+M, eQTL

3. *Association:* TRIM28 with Oxaliplatin

   Validation Status: Indirect

   Evidence: Differential Phosphorylation of TF and Differential Concentration of TF

   LLR: 3.58

   High Scoring Analyses: eQTL+M, eQTM

   Observation: While there exists little direct evidence linking TRIM28 to oxaliplatin, studies have shown that cisplatin increases phosphorylation (Pines et al. 2011) (Hendriks et al. 2012) and mRNA production (when used in concert with piroxicam) (Baldi et al. 2011) of TRIM28. One prominent study on three non-small cell lung cancer (NSLC) cell lines transformed into tumor-initiating cells (TICs) via stem cell media found impaired phosphorylation of TRIM28 due to irradiation and cisplatin treatment; the researchers hypothesized that the inhibition of TRIM28 phosphorylation might provide a mechanistic explanation for the observed reduction

in DNA damage-induced cell cycle arrest and apoptosis in NSCLC TICs (Lundholm et al. 2013). This speaks more broadly to the phosphorylation of TRIM28 desensitizing DNA damaged cells to death rather than to its association with a specific drug. Another very relevant study elucidating the connection between ataxia telangiectasia and Rad3-related (ATR) protein inhibitors and cisplatin demonstrated increased phosphorylation due to mediation by ATR; the same cells sensitized by the ATR inhibitor combined with cisplatin were also sensitized by the ATR inhibitor and oxaliplatin, though to a lesser degree (Hall et al. 2014). However, S-phase cell arrest via ATR appeared to be sensitive to cisplatin, and not oxaliplatin (Lewis et al. 2009), limiting the generalizability of the association of discussion to oxaliplatin. Mechanistically, though, it is still plausible for there to be an association. TRIM28 promotes re-sectioning of DNA double strand breaks not protected by γ-H2AX (Tubbs et al. 2014) in murine G1-phase lymphocytes and oxaliplatin has been shown to induce γ-H2AX (Chiu et al. 2009). In terms of chemotherapeutic drugs more generally, a study found that SKOV3 cells overexpressed with TRIM28 showed increased resistance to cisplatin and paclitaxel (Hu et al. 2015), despite the notable differences in mechanism of action between platinum agents and taxane therapies. Thus, while there is sufficient evidence between a TRIM28 and cisplatin association, there are mechanistic reasons to believe it can be generalized to oxaliplatin, a drug of the same family as cisplatin. As for why TRIM28 was not reported as an association with cisplatin, the best score (2.44) didn't make the cutoff threshold of 3 in the eQTL+M analysis.

4. *Association: FOSL1* with Oxaliplatin

   Validation Status: Indirect

   Evidence: Differential Expression of TF mRNA and Network Analysis

   LLR: 3.47

High Scoring Analyses: eQTL+M, eQTL

Observation: An experiment involving the co-treatment of oxaliplatin with topotecan in bone marrow from rats showed evidence of upregulation of *FOSL1* when treated with this cocktail (Davis et al. 2015). In addition, FOS signaling has been shown to be activated by oxaliplatin treatment in a variety of cancers (Alian et al. 2012). In our analysis, *FOS* association with oxaliplatin was also noteworthy with a score of 3.61, but it was not in the top 7 associated TFs with oxaliplatin.

5. *Association: ZEB1* with Oxaliplatin

   Validation Status: Indirect

   Evidence: Differential Expression of TF mRNA and Regulates Drug Regulator

   LLR: 3.26

   High Scoring Analyses: eQTL+M, eQTM

   Observation: In colon cancer cell line THC8307/L-OHP, oxaliplatin treatment downregulated *ZEB1* expression (Tang et al. 2007). ZEB1 has also been shown to increase tumorigenicity by repressing stemness-inhibiting miRNAs like *miR-203* (Wellner et al. 2009), which has been shown to increase oxaliplatin resistance in colorectal cancer cells (Zhou et al. 2014).

6. *Association:* ATF1 with Oxaliplatin

   Validation Status: Not Validated

   Evidence: None

   LLR: 3.10

   High Scoring Analyses: eQTL+M, eQTM

7. *Association:* PPARGC1A with Oxaliplatin

   Validation Status: Direct

   Evidence: TF siRNA

   LLR: 3.05

   High Scoring Analyses: eQTL+M, eQTM

   Observation: PPARGC1A siRNA knockdowns were performed in colon cancer liver metastases treated with the chemotherapeutic agents oxaliplatin and 5-fluorouracil (5-FU); knockdown of PPARGC1A prevented chemotherapy-induced oxidative phosphorylation (OXPHOS). The study concluded that colorectal tumors shift energy production from glycolysis to OXPHOS via the SIRT1/PPARGC1A pathway (Vellinga et al. 2015).

Paclitaxel: (2/3)

1. *Association:* CTBP2 with Paclitaxel

   Validation Status: Not Validated

   Evidence: None

   LLR: 4.44

   High Scoring Analyses: eQTL+M, eQTM

2. *Association: GATA1* with Paclitaxel

   Validation Status: Indirect

   Evidence: Genomic Alteration and Differential Concentration of TF

   LLR: 4.81

   High Scoring Analyses: eQTL+M, eQTL

   Observation: Chronic myelogenous leukemia (CML) resulting from a t(9,22) translocation is resistant to paclitaxel treatment. Researchers found that this produced an oncoprotein that activated a GATA1 response element in the promoter of a heat shock protein, HSP70. The siRNA knockdown of HSP7 0sensitized the cell to paclitaxel (Ray et al. 2004). Additionally, a murine study of the timeline of paclitaxel-induced cellular changes exhibited upregulation of GATA1 expression (Aguirre et al. 2010).

3. *Association:* E2F1 with Paclitaxel

   Validation Status: Direct

   Evidence: Differential Concentration of TF and TF Over Expression

   LLR: 3.58

   High Scoring Analyses: eQTL+M, eQTM

Observation: Low dose application of paclitaxel human retinoblastoma cells exhibited upregulation of E2F1 (Drago-Ferrante et al. 2008). Additionally, E2F1 overexpression in human osteosarcoma U2OS cells sensitized the cells to paclitaxel (Russo et al. 2006).

Radiation: (4/4)

1. *Association:* EZH2 with Radiation

   Validation Status: Direct

   Evidence: Reported in **Supplemental Note S1**

   LLR: 4.83

   High Scoring Analyses: eQTL+M, eQTM

2. *Association:* ESR1 with Radiation

   Validation Status: Direct

   Evidence: TF Activation and Differential Expression of TF mRNA

   LLR: 4.36

   High Scoring Analyses: eQTL+M, eQTL

   Observation: In a study examining the molecular mechanisms underlying the transformation of immortalized cells into tumorigenic cells, researchers found that *ESR1*, while low expressed, was differentially expressed between immortalized mammary epithelial cells and those induced into tumorigenesis via heavy-ion radiation (Ma et al. 2012). A study examining the bystander effect, where cells respond to their neighbors, of irradiation in variable estrogen receptor (ER) environments provides clearer proof of this association. The researchers irradiated MDA-MB-231 cells, which are ER negative, and MCF-7 cells, which are ER positive; additionally, they treated both cells with 17β-estradiol (E2), which activates ESR1, and tamoxifen, which is an E2 antagonist. MCF-7 cells, which have ESR1, exhibited increased radiosensitivity and bystander response when treated with E2; the effect was diminished by tamoxifen. E2 also increased MCF-7 reactive oxygen species (ROS), absent radiation; however, in MDA-MB-231, neither the bystander response nor ROS increase was observed

(Shao et al. 2008). Given that ESR1 activation sensitized MCF-7 cells to radiation, we consider this direct validation.

3. *Association:* PML with Radiation

   Validation Status: Direct

   Evidence: Immunofluorescence, TF siRNA, TF Over Expression, Northern Blotting

   LLR: 3.19

   High Scoring Analyses: eQTL+M

   Observation: Mouse embryo fibroblasts (MEFs) proficient and deficient in PML were irradiated to determine the effect of PML in radiation-induced apoptosis. PML deficient cells were much more resistant to radiation, indicating that PML mediates the apoptosis of radiation therapy. Overexpression of PML upon cellular irradiation potentiated c-Jun transcriptional activation and co-activation of c-Jun by PML was observed exclusively in irradiated cells. ChIP experiments of irradiated and unaffected cells showed that binding of c-Jun to its promoter was observed in irradiated, but not unaffected cells. Super shift analysis also showed that the DNA binding ability of c-Jun/ATF-2 was comprised in PML deficient irradiated cells (Salomoni et al. 2005). A separate examination of the interaction between PML and TOPBP1 demonstrated that both co-localize in the nucleus of the cervical cell lines, SiHa, to repair DNA damage after irradiation. Additionally, siRNA PML knockouts exhibited a decrease in radiation-induced TOPBP1 expression, suggesting PML is a regulator of TOPBP1. However, overexpression of PML did not increase mRNA levels of *TOPBP1*, but did increase TOPBP1 protein expression. Furthermore, pulse-chase labeling experiments indicated that PML increases the half-life of TOPBP1 protein, indicating that this regulation occurs at the post-transcriptional level (Xu et al. 2003). A separate analysis in HeLa cells treated by radiation and cisplatin showed upregulation of PML protein in response to treatment, although

northern blotting did not indicate a gross increase in mRNA levels. Transfection of p53 into HeLa upregulation of *PML* with respect to control, indicating that PML is regulated by the p53 pathway (Chan et al. 1997).

4. *Association:* HDAC2 with Radiation

   Validation Status: Direct

   Evidence: Differential Concentration of TF and TF siRNA

   LLR: 3.02

   High Scoring Analyses: eQTL+M, eQTM

   Observation: In non-small cell lung cancer (nsCL) BE1 cells, HDAC1 and HDAC2 expression were highly correlated and significantly higher than normal tissues. Prognosis of patients with low expression of these HDACs was noticeably higher compared to high expression patients. Irradiation of BE1 cells downregulated HDAC1 and HDAC2 expression and upregulated *AXIN* expression. Knockdowns of HDAC1 and HDAC2 via siRNA upregulated AXIN expression as well. Radiation treatment combined with HDAC knockdown in BE1 cells expressing AXIN increased apoptosis of cells compared to radiation treatment alone (Han et al. 2012). In fact, there is a growing body of literature that suggest combination therapies of HDAC inhibitors with radiation in chemotherapy treatment (New et al. 2012).

Rapamycin: (3/3)

1. *Association:* GATA1 with Rapamycin

   Validation Status: Direct

   Evidence: TF ChIP and TF siRNA

   LLR: 5.78

   High Scoring Analyses: eQTL+M, eQTM

   Observation: There is a close relationship between the mTOR pathway, which rapamycin directly targets, and the PI3K share a lot of crosstalk, leading to the development of dual inhibitors (Zaytseva et al. 2012). In paper examining PI3K inhibition in regards to differentiation in erythroleukaemia cells, the researchers noticed that treatment with the mTOR inhibitor rapamycin dramatically reduced GATA1 binding to DNA, which is essential in erythroid differentiation (Bavelloni et al. 2000). Another investigation into the role of epidermal growth factor (EGF) on upregulation of the excision-repair cross-complementary 1 (ERCC1) gene in human hepatocarcinoma cells (HuH7) revealed that in EGF-induced HuH7 cells, PI3K inhibition combined with silencing of the PI3K pathway kinase FKBP12-rapamycin-associated protein or mammalian target of rapamycin (FRAP/mTOR) upregulated ERCC1. Additionally, motif search identified a binding site for GATA1 in the promoter of *ERCC1* with ChIP confirming the binding of GATA1 to the promoter in EGF-induced cells (Andrieux et al. 2007). This indicates that GATA1 plays a role in mTOR/PI3K signaling, as it is a possible regulator of a target of such a pathway. This association was also corroborated by our own siRNA knockdown of GATA1 in MDA-MB-231 cells treated with rapamycin; cells were more resistant to rapamycin-induced apoptosis when GATA1 was knocked down.

2. *Association:* STAT2 with Rapamycin

   Validation Status: Direct

   Evidence: Western Blotting, Fluorescence, and TF siRNA

   LLR: 3.20

   High Scoring Analyses: eQTL+M

   Observation: An experiment treating lung adenocarcinoma cells (A549) with rapamycin identified STAT1 interacting with mTOR and increased STAT1 nucelar concentration after rapamycin treatment (Fielhaber et al. 2009). While STAT1 is not STAT2, the two form heterodimers and are members of the same protein family. Additionally, the pGENMI *STAT1* association with Rapamycin score was 2.49, which though below threshold, is very similar to the *STAT2* score. We believe that STAT2 is interpreting the same signal in this context and thus the association is valid. This association was also validated in our own experimental validations of the effect of STAT2 knockdown on rapamycin treated MDA-MB-231 cells.

3. *Association:* PHF8 with Rapamycin

   Validation Status: Direct

   Evidence: TF Over Expression and TF siRNA

   LLR: 3.08

   High Scoring Analyses: eQTL+M

   Observation: A study showed that PHF8 mRNA and protein levels were downregulated in failing human and mice hearts undergoing hypertrophy. Overexpression of PHF8 identified the aKT/mTOR pathway as a target of PHF8. When this pathway was inhibited by treatment of rapamycin, the phenotype lost by PHF8 deficiency was rescued (Liu et al. 2015). As in the previous two associations with rapamycin, we corroborated this association in the laboratory.

Triciribine: (2/2)

1. *Association:* REST with Triciribine

   Validation Status: Indirect

   Evidence: Regulation of Drug Target

   LLR: 4.14

   High Scoring Analyses: eQTL+M, eQTM

   Observation: A study of the regulatory dynamics of REST in small cell lung cancer revealed a putative binding site for REST at the 3' end of the *AKT2* UTR. Previous studies had shown that siRNA mediated knockdown of REST correlated with increased AKT phosphorylation. In this particular study, siRNA knockdown of REST resulted in upregulation of AKT2. Temporal gene expression profiling also showed high *AKT2* expression in small cell lung cancer cell lines only days after high expression of REST diminished (Kreisler et al. 2010). Though we lack direct evidence, given REST's regulation of AKT2 and triciribine's role as an AKT-inhibitor, we are confident in confirming this association at least partially.

2. *Association:* NANOG with Triciribine

   Validation Status: Indirect

   Evidence: Regulated by Drug Target

   LLR: 3.60

   High Scoring Analyses: eQTL+M, eQTL

   Observation: While we lack direct evidence of NANOG's association with triciribine, there is plenty of data linking NANOG with AKT. For instance, a study showed that the PI3K/AKT pathways is important in mediating the regulation of NANOG during differentiation of embryonic carcinoma F9 cells (Kim et al. 2010). Another study elucidated that AKT-mediated phosphorylation is crucial for repression of NANOG in differentiating murine embryonal

carcinoma cells (Chen et al. 2013b). Taken together, we believe there exists a plausible connection between NANOG and the AKT inhibitor triciribine.

Temozolomide: (3/7)

1. *Association:* FOXM1 with Temozolomide

   Validation Status: Direct

   Evidence: Reported in **Supplemental Note S1**

   LLR: 7.40

   High Scoring Analyses: eQTL+M, eQTL, eQTM

2. *Association:* RELA with Temozolomide

   Validation Status: Direct

   Evidence: Reported in **Supplemental Note S1**

   LLR: 6.51

   High Scoring Analyses: eQTL+M, eQTL

3. *Association:* USF1 with Temozolomide

   Validation Status: Not Validated

   Evidence: None Found

   LLR: 4.69

   High Scoring Analyses: eQTL+M, eQTL

4. *Association:* EBF1 with Temozolomide

   Validation Status: Not Validated (Some Evidence)

   Evidence: None Found

   LLR: 4.49

   High Scoring Analyses: eQTL+M, eQTL

Observation: In the treatment of glioblastoma, temozolomide works by operating as a methyl donor for the alkylation of the N-7, O-3, and O-6 positions of nucleotide bases, initiating a DNA repair process that cannot undo this level of damage. O-6-methylguanine methyltransferase (MGMT) removes methyl groups from the O-6 position of guanines, thereby rendering temozolomide ineffective (Weisenberger 2014). IDH1 mutations have been shown to predict longer survival times via treatment of temozolomide (Houillier et al. 2010). It has also been shown that EBF1 can bind to both DNA and TET2 and function as a demethylation agent in IDH1 mutants (Guilhamon et al. 2013). Thus, EBF1 may have a role to play in differential methylation of the MGMT promoter in IDH1 mutants, and thus have a role to play in cellular response to temozolomide.

5. *Association:* ELF1 with Temozolomide

   Validation Status: Direct

   Evidence: TF siRNA

   LLR: 4.46

   High Scoring Analyses: eQTL+M, eQTM

   Observation: While we did not find any literature evidence for this association, we validated it through TF siRNA in U251 glioma cells.

6. *Association:* RUNX3 with Temozolomide

   Validation Status: Not Validated

   Evidence: None Found

   LLR: 4.35

   High Scoring Analyses: eQTL+M, eQTL

7. *Association:* SMARCC2 with Temozolomide

   Validation Status: Not Validated

   Evidence: None Found

   LLR: 4.23

   High Scoring Analyses: eQTL+M, eQTM

Supplemental Note S3: Supplemental Results and Discussion

pGENMi produces distinct associations than simple baseline methods

The following results extend the section of the same name in the main text. We investigated the extent to which sub-significant results in the baseline were enriched with pGENMi. Relaxing the 0.10 FDR threshold to 0.25 produced 253 associations rather than 121, though the overlap with the 90 pGENMi associations only increased to 6 (Hypergeometric p-value = 0.63). We further diluted the statistical reliability of the TWAS associations by reporting associations based on uncorrected p-values <= 0.05. Of the 384 associations matching this criteria (among the 3552 test), the overlap with pGENMi yielded only 10 associations (Hypergeometric p-value = 0.51).

Cell line specific ChIP data and Composite ChIP data alter pGENMi associations

As the genotype, methylation, gene expression, and cytotoxicity data all were derived from the same panel of cell lines, we inquired whether restricting ChIP peaks to those from lymphoblastoid cell lines produced different results than when using a union of clustered peaks across cell lines (‘composite ChIP’ data), which was the strategy used for the analyses above. To test this, we ran pGENMi using eQTL data co-incident with GM12878 ChIP peaks from ENCODE and compared the results to the eQTL-only analysis using composite ChIP peaks. Among the 37 GM12878 specific TFs and 24 drugs tested, we tested 888 (TF, Drug) associations, of which 90 pairs exhibited an LLR >= 1.74. At the same threshold, and when testing the same candidate pairs, the composite ChIP analysis produced 29 associations, with only three associations – (ELF1, 6-MP), (MAX, 6-MP), and (NFIC, 6-TG) – being common with the analysis based on GM12878 peaks. We noted that pairs with LLR >= 1 in composite ChIP analysis had a greater tendency (compared with those with LLR < 1) to be in the significant list of the GM12878 ChIP analysis (t-test p-value 3.05E-04, see **Supplemental Fig. S14**). Overall, our conclusion from this comparison is that the top associations reported by pGENMi depend significantly on the source of the ChIP data use to

infer regulatory evidence, which is expected. Our use of composite (multiple cell line) ChIP data in the results reported in this work was motivated by our search for associations that generalize beyond lymphoblastoid cell lines, to other cancer cell lines where many of the associations validated in the literature as well in this study have been demonstrated.

It is notable that the GM12878 specific analyses produced a substantially greater number of associations above the reporting threshold. One reason for this may lie in the difference between the GM12878 and Composite ChIP analyses in terms of the number of target genes (those with regulatory evidence) for each TF. For instance, the median number of target genes with cis-eQTL evidence for a TF's influence is 58 per TF and 151 per TF for Composite and GM12878 ChIP data respectively; the mean number of target genes per TF based on either cis-eQTL or cis-eQTM (eQTL+M) evidence improves to 102 for the composite ChIP data and 155 for the GM12878 data. The detection discrepancy between the two analyses that leads to fewer targets in the Composite ChIP analysis may be due, in part, to the clustering across cell lines which dilutes LCL specific ChIP signals and high occupancy target (HOT) region removal. It does not seem exceptional that LCL derived eQTLs would be more enriched in LCL specific ChIP peaks. Another reason for the GM12878 specific analyses exhibiting may be the asymmetric distribution of TFs associated with drugs in the analysis using GM12878 ChIP data. As shown in **Supplemental Table S4,** the 4 drugs 6-MP, Doxorubicin, Epirubicin, 6-TG account for 67 of the 90 (TF, Drug) associations observed at a LLR >= 1.74. Two of these drugs – 6-MP and 6-TG – also had among the largest number of TWAS genes, which together with the larger numbers of target genes designated with GM12878 ChIP data may have resulted in the inflated numbers of TF associations. The enrichment of associations for these drugs may also indicate a greater sensitivity to them in this cell line.

We maintain that the Composite ChIP analysis offers a more balanced and generalizable view of the regulatory landscape for a TF and thus is more robust to validation in other cell lines and tissues. We leave more in depth analysis of the utility of cell line specific ChIP data for future research.

Potential factors leading to false positives in pGENMi predictions

Validation rates of pGENMi predictions, based on literature search or our own experiments, were around 40% overall, which is far from perfect, and highly variable across drugs. One of the potential reasons for false positives is the great variation among drugs in terms of the number of significant TWAS genes, i.e., genes with significant correlation between their expression and cytotoxicity. We tabulated the number of significant TWAS genes alongside the number of pGENMi eQTL+M associations (LLR >= 3) for each drug in **Supplemental Table S6**. The five drugs with most reported associations (6-MP, NAPQI, Temozolomide, Cisplatin, Ara-C) - about 10 TFs on average - show far more TWAS genes (median of 227), compared to the remaining 19 drugs, which typically yield about 1 TF association (median) and have only 27 TWAS genes (median); see **Supplemental Table S7.** This raises the possibility that pGENMi makes false positive predictions in cases when there are significantly more phenotype-associated genes. Pursuing this further, we noted that for the top five drugs by number of associations, our literature-based validation (**Table 2**) found direct evidence for only 10 of 35 (29%) predicted TF associations, while for the remaining 19 drugs we were able to find similar direct evidence for 20 of 38 (53%) TF associations predicted by pGENMi, a statistically significant difference (Hypergeometric test p-value 0.032). Similar observations were made on our own experimental validations (**Table 4A**), though small sample sizes prevent statistical claims. For instance, two of the above-mentioned 'top 5' drugs had 3 or more TFs tested by us (6-MP and Temozolomide), and both yielded low validation rates (0/3 and 1/4 respectively), while the drug Rapamycin, for

which only 3 predictions were made and all three were tested by us, yielded a success rate of 3/3. In light of the above observations, we believe that the accuracy of pGENMi predictions may suffer when a phenotype variation is correlated with a large number of genes' expression levels, and future work should attempt to rectify this issue if possible.

Another possible factor leading to false positives is that extensive co-binding of a pair of TFs leads to the method mistaking the co-bound TF for the true regulator. We have tried to address this potential source of false positives by removing HOT regions where most co-binding is observed from our analysis (see **Supplemental Note S1)**. Many TF pairs are known to exhibit significant co-binding even after HOT region removal, as demonstrated by the ENCODE project, and thus it is possible that some of the false positives arise from co-binding. In such cases, if the true TF is among those tested by us (i.e., has ENCODE ChIP data), then we would expect that it should also score highly in the pGENMi analysis. For example, in our experimental validation, we predicted that ELF1 and ZNF263 were both associated with temozolomide, but only succeeded in validating ELF1 through cytotoxicity assays, while ZNF263 knockdown did not show significant change in response. ENCODE reports that these two TFs co-bind across the whole genome in K562 cells (The Encode Project Consortium 2012), so it is possible that the predicted ZNF263 association was a false positive because of the true ELF1 association and the ELF1-ZNF263 co-binding.

Finally, with regards to the experimental validation, some (TF, Drug) pairs didn't validate *in vivo* because of the assay design; (FOXM1, Temozolomide) and (PML, Radiation) were validated directly in tissues according to the literature, but both failed to validate in vivo in cell lines. Furthermore, we validated (FOXM1, Temozolomide) in recurrent glioblastoma multiforme (GBM) tumor but failed to validate in U251 – which is derived from completely different tissue than GBM.

The same holds for (PML, Radiation), which was validated most strongly in mouse embryonic fibroblasts (MEFs), and which failed in a triple negative breast cancer cell line (MDA-MB-231) completely unlike MEFs. Because (TF, Drug) pairs may be context (cell-line) specific, failure to validate in certain cell lines cannot be interpreted necessarily as a rejection of the relationship altogether, as the two examples above show.

pGENMi comparison to GPA

The Genomic Pleiotropy with Annotation (GPA) (Chung et al. 2014) algorithm is similar in spirit to pGENMi. GPA uses latent variables for SNPs and integrates the GWAS p-value of a SNP with annotations of that SNP; pGENMi in contrast uses latent variables for genes and integrates the TWAS p-value a gene with annotations of that gene based on eQTL, eQTM and TF ChIP peak evidence. The commonality between the two models, is thus at a higher conceptual level rather than at a practical level that might warrant empirical comparisons. Even at the technical level there is a key difference in that GPA models the joint likelihood of the annotations and GWAS p-value of SNPs, whereas we only use regulatory evidence to maximize the likelihood of TWAS data.

Supplemental Note S4: Data pre-processing and statistical methodology

Removing Transcription Factors for GENMi

Some TFs (ATF1, BCLAF1, MEF2C, PPARGC1A, SMARCC2, STAT2) were filtered out of the GENMi analysis for having an insufficient number of target genes (<15) for GSEA.

Removing HOT Regions from ENCODE TF ChIP data

We used TF ChIP peaks as a way to focus on SNPs whose association with expression might be mediated by the regulatory action of that TF. However, it is well known that different TFs tend to co-localize at the same genomic loci, a phenomenon that is especially pronounced at "HOT" (high occupancy target) regions, and a TF's binding at these HOT regions is not necessarily indicative of regulatory function (mod Encode Consortium et al. 2010). To enrich the ChIP-based collection of binding sites for functional TF-DNA interactions, we removed segments of 50bp where six or more TFs bind, resulting in a ~25% reduction in the total number of ChIP TF peaks across all ENCODE cell lines.

SNP Imputation

Imputation was run separately for each race and chromosome separately. Chromosomes were divided into 40MB regions. BEAGLE v3.3.1 (Browning and Yu 2009) was run on these 40MB regions of the genome, plus a 1MB buffer region to the right and left of the main region, as the ends are generally imputed poorly. The reference and observed genotype data were input as phased and unphased, respectively. The lowmem option was enforced to reduce the overall amount of memory required to run BEAGLE; additionally, the exclude markers option was used to remove the rare SNPs from the reference mentioned above. For edification, BEAGLE imputed untyped markers, not missing genotyped markers.

Summarizing probe expression by gene symbol

Rather than modeling raw probe data, we opted to summarize probe level expression at the Ensembl gene symbol level, since representing gene expression using multiple correlated probes breaks the independence assumptions of our probabilistic model by giving greater weight to genes with higher probe coverage. Of the 54,613 probes, those with low variance ($\sigma^2 \leq 0.1$), were omitted from analysis, purging 5,408 probes. Of the remainder, 34,832 probes mapped to 16,183 stable Ensembl gene symbols with at least one exon annotation, using the GRCh37 Ensembl BioMart mapping of Ensembl gene symbols with HGU 133 Plus 2.0 array probe identifiers. Since multiple probes can map to the same gene, we utilized a simple algorithm to obtain a single gene expression value as a function of its mapped probe expressions. The following illustrates this procedure, which uses principle component analysis (PCA) to obtain a vector of gene expression and Z-score normalization to ensure all genes are scaled identically:

For each gene g with at least one mapped probe:

1. If g has one mapped probe
    a. Return z-score normalization of the probe's expression.
2. If gene g has n mapped probes with a probe expression matrix of size *n* x 284:
    a. Obtain the first principal component (PC_1) of the probe expression matrix.
    b. Project the probe expression matrix onto PC_1.
    c. Return z-score normalization of the projected PC_1 expression.

Applying this procedure resulted in a matrix of Ensembl gene expression with dimensionality 16,183 (number of genes) x 284 (number of LCLs), and Z-score normalized expression across

individuals. Henceforth in this publication, we refer to this matrix and its values as the gene expression matrix and gene expression, respectively.

Controlling for confounding variables

We included the following potentially confounding variables as covariates in all regression analyses for this study: batch, age, gender, and sub-population labels, derived from EIGENSTRAT (see below). We decided to omit explicit labels of ethnicity, as the sub-population labels should capture that information implicitly. To derive p-values for the covariate of interest in the multiple regression (for instance, a SNP in an eQTL regression) we computed the p-value of the log likelihood ratio between models with and without the covariate of interest, using a χ^2 distribution.

Population stratification

Despite the information provided by ethnic labels, it is well known that sub-population structures within these ethnic groups contain important information that can radically confound the results of association analyses when ignored or improperly controlled (Price et al. 2006). To address this issue, we utilized the EIGENSTRAT program (version 6.0.1) developed by the Price lab (https://www.hsph.harvard.edu/alkes-price/software), to identify sub-population labels from genotype data and remove their potential confounding effects in regression analyses. Instrumental to EIGENSTRAT's ability to recover true population labels is the quality and format in which the data are provided. It may conflate large genomic regions in linkage disequilibrium (LD) with population structure, hence such regions have to be pruned. Additionally, while EIGENSTRAT could identify latent population labels corresponding to the cohort's ethnicities from the entire cohort's genotype matrix, it is more useful run EIGENSTRAT on each known ethnic

group's genotype matrix: doing so will direct EIGENSTRAT to look for sub-populations only within each ethnicity, and not across.

To perform this analysis, we first computed a set of independent SNPs using the PLINK program (1.90 beta) (Chang et al. 2015). For each ethnicity (HCA, CA, and AA), we used a variant pruning algorithm to remove redundant SNPs in LD and reported only independent SNPs on somatic chromosomes. To generate these results, we ran PLINK with the '-indep' flag and the following parameters:

1. Window size (SNPs): 50
2. Shift window (SNPs): 5
3. Variance Inflation Factor (VIF) threshold: 1

Using these parameters, PLINK slides a window across the genome and only retains those SNPs in the window that cannot be adequately predicted from a linear combination of the remaining SNPs. For each SNP s in a window, PLINK reports a goodness of fit, R^2_s, of the multivariate regression to predict s from all other SNPs. PLINK retains only those SNPs with R^2 >= 1 – 1/VIF. Setting VIF to 1 ensures that only statistically independent SNPs in each window are selected; although strict, this removes all potential for LD confoundment, at the potential cost of underestimating the number of sub-populations.

We estimated these sub-population labels as continuous axes of variation by using EIGENSTRAT to perform PCA on each ethnic group's independent SNP genotype matrix. Each significant principle component (PC) derived from the genotype matrix of a given ethnicity can be interpreted as a sub-population of that ethnicity. For each ethnicity, we retrieved all PCs with Tracy-Widom p-values $\leq$ 0.05, resulting in a total of seven axes, shown in **Supplemental Table S1**. We

projected the entire genotype matrix onto each of the seven axes (setting to 0 SNPs not contributing to the axis) to derive numeric sub-population representations for the cohort. We included these sub-population labels as covariates in our regression models to control for population stratification.

Supplemental Note S5: Experimental validation design, data, methodology, and statistical analysis

Removing Common Transcription Factors for Experimental Validation

In experimental validation, we avoided TFs like *BRF2* and *GTF2F1* that were associated with 10 or more drugs. These are general transcription factors whose association is likely due to having many more ChIP peaks than other TFs, a point we have discussed in previous work (Hanson et al. 2015). We also excluded NAPQI, a toxic byproduct produced during the xenobiotic metabolism of the analgesic paracetamol, due to its lack of clinical application.

Cell culture and treatments

Human triple negative breast cancer (MDA-MB231), leukemia (Jurkat), and glioma (U251) cell lines were obtained from the American Type Culture Collection (Manassas, VA). MDA-MB-231 cells were cultured in L-15 medium containing 10% Fetal Bovine Serum (FBS). Jurkat cells were cultured in Roswell Park Memorial Institute (RPMI) 1640 Medium, containing 10% FBS. U251 cells were cultured in Dulbecco's Modified Eagle Medium (DMEM), also containing 10% FBS.

The following 10 drugs were purchased from Sigma-Aldrich (St. Louis, MO): 6-Mercaptopurine (6-MP), 6-Thioguanine (6-TG), carboplatin, cisplatin (CDDP), cytarabine (Ara-C), docetaxel, epirubicin, gemcitabine, oxaliplatin, and paclitaxel. The remaining three drugs were obtained Selleck Chemicals (Houston, TX): cladribine, rapamycin (sirolimus), and temozolomide (TMZ). All 13 drugs were dissolved in dimethyl sulfoxide (DMSO) and aliquots of stock solutions were frozen at -80°C.

RNA interference and Real-time quantitative reverse transcription-PCR (qRT-PCR)

siRNAs for candidate TFs and negative control siRNA were purchased from Dharmacon. Reverse transfection was performed for MDA-MB231 and U251 cells in 96-well plates. Specifically, 3000-4000 cells were mixed with 0.1 mL of lipofectamine RNAi-MAX reagent (Invitrogen) and 10 nM siRNA for each experiment. Electroporation was performed for Jurkat cells using Nucleofector® Kit V from Lonza (Cologne, Germany).

Prior to electroporation, cells were washed with phosphate buffer saline (PBS) and counted. One million Jurkat cells were re-suspended in 100 µL of the Nucleofector® Solution buffer and mixed with 100 nM of specific siRNA. The re-suspended cells were transferred to cuvettes and immediately electroporated using the program X-005. After electroporation, cells were incubated in a cuvette at room temperature for 10 minutes and then 500 µL of pre-warmed culture medium were added to the cuvette. Cells were then transferred to a 12-well plate and incubated at 37°C/5% CO_2 overnight.

Total RNA was isolated from cultured cells transfected with control or TF-specific siRNAs with the Qiagen RNeasy kit (QIAGEN, Inc.), followed by qRT-PCR performed with the one-step Brilliant SYBR Green qRT-PCR master mix kit (Stratagene). Specifically, primers purchased from QIAGEN were used to perform qRT-PCR using the Stratagene Mx3005P Real-Time PCR detection system (Stratagene). All experiments were performed in triplicate with beta-actin as an internal control. Reverse transcribed Universal Human reference RNA (Stratagene) was used to generate a standard curve. Control reactions lacked RNA template.

MTS cytotoxicity assay

Cell proliferation assays were performed in triplicates at each drug concentration. Cytotoxicity assays with the lymphoblastoid and tumor cell lines were performed in triplicates at each dose.

Specifically, 90 μL of cells (5 × 10^4 cells) were plated into 96-well plates (Corning, NY) and were treated with increasing does of a specific drug or radiation. After incubation for 72 hours, 20 μL of CellTiter 96® AQueous Non-Radioactive Cell Proliferation Assay solution (Promega Corporation, Madison, WI) was added to each well. Plates were read in a Safire2 plate reader (Tecan AG, Switzerland).

Cytotoxicity assays with the tumor cell lines were performed using the CellTiter 96® AQueous Non-Radioactive Cell Proliferation Assay (Promega Corporation, Madison, WI). Specifically, 90 μL of cells (5 × 10^3 cells) were plated into 96-well plates and were treated with increasing does of a specific drug. The escalation of concentrations for each drug is listed in **Supplemental Table S3**. After incubation for 72 hours, 20 μL of CellTiter 96® AQueous Non-Radioactive Cell Proliferation Assay solution (Promega Corporation, Madison, WI) was added to each well. Plates were read in a Safire2 plate reader (Tecan AG, Switzerland). Cytotoxicity was assessed by plotting cell survival versus drug concentration, on a log scale.

Radiation cytotoxicity was performed in triplicates at each radiation dose as described above. 100 μL of cells (5 × 10^3 cells) were plated into 96-well plates and were treated with ionizing radiation at 0, 0.25, 0.5, 1, 2.5, 5, 10, and 20 Gy, using cesium-137 gamma-rays (J.L. Shepherd and Associates Mark I Model 25 Irradiator). After incubation for 72 hours, 20 μL of CellTiter 96 AQueous Non-Radioactive Cell Proliferation Assay solution was added to each well. Plates were read in a Safire2 plate reader (Tecan AG).

Statistical analysis

Significance of the IC50 values between negative control siRNA and TF-specific siRNA was determined using a two-tailed unpaired t-test.

Supplemental Note S6: Expectation Maximization Formulation for PGM and Implementation Details

Definitions:

M := Number of regulatory evidences + 1.

G := Number of genes.

$\mathbf{R}$:= A matrix of size $G \times M$, where the first columns is all 1's and each additional column a binary corresponding to whether regulatory evidence was observed for that column's source.

$\mathbf{r_g}$:= A vector of length M with the first column as 1 and each additional column a binary corresponding to whether regulatory evidence was observed for that column's source.

$\mathbf{w}$:= A continuous vector of length M that weights each type of regulatory evidence. This is a parameter estimated by the model across all genes.

z_g:= A binary latent variable representing whether or not the correlation p-value of gene $g's$ expression with the phenotype of interest is drawn from a uniform or beta distribution.

α := Parameter determining the shape of the beta distribution. The β parameter is set to 1 and α is capped in the range [0,1]. This parameter is estimated by the model.

p_g:= A continuous observed variable in the range [0,1] representing the correlation p-value of gene $g's$ expression with the phenotype of interest

Likelihood

The probability that $z_g = 1$ is a logistic function of $\mathbf{w}$ and $\mathbf{r_g}$**:**

$$\Pr(z_g = 1|\mathbf{w}, \mathbf{r_g}) = \frac{1}{1 + \exp(-\mathbf{w} \cdot \mathbf{r_g})}$$

The probability that $z_g = 0$ is 1 – probability $z_g = 1$:

$$\Pr\left(z_g = 0|\mathbf{w}, \mathbf{r_g}\right) = \frac{\exp\left(-\mathbf{w}\cdot\mathbf{r_g}\right)}{1+\exp\left(-\mathbf{w}\cdot\mathbf{r_g}\right)}$$

If you interpret 1 as $\exp(-0\cdot\mathbf{r_g})$, then this is simply the Boltzman distribution with the 2nd state being $\mathbf{w} = \mathbf{0}.$

The probability of p_g depends on if z_g is 1 or 0. With respect to the former, it is distributed per a beta distribution (with $\beta = 1$) and uniform distribution for the latter.

$$\Pr\left(p_g|z_g = 1, \alpha\right) \sim \mathrm{Beta}(\alpha, 1)$$

$$\Pr\left(p_g|z_g = 0, \alpha\right) \sim \mathrm{Unif}(0,1)$$

Aside:

$$Beta(\alpha, 1) = \frac{x^{\alpha-1}}{\mathrm{B}(\alpha,1)} = \frac{x^{\alpha-1}}{\Gamma(\alpha)}\Gamma(\alpha+1) = \alpha\frac{\Gamma(\alpha)}{\Gamma(\alpha)}x^{\alpha-1} = \alpha x^{\alpha-1}$$

Therefore,

$$\Pr\left(p_g|z_g = 1, \alpha\right) \sim \mathrm{Beta}(\alpha, 1) = \alpha\, x^{\alpha-1}$$

The likelihood of the data $\mathbf{p}$ given $\mathbf{R}$ is thus.

$$\Pr(\mathbf{p}|\mathbf{w}, \mathbf{R}, \alpha) = \prod_{g=1}^{G}\sum_{i=0}^{1}\Pr(p_g|z_g = i, \alpha) * \Pr(z_g = i|\mathbf{w}, \mathbf{r_g})$$

Expanding the terms and the sum yields the following:

$$\Pr(\mathbf{p}|\mathbf{w}, \mathbf{R}, \alpha) = \prod_{g=1}^{G}\alpha p_g^{\alpha-1} * \frac{1}{1+\exp\left(-\mathbf{w}\cdot\mathbf{r_g}\right)} + \frac{\exp\left(-\mathbf{w}\cdot\mathbf{r_g}\right)}{1+\exp\left(-\mathbf{w}\cdot\mathbf{r_g}\right)}$$

The log likelihood is thus:

$$l(\mathbf{p}|\mathbf{w}, \mathbf{R}, \alpha) = \sum_{i=1}^{G}\log\left(\alpha p_g^{\alpha-1} * \frac{1}{1+\exp\left(-\mathbf{w}\cdot\mathbf{r_g}\right)} + \frac{\exp\left(-\mathbf{w}\cdot\mathbf{r_g}\right)}{1+\exp\left(-\mathbf{w}\cdot\mathbf{r_g}\right)}\right)$$

We can either optimize this function directly or use Expectation Maximization. In this formulation, we choose EM.

EM Posterior Probability:

The posterior probability for $z_g = 1$ is the following:

$$\Pr\left(z_g = 1 \middle| p_g, \alpha, \mathbf{w}, \mathbf{r_g}\right) = \frac{\Pr(p_g, z_g = 1, \alpha, \mathbf{w}, \mathbf{r_g})}{\Pr(p_g, \alpha, \mathbf{w}, \mathbf{r_g})} = \frac{\Pr(p_g, z_g = 1, \alpha, \mathbf{w}, \mathbf{r_g})}{\Pr(p_g, z_g = 0 \alpha, \mathbf{w}, \mathbf{r_g}) + \Pr(p_g, z_g = 1 \alpha, \mathbf{w}, \mathbf{r_g})}$$

$$\Pr(p_g, z_g = 1, \alpha, \mathbf{w}, \mathbf{r_g}) = \alpha p_g^{\alpha-1} * \frac{1}{1 + \exp(-\mathbf{w} \cdot \mathbf{r_g})}$$

$$\Pr(p_g, z_g = 1, \alpha, \mathbf{w}, \mathbf{r_g}) = \frac{\exp(-\mathbf{w} \cdot \mathbf{r_g})}{1 + \exp(-\mathbf{w} \cdot \mathbf{r_g})}$$

$$\Pr\left(z_g = 1 \middle| p_g, \alpha, \mathbf{w}, \mathbf{r_g}\right) = \frac{\frac{\alpha p_g^{\alpha-1}}{1 + \exp(-\mathbf{w} \cdot \mathbf{r_g})}}{\frac{\alpha p_g^{\alpha-1}}{1 + \exp(-\mathbf{w} \cdot \mathbf{r_g})} + \frac{\exp(-\mathbf{w} \cdot \mathbf{r_g})}{1 + \exp(-\mathbf{w} \cdot \mathbf{r_g})}}$$

$$\Pr\left(z_g = 1 \middle| p_g, \alpha, \mathbf{w}, \mathbf{r_g}\right) = \frac{\alpha p_g^{\alpha-1}}{\alpha p_g^{\alpha-1} + \exp(-\boldsymbol{w} \cdot \boldsymbol{r_g})}$$

Naturally, the posterior probability for $z_g = 0$ follows as 1 – posterior probability of $z_g = 1$:

$$\Pr\left(z_g = 0 \middle| p_g, \alpha, \mathbf{w}, \mathbf{r_g}\right) = \frac{\exp(-\boldsymbol{w} \cdot \boldsymbol{r_g})}{\alpha p_g^{\alpha-1} + \exp(-\boldsymbol{w} \cdot \boldsymbol{r_g})}$$

Expectation Maximization

The function that EM optimizes is the $Q(\boldsymbol{\theta}|\boldsymbol{\theta}^{t-1})$ function where $\boldsymbol{\theta} = \{\mathbf{w}, \alpha\}$. It is essentially the expectation of the joint log likelihood under the current parameters $\boldsymbol{\theta}$ at time t with respect to the posterior computed using the previous parameters $\boldsymbol{\theta}^{t-1}$**.**

$$Q(\boldsymbol{\theta}|\boldsymbol{\theta}^{t-1}) = \sum_{g=1}^{G} \sum_{i=0}^{1} \Pr\left(z_g = i \,\middle|\, p_g, \alpha^{t-1}, \mathbf{w}^{t-1}, \mathbf{r_g}\right) \log(\Pr(p_g, z_g = i, \alpha, \mathbf{w}, \mathbf{r_g}))$$

We will let the posteriors be designated as follows:

$$z_{g0}^{t-1} = \Pr\left(z_g = 0 \,\middle|\, p_g, \alpha^{t-1}, \mathbf{w}^{t-1}, \mathbf{r_g}\right)$$

$$z_{g1}^{t-1} = \Pr\left(z_g = 1 \,\middle|\, p_g, \alpha^{t-1}, \mathbf{w}^{t-1}, \mathbf{r_g}\right)$$

Expanding terms gives the following:

$$Q(\boldsymbol{\theta}|\boldsymbol{\theta}^{t-1}) = \sum_{g=1}^{G} z_{g1}^{t-1} * \log\left(\Pr\left(p_g|z_g = 1, \alpha\right) * \Pr\left(z_g = 1|\mathbf{r_g}, \mathbf{w}\right)\right)$$

$$+ \; z_{g0}^{t-1} \log\left(\Pr\left(p_g|z_g = 0, \alpha\right) * \Pr\left(z_g = 0|\mathbf{r_g}, \mathbf{w}\right)\right)$$

Further reducing gives:

$$Q(\boldsymbol{\theta}|\boldsymbol{\theta}^{t-1}) = \sum_{g=1}^{G} z_{g1}^{t-1} \log\left(\alpha p_g^{\alpha-1} * \frac{1}{1+\exp\left(-\mathbf{w}\cdot\mathbf{r_g}\right)}\right) + z_{g0}^{t-1} \log\left(\frac{\exp\left(-\mathbf{w}\cdot\mathbf{r_g}\right)}{1+\exp\left(-\mathbf{w}\cdot\mathbf{r_g}\right)}\right)$$

Further reducing gives:

$$Q(\boldsymbol{\theta}|\boldsymbol{\theta}^{t-1}) = \sum_{g=1}^{G} z_{g1}^{t-1} \log\left(\alpha p_g^{\alpha-1}\right) - z_{g0}^{t-1}(\mathbf{w}\cdot\mathbf{r_g}) \; - \log\left(1+\exp\left(-\mathbf{w}\cdot\mathbf{r_g}\right)\right)$$

Update $\mathbf{w}$*:*

When maximizing, we assume z_{g0}^{t-1} and z_{g1}^{t-1} are fixed. Therefore, finding a formula for $\mathbf{w}$:

$$\frac{\partial Q}{\partial \mathbf{w}} = \sum_{g=1}^{G} \mathbf{r_g} * \left(\frac{\exp\left(-\mathbf{w}\cdot\mathbf{r_g}\right)}{1+\exp\left(-\mathbf{w}\cdot\mathbf{r_g}\right)} - z_{g0}^{t-1}\right)$$

Setting to 0 doesn't yield a closed form solution, so we use this gradient in a gradient descent for updating $\mathbf{w}$.

$$\mathbf{w^t} = \mathbf{w}^{t-1} - \beta * \frac{\partial Q}{\partial \mathbf{w}}$$

For this study, we converged when $\left|\left|\mathbf{w^t} - \mathbf{w}^{t-1}\right|\right|_{\mathbf{2}} < \epsilon$

Update α:

The parameter α has a closed form solution. Given

$$Q(\boldsymbol{\theta}|\boldsymbol{\theta}^{t-1}) = \sum_{g=1}^{G} z_{g1}^{t-1} \log\left(\alpha p_g^{\alpha-1}\right) - z_{g0}^{t-1}(\mathbf{w}\cdot\mathbf{r_g}) \; - \log\left(1+\exp\left(-\mathbf{w}\cdot\mathbf{r_g}\right)\right)$$

Optimizing for α gives:

$$\frac{\partial Q}{\partial \alpha} = \sum_{g=1}^{G} z_{g1}^{t-1} \frac{\partial}{\partial \alpha} (\log(\alpha) + (\alpha - 1)\log(p_g))$$

$$\frac{\partial Q}{\partial \alpha} = \sum_{g=1}^{G} z_{g1}^{t-1} \left(\frac{1}{\alpha} + \log(p_g)\right)$$

Setting to 0 and solving for α gives the following:

$$0 = \sum_{g=1}^{G} \frac{1}{\alpha} z_{g1}^{t-1} + \log(p_g) z_{g1}^{t-1}$$

$$-\frac{1}{\alpha} \sum_{g=1}^{G} z_{g1}^{t-1} = \sum_{g=1}^{G} z_{g1}^{t-1} \log(p_g)$$

$$\alpha = -\frac{\sum_{g=1}^{G} z_{g1}^{t-1}}{\sum_{g=1}^{G} z_{g1}^{t-1} \log(p_g)}$$

Log Likelihood Ratio:

We compare the fit model, $H = 1$, to a model where $\mathbf{w} = \mathbf{0}$. We indicate this with, $H = 0$, indicating the null hypothesis. We assess the significance of the alternative model, $H = 1$, by computing the log likelihood ratio between a fit model and the null model.

$$LLR = \log(Pr(\boldsymbol{p}|\boldsymbol{w}, \boldsymbol{R}, \alpha, H = 1)) - \log(Pr(\boldsymbol{p}|\boldsymbol{w}, \boldsymbol{R}, \alpha, H = 0))$$

Implementation:

In our implementation of EM, we choose $\beta = 0.0001$ and $\epsilon = 0.0001$ with a minimum and maximum of 25 and 300 steps in each EM calculation and 100 random restarts for each model.

For each random restart, we estimated an alternative and null model. We report the LLR corresponding to the model with the best log-likelihood for the alternative model.

TensorFlow Implementation:

We also have a TensorFlow implementation available for download, although this implementation was not used to generate the results of this paper.

Code Location:

Both the C++ code used to generate the results of this paper and the TensorFlow code are available in a GitHub repository located at https://github.com/knoweng/pgenmi and with a link to the GitHub repository at veda.cs.uiuc.edu/pgenmi . The GitHub repo is also available in **Supplemental Code S1.**

Supplemental Fig. S1: Cytotoxicity experiments performed in Jurkat cell lines treated with 6-MP. Of the 5 TFs tested, one was validated. In this case, the validated TF, EZH2 (outlined in red), and FOXP2 were eQTL+M negative controls, while the other 3 were eQTL+M predictions.

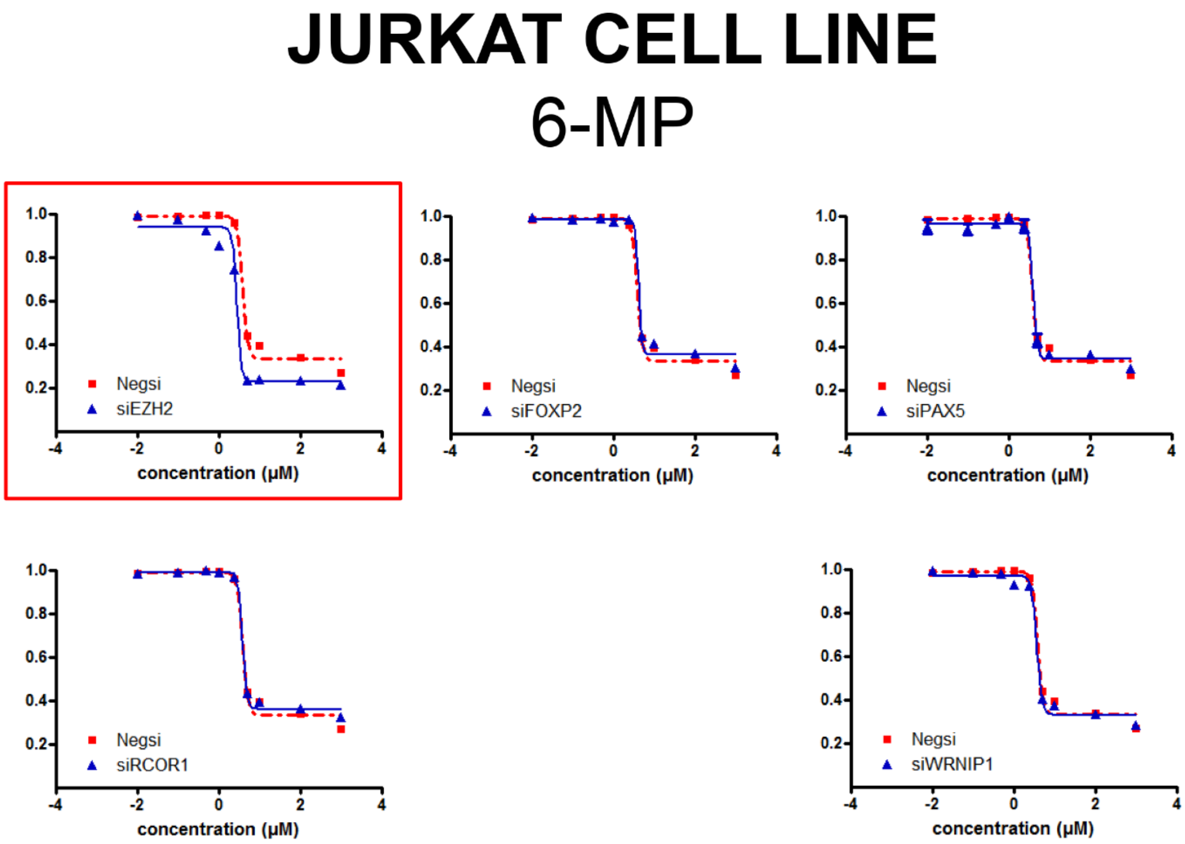

Supplemental Fig. S2: Cytotoxicity experiments performed in Jurkat cell lines treated with 6-TG. Both TFs were eQTL+M negative controls and failed validation.

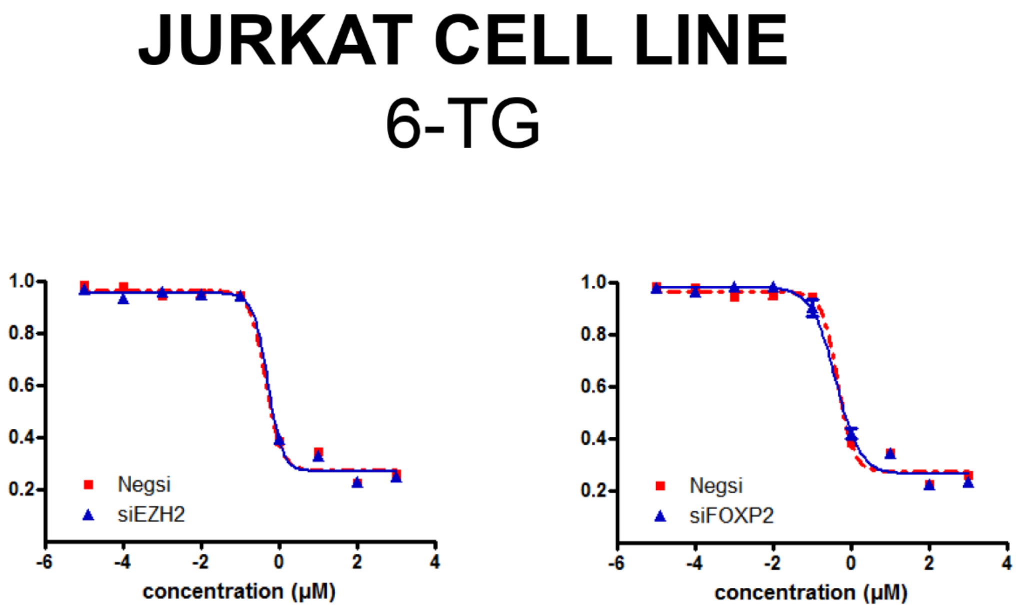

Supplemental Fig. S3: Cytotoxicity experiments performed in Jurkat cell lines treated with Ara-C. Both TFs were eQTL+M predictions. *RAD21* was successfully validated, indicated by the red outline, while *UBTF* failed validation.

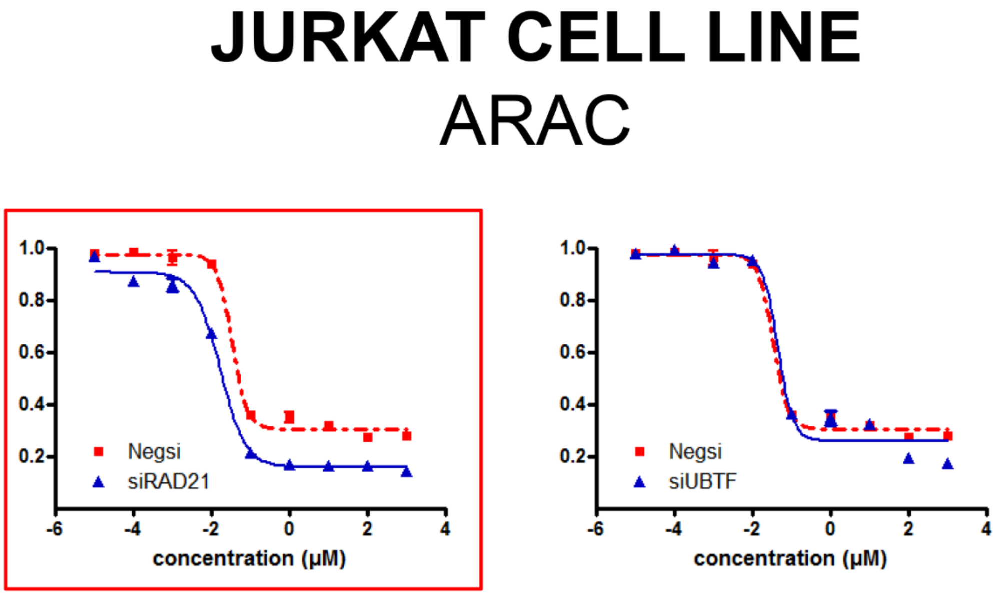

Supplemental Fig. S4: Cytotoxicity experiments performed in MDA-MB-231 cell lines treated with carboplatin. The TF failed to validate and was an eQTL+M negative control.

MDA-MB-231 CELL LINE
CARBOPLATIN

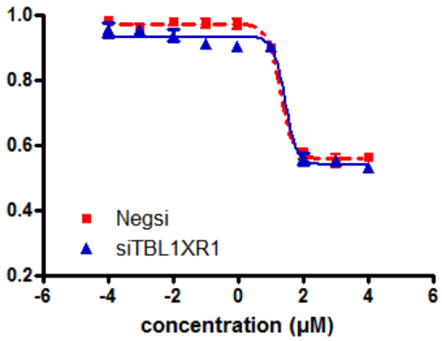

Supplemental Fig. S5: Cytotoxicity experiments performed in MDA-MB-231 cell lines treated with cisplatin (CDDP). Of the 2 TFs tested, *CEBPD*, which was an eQTL+M prediction (as indicated by the red outline), was validated, while *EZH2*, which was a negative control, failed to be validated.

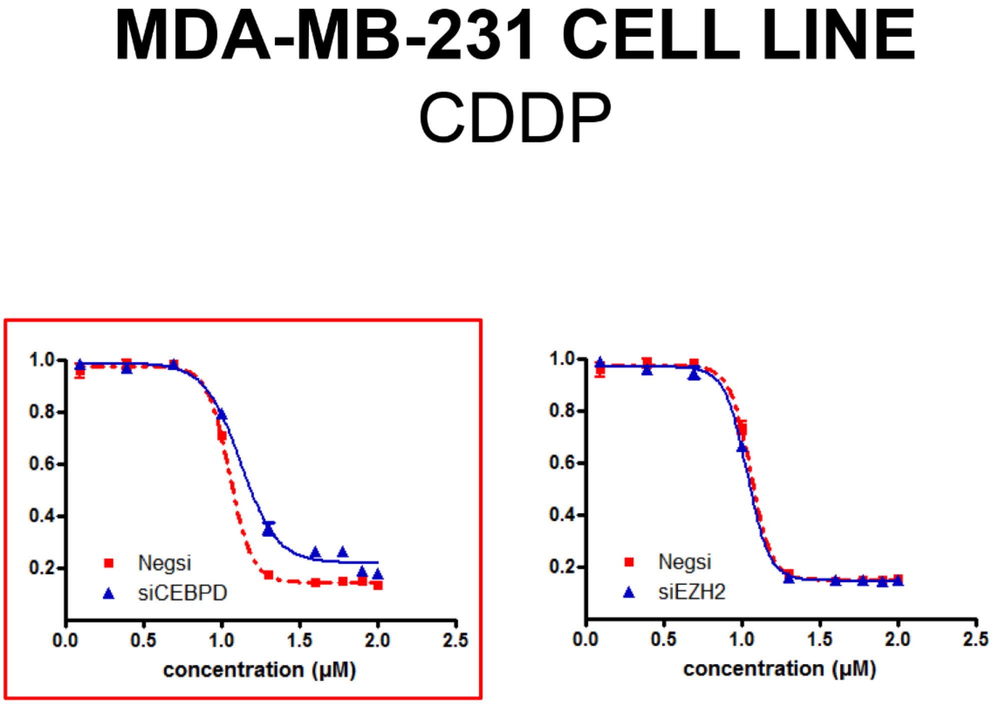

Supplemental Fig. S6: A Cytotoxicity experiment performed in MDA-MB-231 cell lines treated with epirubicin. The TF, which was an eQTL+M prediction, failed to validate.

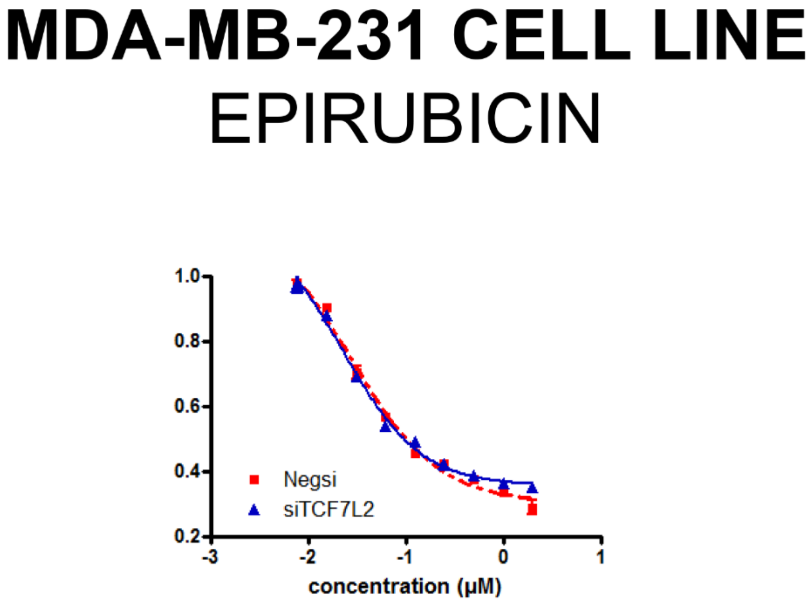

Supplemental Fig. S7: A Cytotoxicity experiment performed in MDA-MB-231 cell lines treated with gemcitabine. The TF, which was an eQTL+M prediction, failed to validate.

MDA-MB-231 CELL LINE
GEMCITABINE

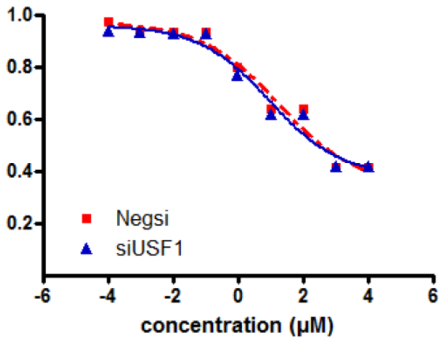

Supplemental Fig. S8: A Cytotoxicity experiment performed in MDA-MB-231 cell lines treated with oxaliplatin. The TF, which was an eQTL+M negative control, failed to validate.

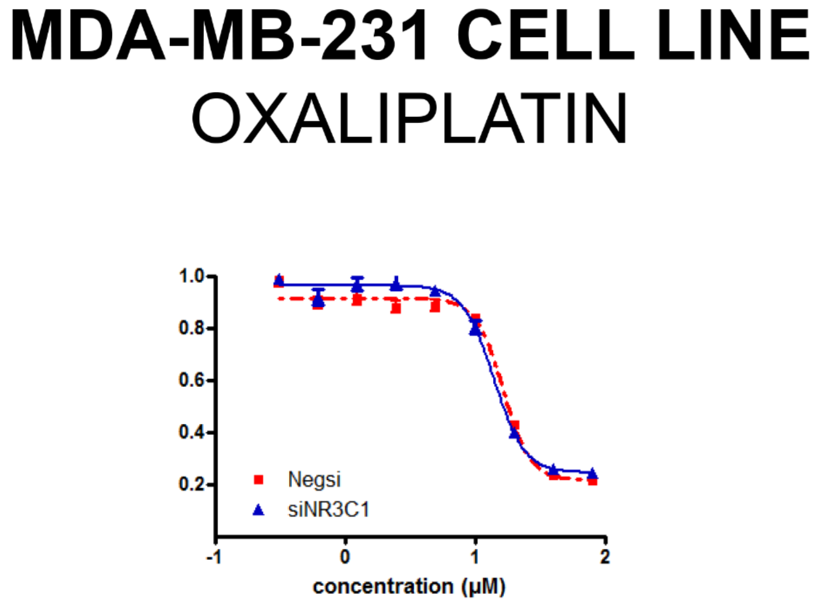

Supplemental Fig. S9: A Cytotoxicity experiment performed in MDA-MB-231 cell lines treated with paclitaxel. The TF, which was an eQTL+M negative control, failed to validate.

MDA-MB-231 CELL LINE

PACLITAXEL

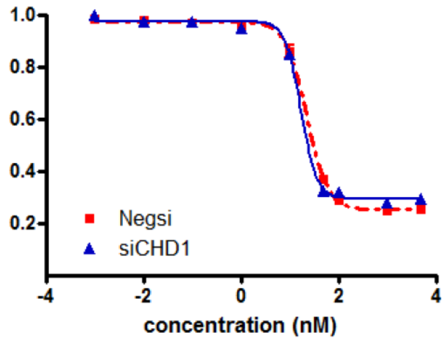

Supplemental Fig. S10: A Cytotoxicity experiment performed in MDA-MB-231 cell lines treated with radiation. The TF, which was an eQTL+M prediction, failed to validate.

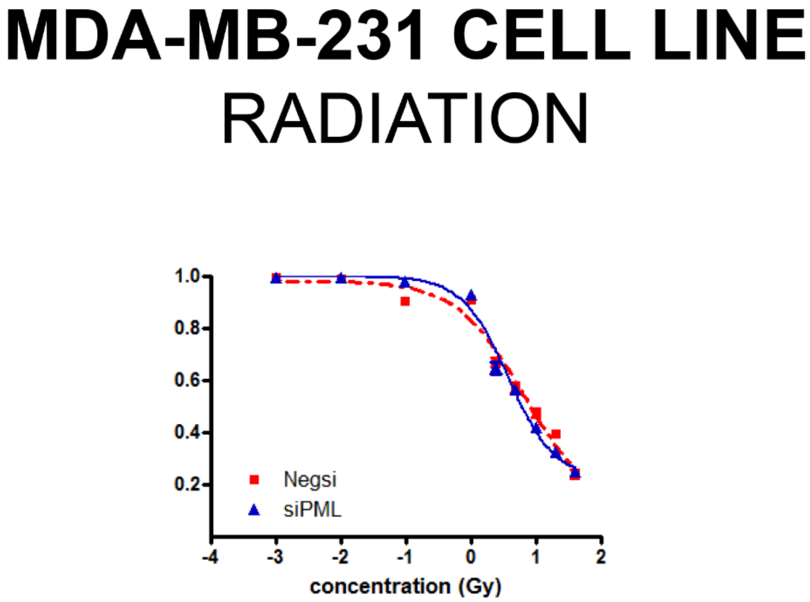

Supplemental Fig. S11: Cytotoxicity experiments performed in MDA-MB-231 cell lines treated with rapamycin. All 3 TFs were validated, as indicated by the red outline, and all were eQTL+M predictions.

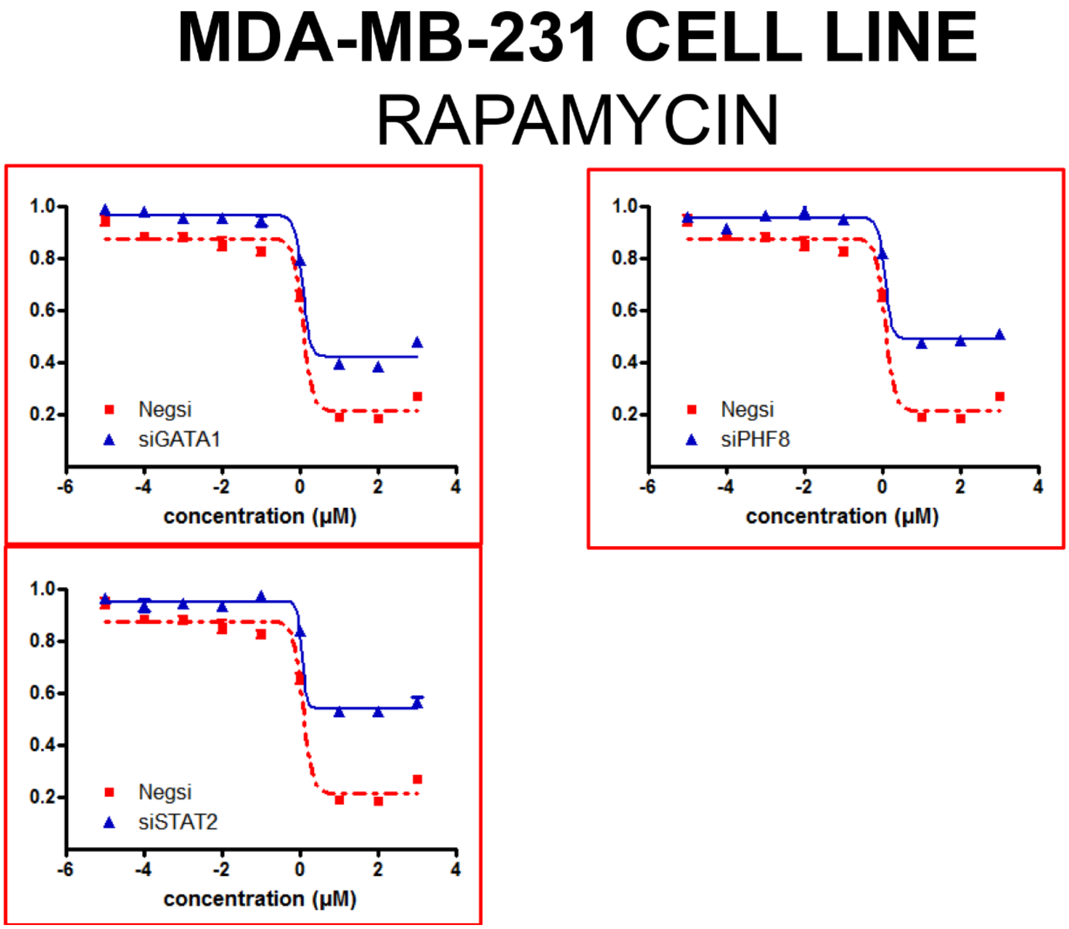

Supplemental Fig. S12: A Cytotoxicity experiment performed in MDA-MB-231 cell lines treated with docetaxel. The TF was validated, as indicated by the red outline, and was an eQTL+M prediction.

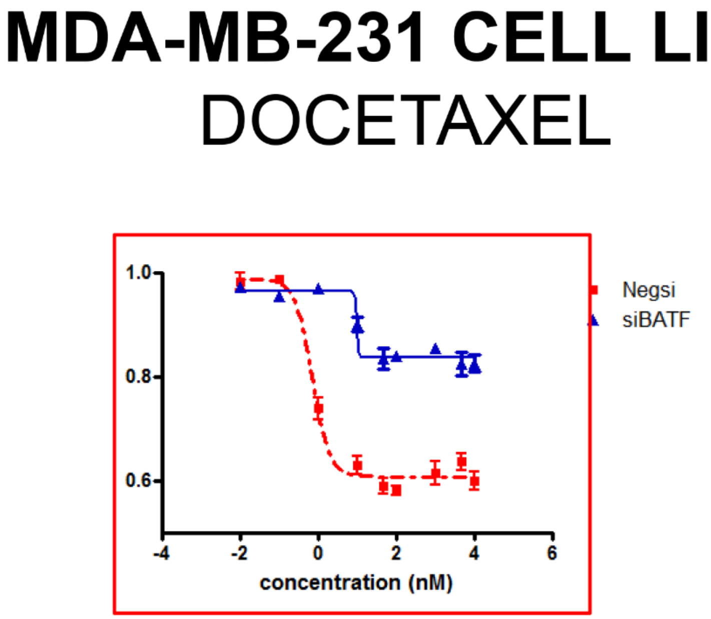

Supplemental Fig. S13: Cytotoxicity experiments performed in U251 cell lines treated with temozolomide (TMZ). Of the 4 TFs tested, only 1 was validated, as indicated by the red outline, and all were eQTL+M predictions, except *ZNF263* which was only supported by eQTM analysis.

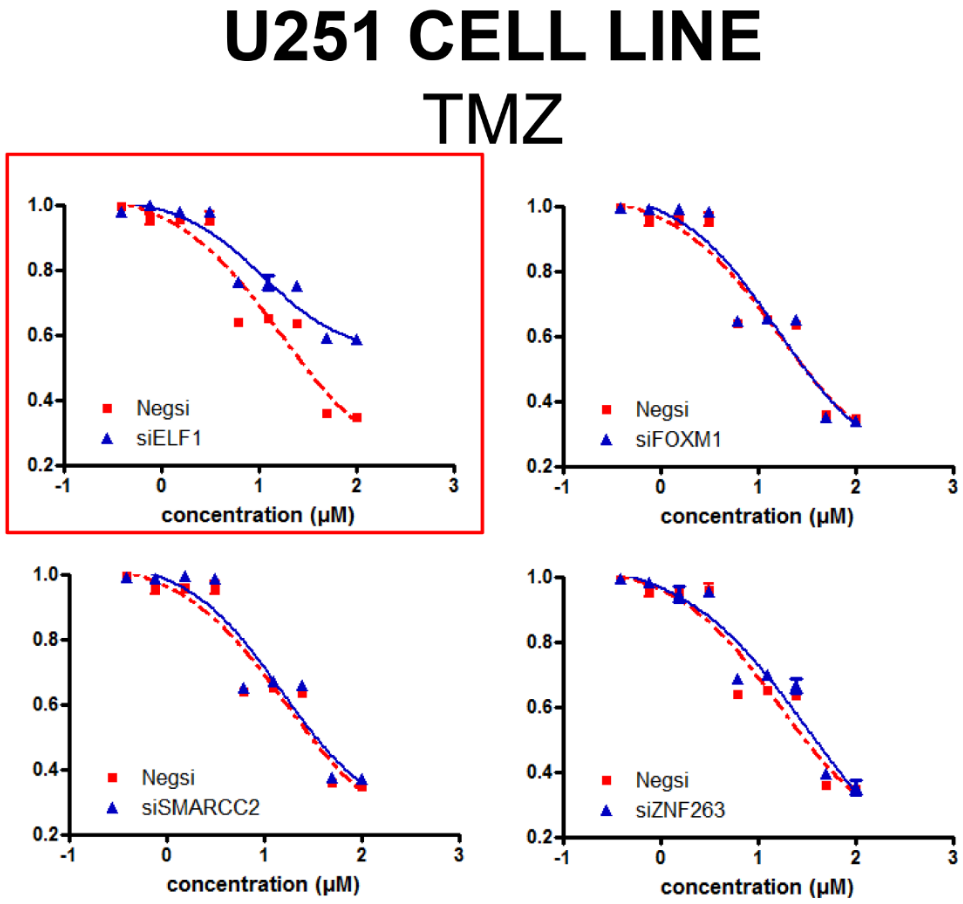

Supplemental Fig. S14: Distribution of GM12878 ChIP pGENMi eQTL-only Log-Likelihood Ratios (LLR) for tested (TF, drug) pairs for which the corresponding LLR with Composite ChIP data is < 1 (blue) or $>= 1$ (orange) respectively.

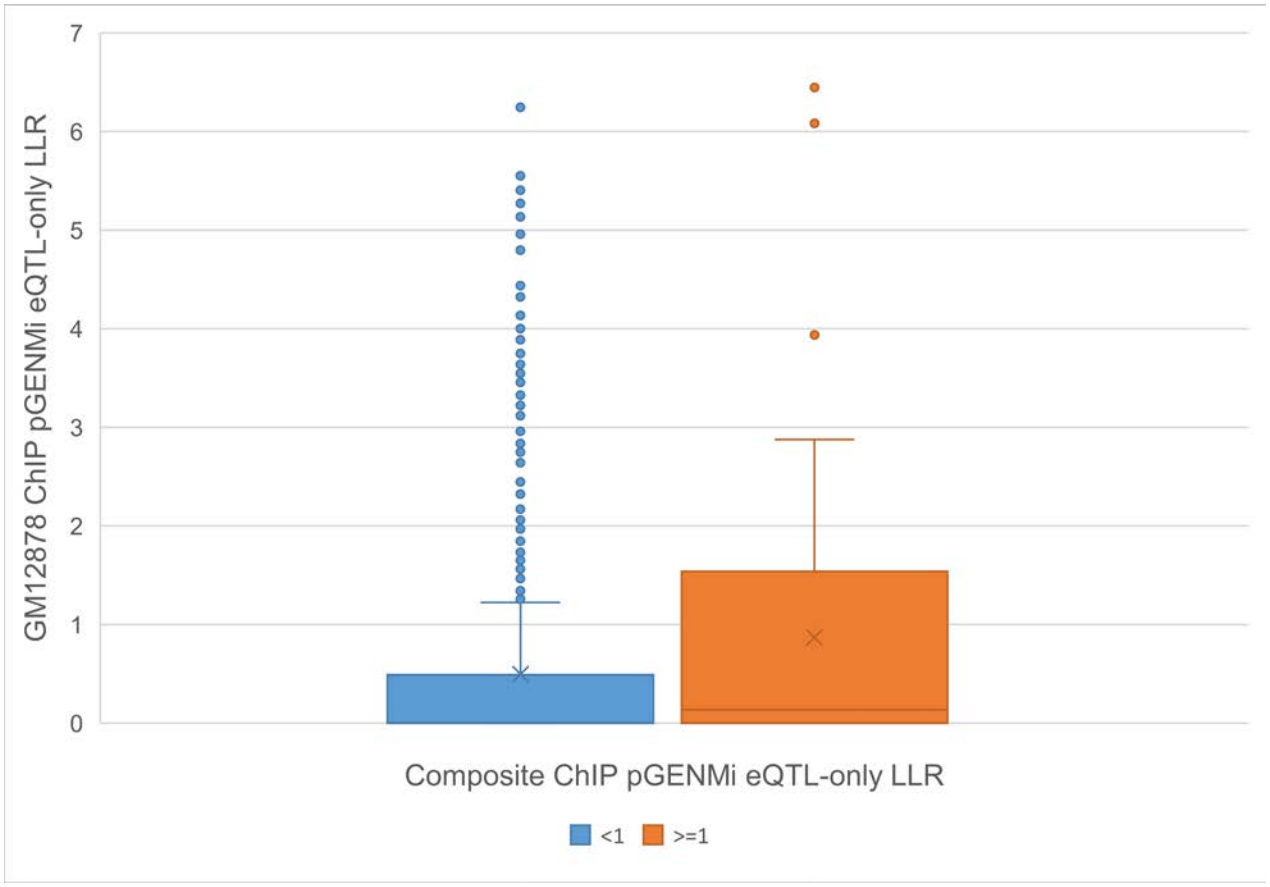

Supplemental Table S1A: Decomposition of the LLR ranks of eQTL and eQTM (TF, Drug) associations with no thresholds applied.

		**eQTM**	
		Top 500	Not Top
**eQTL**	Top 500	103	384
	Not Top	397	2595

Supplemental Table S1B: Decomposition of the LLR ranks of eQTL and eQTL+M (TF, Drug) associations with no thresholds applied.

		**eQTL+M**	
		Top 500	Not Top
**eQTL**	Top 500	309	161
	Not Top	173	2765

Supplemental Table S1C: Decomposition of the LLR ranks of eQTM and eQTL+M (TF, Drug) associations with no thresholds applied.

		**eQTL+M**	
		Top 500	Not Top
**eQTM**	Top 500	262	223
	Not Top	220	2703

Supplemental Table S2: Sub-population principal components extracted from EIGENSTRAT for each ethnicity separately, with Tracy-Widom statistics and p-values.

#	Ethnicity	Principal Comp	EigenValue	EigenDiff	TWStat	P-Val	Effect
1	CA	1	1.65	NA	60.84	2E-139	6767.46
2	CA	2	1.48	-1.72E-01	52.05	9.8E-111	9558.58
3	CA	3	1.25	-2.31E-01	18.28	1.58E-24	12486.00
4	CA	4	1.18	-7.04E-02	5.84	2.91E-06	13515.38
5	HCA	1	1.22	NA	9.94	6.74E-11	12980.28
6	AA	1	2.18	NA	73.83	9.9E-186	2955.53
7	AA	2	1.31	-8.68E-01	6.67	3.33E-07	5407.81

Supplemental Table S3: Concentration of administered drugs applied during experimental validation.

Drug	Concentrations										Scale
Paclitaxel	0	0.01	0.1	1	10	50	100	1000	5000	10000	µmol/L
Docetaxel	0	0.01	0.1	1	10	50	100	1000	5000	10000	µmol/L
Epirubicin	0	0.0156	0.03125	0.0625	0.125	0.25	0.55	1	2	4	µmol/L
Ara-C	0	0.00001	0.0001	0.001	0.01	0.1	1	10	100	1000	µmol/L
Gemcitabine	0	0.00001	0.0001	0.001	0.01	0.1	1	10	100	1000	µmol/L
6-MP	0	0.01	0.1	0.5	1	2.5	5	10	100	1000	µmol/L
6-TG	0	0.005	0.05	0.25	0.5	1	2.5	5	50	500	µmol/L
Carboplatin	0	0.0001	0.001	0.01	0.1	1	10	100	1000	10000	µmol/L
Cisplatin	0	1.25	2.5	5	10	20	40	60	80	100	µmol/L
Oxaliplatin	0	0.3125	0.625	1.25	2.5	5	10	20	40	80	µmol/L
Cladribine	0	0.0001	0.001	0.01	0.1	1	10	100	1000	10000	nmol/L
Rapamycin	0	0.00001	0.0001	0.001	0.01	0.1	1	10	100	1000	µmol/L
Temozolomide	0	0.39	0.78	1.56	3.125	6.25	12.5	25	50	100	µmol/L

Supplemental Table S4: The decomposition of TFs associated with Drugs among the top 90 (TF, Drug) pairs for the GM12878 eQTL-only pGENMi analysis.

**Drug**	**# TFs associated in top 90 (TF, Drug) pairs**
Rapamycin	1
Triciribine	1
Hypoxia	2
NAPQI	2
Gemcitabine	2
Fludarabine	4
Radiation	5
Arsenic	6
6-MP	14
Doxorubicin	16
Epirubicin	18
6-TG	19

Supplemental Table S5: The decomposition of Drugs associated with TFs among the top 90 (TF, Drug) pairs for the GM12878 eQTL-only pGENMi analysis.

Drug	# TFs associated in top 90 (TF, Drug) pairs
BHLHE40	1
EGR1	1
BATF	1
RELA	1
USF2	1
SPI1	1
BCL11A	1
USF1	1
NFYB	2
EBF1	2
PAX5	2
ZEB1	2
POU2F2	2
NFATC1	2
IRF4	2
MEF2A	2
BCL3	2
ATF2	2
TCF3	2
RUNX3	3
TCF12	3
SRF	3
MTA3	3
ZNF143	4
MAX	4
NFIC	4
MXI1	5
FOXM1	5
YY1	5
ELF1	5
SP1	5
SIN3A	5
PML	6

Supplemental Table S6: The number of TWAS genes (of 16183) at varying p-value thresholds and the number of pGENMi eQTL+M associations for those drugs at LLR >=3.

Drug	# of TWAS gene with p-value <=		# of pGENMi eQTL+M associations at LLR >=3
	0.005	0.0005	
6-MP	956	397	15
NAPQI	103	12	13
Temozolomide	324	133	10
Cisplatin	862	265	7
Oxaliplatin	562	227	7
Cytarabine	854	376	6
Gemcitabine	298	68	5
6-TG	882	377	4
Epirubicin	313	64	4
Radiation	181	27	4
Doxorubicin	318	74	3
Paclitaxel	176	34	3
Rapamycin	113	21	3
Triciribine	257	77	2
Arsenic	147	22	1
Carboplatin	1339	489	1
Docetaxel	138	27	1
Hypoxia	82	6	1
Cladribine	61	6	0
Everolimus	79	17	0
Fludarabine	88	12	0
Metformin	368	57	0
MPA	112	9	0
Methotrexate	56	7	0

Supplemental Table S7: The median and mean number of TWAS genes (of 16183) at varying p-value thresholds and the median and mean number of pGENMi eQTL+M associations at LLR >=3 for the Top 5 and Bottom 19 drugs by the # of pGENMi eQTL+M associations at LLR >= 3.

**Statistic**		**# of TWAS gene with p-value <=**		**# of pGENMi eQTL+M associations at LLR >=3**
		**0.005**	**0.0005**	
Top 5 Drugs	Median	562	227	10
	Mean	561.4	206.8	10.4
Bottom 19 Drugs	Median	176	27	1
	Mean	308.5	93.2	2

References

Aguirre MV, Todaro JS, Juaristi JA, Brandan NC. 2010. Murine erythropoietic impairment induced by paclitaxel: interactions of GATA-1 and erythroid Kruppel-like transcription factors, apoptotic related proteins and erythropoietin receptor. *Eur J Pharmacol* **636**: 42-51.

Alian OM, Azmi AS, Mohammad RM. 2012. Network insights on oxaliplatin anti-cancer mechanisms. *Clin Transl Med* **1**: 26.

Andrieux LO, Fautrel A, Bessard A, Guillouzo A, Baffet G, Langouet S. 2007. GATA-1 is essential in EGF-mediated induction of nucleotide excision repair activity and ERCC1 expression through ERK2 in human hepatoma cells. *Cancer research* **67**: 2114-2123.

Baldi A, Piccolo MT, Boccellino MR, Donizetti A, Cardillo I, La Porta R, Quagliuolo L, Spugnini EP, Cordero F, Citro G et al. 2011. Apoptosis induced by piroxicam plus cisplatin combined treatment is triggered by p21 in mesothelioma. *PLoS One* **6**: e23569.

Bavelloni A, Faenza I, Aluigi M, Ferri A, Toker A, Maraldi NM, Marmiroli S. 2000. Inhibition of phosphoinositide 3-kinase impairs pre-commitment cell cycle traverse and prevents differentiation in erythroleukaemia cells. *Cell Death Differ* **7**: 112-117.

Bernard M, Delabesse E, Novault S, Hermine O, Macintyre EA. 1998. Antiapoptotic effect of ectopic TAL1/SCL expression in a human leukemic T-cell line. *Cancer research* **58**: 2680-2687.

Beyer RP, Fry RC, Lasarev MR, McConnachie LA, Meira LB, Palmer VS, Powell CL, Ross PK, Bammler TK, Bradford BU et al. 2007. Multicenter study of acetaminophen hepatotoxicity reveals the importance of biological endpoints in genomic analyses. *Toxicological sciences : an official journal of the Society of Toxicology* **99**: 326-337.

Biswal BK, Beyrouthy MJ, Hever-Jardine MP, Armstrong D, Tomlinson CR, Christensen BC, Marsit CJ, Spinella MJ. 2012. Acute hypersensitivity of pluripotent testicular cancer-derived embryonal carcinoma to low-dose 5-aza deoxycytidine is associated with global DNA Damage-associated p53 activation, anti-pluripotency and DNA demethylation. *PLoS One* **7**: e53003.

Browning BL, Yu Z. 2009. Simultaneous genotype calling and haplotype phasing improves genotype accuracy and reduces false-positive associations for genome-wide association studies. *American journal of human genetics* **85**: 847-861.

Campbell KJ, Witty JM, Rocha S, Perkins ND. 2006. Cisplatin mimics ARF tumor suppressor regulation of RelA (p65) nuclear factor-kappaB transactivation. *Cancer research* **66**: 929-935.

Capaccione KM, Pine SR. 2013. The Notch signaling pathway as a mediator of tumor survival. *Carcinogenesis* **34**: 1420-1430.

Caporali S, Levati L, Graziani G, Muzi A, Atzori MG, Bonmassar E, Palmieri G, Ascierto PA, D'Atri S. 2012. NF-kappaB is activated in response to temozolomide in an AKT-dependent manner and confers protection against the growth suppressive effect of the drug. *J Transl Med* **10**: 252.

Chalastanis A, Penard-Lacronique V, Svrcek M, Defaweux V, Antoine N, Buhard O, Dumont S, Fabiani B, Renault I, Tubacher E et al. 2010. Azathioprine-induced carcinogenesis in mice according to Msh2 genotype. *J Natl Cancer Inst* **102**: 1731-1740.

Chan JY, Li L, Fan YH, Mu ZM, Zhang WW, Chang KS. 1997. Cell-cycle regulation of DNA damage-induced expression of the suppressor gene PML. *Biochemical and biophysical research communications* **240**: 640-646.

Chang CC, Chow CC, Tellier LC, Vattikuti S, Purcell SM, Lee JJ. 2015. Second-generation PLINK: rising to the challenge of larger and richer datasets. *Gigascience* **4**: 7.

Chen B, Liu J, Chang Q, Beezhold K, Lu Y, Chen F. 2013a. JNK and STAT3 signaling pathways converge on Akt-mediated phosphorylation of EZH2 in bronchial epithelial cells induced by arsenic. *Cell Cycle* **12**: 112-121.

Chen B, Xue Z, Yang G, Shi B, Yang B, Yan Y, Wang X, Han D, Huang Y, Dong W. 2013b. Akt-signal integration is involved in the differentiation of embryonal carcinoma cells. *PLoS One* **8**: e64877.

Chen HH, Kuo MT. 2013. Overcoming platinum drug resistance with copper-lowering agents. *Anticancer Res* **33**: 4157-4161.

Chiu SJ, Lee YJ, Hsu TS, Chen WS. 2009. Oxaliplatin-induced gamma-H2AX activation via both p53-dependent and -independent pathways but is not associated with cell cycle arrest in human colorectal cancer cells. *Chem Biol Interact* **182**: 173-182.

Chun JY, Hu Y, Pinder E, Wu J, Li F, Gao AC. 2007. Selenium inhibition of survivin expression by preventing Sp1 binding to its promoter. *Mol Cancer Ther* **6**: 2572-2580.

Chung D, Yang C, Li C, Gelernter J, Zhao H. 2014. GPA: A Statistical Approach to Prioritizing GWAS Results by Integrating Pleiotropy and Annotation. *PLoS genetics* **10**: e1004787.

Ciccia A, Nimonkar AV, Hu Y, Hajdu I, Achar YJ, Izhar L, Petit SA, Adamson B, Yoon JC, Kowalczykowski SC et al. 2012. Polyubiquitinated PCNA recruits the ZRANB3 translocase to maintain genomic integrity after replication stress. *Mol Cell* **47**: 396-409.

Cross-Knorr S, Lu S, Perez K, Guevara S, Brilliant K, Pisano C, Quesenberry PJ, Resnick MB, Chatterjee D. 2013. RKIP phosphorylation and STAT3 activation is inhibited by oxaliplatin and camptothecin and are associated with poor prognosis in stage II colon cancer patients. *BMC cancer* **13**: 463.

Dadarkar SS, Fonseca LC, Thakkar AD, Mishra PB, Rangasamy AK, Padigaru M. 2010. Effect of nephrotoxicants and hepatotoxicants on gene expression profile in human peripheral blood mononuclear cells. *Biochemical and biophysical research communications* **401**: 245-250.

Davis M, Li J, Knight E, Eldridge SR, Daniels KK, Bushel PR. 2015. Toxicogenomics profiling of bone marrow from rats treated with topotecan in combination with oxaliplatin: a mechanistic strategy to inform combination toxicity. *Frontiers in genetics* **6**: 14.

de Koning BA, Lindenbergh-Kortleve DJ, Pieters R, Buller HA, Renes IB, Einerhand AW. 2007. Alterations in epithelial and mesenchymal intestinal gene expression during doxorubicin-induced mucositis in mice. *Dig Dis Sci* **52**: 1814-1825.

Drago-Ferrante R, Santulli A, Di Fiore R, Giuliano M, Calvaruso G, Tesoriere G, Vento R. 2008. Low doses of paclitaxel potently induce apoptosis in human retinoblastoma Y79 cells by up-regulating E2F1. *Int J Oncol* **33**: 677-687.

Du Y, Shi L, Wang T, Liu Z, Wang Z. 2012. Nanog siRNA plus Cisplatin may enhance the sensitivity of chemotherapy in esophageal cancer. *J Cancer Res Clin Oncol* **138**: 1759-1767.

Elferink MG, Olinga P, van Leeuwen EM, Bauerschmidt S, Polman J, Schoonen WG, Heisterkamp SH, Groothuis GM. 2011. Gene expression analysis of precision-cut human liver slices indicates stable expression of ADME-Tox related genes. *Toxicol Appl Pharmacol* **253**: 57-69.

Fiedler J, Breckwoldt K, Remmele CW, Hartmann D, Dittrich M, Pfanne A, Just A, Xiao K, Kunz M, Muller T et al. 2015. Development of Long Noncoding RNA-Based Strategies to Modulate Tissue Vascularization. *J Am Coll Cardiol* **66**: 2005-2015.

Fielhaber JA, Han YS, Tan J, Xing S, Biggs CM, Joung KB, Kristof AS. 2009. Inactivation of mammalian target of rapamycin increases STAT1 nuclear content and transcriptional activity in alpha4- and protein phosphatase 2A-dependent fashion. *J Biol Chem* **284**: 24341-24353.

Ge Y, Jensen TL, Matherly LH, Taub JW. 2003. Physical and functional interactions between USF and Sp1 proteins regulate human deoxycytidine kinase promoter activity. *J Biol Chem* **278**: 49901-49910.

Ge Y, Jensen TL, Tatman DA, Stout ML, Buck SA, Ravindranath Y, Matherly LH, Taub JW. 2005. Role of USF1 in the differential expression of the human deoxycytidine kinase gene in acute myeloid leukemia. *Leukemia* **19**: 677-679.

Guilhamon P, Eskandarpour M, Halai D, Wilson GA, Feber A, Teschendorff AE, Gomez V, Hergovich A, Tirabosco R, Fernanda Amary M et al. 2013. Meta-analysis of IDH-mutant cancers identifies EBF1 as an interaction partner for TET2. *Nat Commun* **4**: 2166.

Guo C, Ding J, Yao L, Sun L, Lin T, Song Y, Sun L, Fan D. 2005. Tumor suppressor gene Runx3 sensitizes gastric cancer cells to chemotherapeutic drugs by downregulating Bcl-2, MDR-1 and MRP-1. *Int J Cancer* **116**: 155-160.

Gyorffy B, Surowiak P, Kiesslich O, Denkert C, Schafer R, Dietel M, Lage H. 2006. Gene expression profiling of 30 cancer cell lines predicts resistance towards 11 anticancer drugs at clinically achieved concentrations. *Int J Cancer* **118**: 1699-1712.

Hall AB, Newsome D, Wang Y, Boucher DM, Eustace B, Gu Y, Hare B, Johnson MA, Milton S, Murphy CE et al. 2014. Potentiation of tumor responses to DNA damaging therapy by the selective ATR inhibitor VX-970. *Oncotarget* **5**: 5674-5685.

Han Y, Zhang Y, Yang LH, Mi XY, Dai SD, Li QC, Xu HT, Yu JH, Li G, Zhao J et al. 2012. X-radiation inhibits histone deacetylase 1 and 2, upregulates Axin expression and induces apoptosis in non-small cell lung cancer. *Radiat Oncol* **7**: 183.

Hanson C, Cairns J, Wang L, Sinha S. 2015. Computational discovery of transcription factors associated with drug response. *The pharmacogenomics journal* doi:10.1038/tpj.2015.74.

He Y, Sun S, Sha H, Liu Z, Yang L, Xue Z, Chen H, Qi L. 2010. Emerging roles for XBP1, a sUPeR transcription factor. *Gene Expr* **15**: 13-25.

Hendriks G, Atallah M, Morolli B, Calleja F, Ras-Verloop N, Huijskens I, Raamsman M, van de Water B, Vrieling H. 2012. The ToxTracker assay: novel GFP reporter systems that provide mechanistic insight into the genotoxic properties of chemicals. *Toxicological sciences : an official journal of the Society of Toxicology* **125**: 285-298.

Hernandez-Vargas H, Rodriguez-Pinilla SM, Julian-Tendero M, Sanchez-Rovira P, Cuevas C, Anton A, Rios MJ, Palacios J, Moreno-Bueno G. 2007. Gene expression profiling of breast cancer cells in response to gemcitabine: NF-kappaB pathway activation as a potential mechanism of resistance. *Breast Cancer Res Treat* **102**: 157-172.

Houillier C, Wang X, Kaloshi G, Mokhtari K, Guillevin R, Laffaire J, Paris S, Boisselier B, Idbaih A, Laigle-Donadey F et al. 2010. IDH1 or IDH2 mutations predict longer survival and response to temozolomide in low-grade gliomas. *Neurology* **75**: 1560-1566.

Hour TC, Lai YL, Kuan CI, Chou CK, Wang JM, Tu HY, Hu HT, Lin CS, Wu WJ, Pu YS et al. 2010. Transcriptional up-regulation of SOD1 by CEBPD: a potential target for cisplatin resistant human urothelial carcinoma cells. *Biochem Pharmacol* **80**: 325-334.

Hu M, Fu X, Cui Y, Xu S, Xu Y, Dong Q, Sun L. 2015. Expression of KAP1 in epithelial ovarian cancer and its correlation with drug-resistance. *Int J Clin Exp Med* **8**: 17308-17320.

Jagtap S, Meganathan K, Gaspar J, Wagh V, Winkler J, Hescheler J, Sachinidis A. 2011. Cytosine arabinoside induces ectoderm and inhibits mesoderm expression in human embryonic stem cells during multilineage differentiation. *Br J Pharmacol* **162**: 1743-1756.

James MI, Iwuji C, Irving G, Karmokar A, Higgins JA, Griffin-Teal N, Thomas A, Greaves P, Cai H, Patel SR et al. 2015. Curcumin inhibits cancer stem cell phenotypes in ex vivo models of colorectal liver metastases, and is clinically safe and tolerable in combination with FOLFOX chemotherapy. *Cancer Lett* **364**: 135-141.

Jeter CR, Liu B, Liu X, Chen X, Liu C, Calhoun-Davis T, Repass J, Zaehres H, Shen JJ, Tang DG. 2011. NANOG promotes cancer stem cell characteristics and prostate cancer resistance to androgen deprivation. *Oncogene* **30**: 3833-3845.

Ju SY, Huang CY, Huang WC, Su Y. 2015. Identification of thiostrepton as a novel therapeutic agent that targets human colon cancer stem cells. *Cell death & disease* **6**: e1801.

Karnitz LM, Flatten KS, Wagner JM, Loegering D, Hackbarth JS, Arlander SJH, Vroman BT, Thomas MB, Baek Y-U, Hopkins KM et al. 2005. Gemcitabine-Induced Activation of Checkpoint Signaling Pathways That Affect Tumor Cell Survival. *Molecular Pharmacology* **68**: 1636-1644.

Kasof GM, Goyal L, White E. 1999. Btf, a novel death-promoting transcriptional repressor that interacts with Bcl-2-related proteins. *Mol Cell Biol* **19**: 4390-4404.

Kawamura N, Nimura K, Nagano H, Yamaguchi S, Nonomura N, Kaneda Y. 2015. CRISPR/Cas9-mediated gene knockout of NANOG and NANOGP8 decreases the malignant potential of prostate cancer cells. *Oncotarget* **6**: 22361-22374.

Kawano Y, Kikukawa Y, Fujiwara S, Wada N, Okuno Y, Mitsuya H, Hata H. 2013. Hypoxia reduces CD138 expression and induces an immature and stem cell-like transcriptional program in myeloma cells. *Int J Oncol* **43**: 1809-1816.

Kim JS, Kim BS, Kim J, Park CS, Chung IY. 2010. The phosphoinositide-3-kinase/Akt pathway mediates the transient increase in Nanog expression during differentiation of F9 cells. *Arch Pharm Res* **33**: 1117-1125.

Kim SH, Joshi K, Ezhilarasan R, Myers TR, Siu J, Gu C, Nakano-Okuno M, Taylor D, Minata M, Sulman EP et al. 2015. EZH2 protects glioma stem cells from radiation-induced cell death in a MELK/FOXM1-dependent manner. *Stem Cell Reports* **4**: 226-238.

Kobarg CB, Kobarg J, Crosara-Alberto DP, Theizen TH, Franchini KG. 2005. MEF2C DNA-binding activity is inhibited through its interaction with the regulatory protein Ki-1/57. *FEBS Lett* **579**: 2615-2622.

Kothandapani A, Gopalakrishnan K, Kahali B, Reisman D, Patrick SM. 2012. Downregulation of SWI/SNF chromatin remodeling factor subunits modulates cisplatin cytotoxicity. *Exp Cell Res* **318**: 1973-1986.

Kreisler A, Strissel PL, Strick R, Neumann SB, Schumacher U, Becker CM. 2010. Regulation of the NRSF/REST gene by methylation and CREB affects the cellular phenotype of small-cell lung cancer. *Oncogene* **29**: 5828-5838.

Lewis KA, Lilly KK, Reynolds EA, Sullivan WP, Kaufmann SH, Cliby WA. 2009. Ataxia telangiectasia and rad3-related kinase contributes to cell cycle arrest and survival after cisplatin but not oxaliplatin. *Mol Cancer Ther* **8**: 855-863.

Limonciel A, Moenks K, Stanzel S, Truisi GL, Parmentier C, Aschauer L, Wilmes A, Richert L, Hewitt P, Mueller SO et al. 2015. Transcriptomics hit the target: Monitoring of ligand-activated and stress response pathways for chemical testing. *Toxicol In Vitro* **30**: 7-18.

Lin HY, Hung SK, Lee MS, Chiou WY, Huang TT, Tseng CE, Shih LY, Lin RI, Lin JM, Lai YH et al. 2015. DNA methylome analysis identifies epigenetic silencing of FHIT as a determining factor for radiosensitivity in oral cancer: an outcome-predicting and treatment-implicating study. *Oncotarget* **6**: 915-934.

Liu X, Wang X, Bi Y, Bu P, Zhang M. 2015. The histone demethylase PHF8 represses cardiac hypertrophy upon pressure overload. *Exp Cell Res* **335**: 123-134.

Liu ZJ, Italiano J, Jr., Ferrer-Marin F, Gutti R, Bailey M, Poterjoy B, Rimsza L, Sola-Visner M. 2011. Developmental differences in megakaryocytopoiesis are associated with up-regulated TPO signaling through mTOR and elevated GATA-1 levels in neonatal megakaryocytes. *Blood* **117**: 4106-4117.

Low WK, Kong SW, Tan MG. 2010. Ototoxicity from combined Cisplatin and radiation treatment: an in vitro study. *Int J Otolaryngol* **2010**: 523976.

Lu W, Chen Z, Zhang H, Wang Y, Luo Y, Huang P. 2012. ZNF143 transcription factor mediates cell survival through upregulation of the GPX1 activity in the mitochondrial respiratory dysfunction. *Cell death & disease* **3**: e422.

Lu YS, Yeh PY, Chuang SE, Gao M, Kuo ML, Cheng AL. 2006. Glucocorticoids enhance cytotoxicity of cisplatin via suppression of NF-{kappa}B activation in the glucocorticoid receptor-rich human cervical carcinoma cell line SiHa. *J Endocrinol* **188**: 311-319.

Lundholm L, Haag P, Zong D, Juntti T, Mork B, Lewensohn R, Viktorsson K. 2013. Resistance to DNA-damaging treatment in non-small cell lung cancer tumor-initiating cells involves reduced DNA-PK/ATM activation and diminished cell cycle arrest. *Cell death & disease* **4**: e478.

Ma L, Nie L, Liu J, Zhang B, Song S, Sun M, Yang J, Yang Y, Fang X, Hu S et al. 2012. An RNA-seq-based gene expression profiling of radiation-induced tumorigenic mammary epithelial cells. *Genomics Proteomics Bioinformatics* **10**: 326-335.

Masliah-Planchon J, Bieche I, Guinebretiere JM, Bourdeaut F, Delattre O. 2015. SWI/SNF chromatin remodeling and human malignancies. *Annu Rev Pathol* **10**: 145-171.

mod Encode Consortium, Roy S, Ernst J, Kharchenko PV, Kheradpour P, Negre N, Eaton ML, Landolin JM, Bristow CA, Ma L et al. 2010. Identification of functional elements and regulatory circuits by Drosophila modENCODE. *Science* **330**: 1787-1797.

Moffit JS, Koza-Taylor PH, Holland RD, Thibodeau MS, Beger RD, Lawton MP, Manautou JE. 2007. Differential gene expression in mouse liver associated with the hepatoprotective effect of clofibrate. *Toxicol Appl Pharmacol* **222**: 169-179.

Msaki A, Sanchez AM, Koh LF, Barre B, Rocha S, Perkins ND, Johnson RF. 2011. The role of RelA (p65) threonine 505 phosphorylation in the regulation of cell growth, survival, and migration. *Mol Biol Cell* **22**: 3032-3040.

New M, Olzscha H, La Thangue NB. 2012. HDAC inhibitor-based therapies: can we interpret the code? *Mol Oncol* **6**: 637-656.

Nishimoto A, Kugimiya N, Hosoyama T, Enoki T, Li TS, Hamano K. 2014. HIF-1alpha activation under glucose deprivation plays a central role in the acquisition of anti-apoptosis in human colon cancer cells. *Int J Oncol* **44**: 2077-2084.

Ordelheide AM, Gerst F, Rothfuss O, Heni M, Haas C, Thielker I, Herzberg-Schafer S, Bohm A, Machicao F, Ullrich S et al. 2013. Nor-1, a novel incretin-responsive regulator of insulin genes and insulin secretion. *Mol Metab* **2**: 243-255.

Ordentlich P, Yan Y, Zhou S, Heyman RA. 2003. Identification of the antineoplastic agent 6-mercaptopurine as an activator of the orphan nuclear hormone receptor Nurr1. *J Biol Chem* **278**: 24791-24799.

Pines A, Kelstrup CD, Vrouwe MG, Puigvert JC, Typas D, Misovic B, de Groot A, von Stechow L, van de Water B, Danen EH et al. 2011. Global phosphoproteome profiling reveals unanticipated networks responsive to cisplatin treatment of embryonic stem cells. *Mol Cell Biol* **31**: 4964-4977.

Price AL, Patterson NJ, Plenge RM, Weinblatt ME, Shadick NA, Reich D. 2006. Principal components analysis corrects for stratification in genome-wide association studies. *Nat Genet* **38**: 904-909.

Prot JM, Bunescu A, Elena-Herrmann B, Aninat C, Snouber LC, Griscom L, Razan F, Bois FY, Legallais C, Brochot C et al. 2012. Predictive toxicology using systemic biology and liver microfluidic "on chip" approaches: application to acetaminophen injury. *Toxicol Appl Pharmacol* **259**: 270-280.

Rabik CA, Dolan ME. 2007. Molecular mechanisms of resistance and toxicity associated with platinating agents. *Cancer Treat Rev* **33**: 9-23.

Ray S, Lu Y, Kaufmann SH, Gustafson WC, Karp JE, Boldogh I, Fields AP, Brasier AR. 2004. Genomic mechanisms of p210BCR-ABL signaling: induction of heat shock protein 70

through the GATA response element confers resistance to paclitaxel-induced apoptosis. *J Biol Chem* **279**: 35604-35615.

Romero-Ramirez L, Cao H, Nelson D, Hammond E, Lee AH, Yoshida H, Mori K, Glimcher LH, Denko NC, Giaccia AJ et al. 2004. XBP1 is essential for survival under hypoxic conditions and is required for tumor growth. *Cancer research* **64**: 5943-5947.

Russo AJ, Magro PG, Hu Z, Li WW, Peters R, Mandola J, Banerjee D, Bertino JR. 2006. E2F-1 overexpression in U2OS cells increases cyclin B1 levels and cdc2 kinase activity and sensitizes cells to antimitotic agents. *Cancer research* **66**: 7253-7260.

Saijo K, Winner B, Carson CT, Collier JG, Boyer L, Rosenfeld MG, Gage FH, Glass CK. 2009. A Nurr1/CoREST pathway in microglia and astrocytes protects dopaminergic neurons from inflammation-induced death. *Cell* **137**: 47-59.

Salomoni P, Bernardi R, Bergmann S, Changou A, Tuttle S, Pandolfi PP. 2005. The promyelocytic leukemia protein PML regulates c-Jun function in response to DNA damage. *Blood* **105**: 3686-3690.

Sanchez-Tillo E, Fanlo L, Siles L, Montes-Moreno S, Moros A, Chiva-Blanch G, Estruch R, Martinez A, Colomer D, Gyorffy B et al. 2014. The EMT activator ZEB1 promotes tumor growth and determines differential response to chemotherapy in mantle cell lymphoma. *Cell Death Differ* **21**: 247-257.

Shahzad MM, Mangala LS, Han HD, Lu C, Bottsford-Miller J, Nishimura M, Mora EM, Lee JW, Stone RL, Pecot CV et al. 2011. Targeted delivery of small interfering RNA using reconstituted high-density lipoprotein nanoparticles. *Neoplasia* **13**: 309-319.

Shao C, Folkard M, Held KD, Prise KM. 2008. Estrogen enhanced cell-cell signalling in breast cancer cells exposed to targeted irradiation. *BMC cancer* **8**: 184.

Sheng WJ, Jiang H, Wu DL, Zheng JH. 2013. Early responses of the STAT3 pathway to platinum drugs are associated with cisplatin resistance in epithelial ovarian cancer. *Braz J Med Biol Res* **46**: 650-658.

Stuart HT, van Oosten AL, Radzisheuskaya A, Martello G, Miller A, Dietmann S, Nichols J, Silva JC. 2014. NANOG amplifies STAT3 activation and they synergistically induce the naive pluripotent program. *Curr Biol* **24**: 340-346.

Tang H, Liu YJ, Liu M, Li X. 2007. Establishment and gene analysis of an oxaliplatin-resistant colon cancer cell line THC8307/L-OHP. *Anticancer Drugs* **18**: 633-639.

Tardito S, Isella C, Medico E, Marchio L, Bevilacqua E, Hatzoglou M, Bussolati O, Franchi-Gazzola R. 2009. The thioxotriazole copper(II) complex A0 induces endoplasmic reticulum stress and paraptotic death in human cancer cells. *J Biol Chem* **284**: 24306-24319.

Tel J, Hato SV, Torensma R, Buschow SI, Figdor CG, Lesterhuis WJ, de Vries IJ. 2012. The chemotherapeutic drug oxaliplatin differentially affects blood DC function dependent on environmental cues. *Cancer Immunol Immunother* **61**: 1101-1111.

The Encode Project Consortium. 2012. An integrated encyclopedia of DNA elements in the human genome. *Nature* **489**: 57-74.

Thota S, Viny AD, Makishima H, Spitzer B, Radivoyevitch T, Przychodzen B, Sekeres MA, Levine RL, Maciejewski JP. 2014. Genetic alterations of the cohesin complex genes in myeloid malignancies. *Blood* **124**: 1790-1798.

Tubbs AT, Dorsett Y, Chan E, Helmink B, Lee BS, Hung P, George R, Bredemeyer AL, Mittal A, Pappu RV et al. 2014. KAP-1 promotes resection of broken DNA ends not protected by gamma-H2AX and 53BP1 in G(1)-phase lymphocytes. *Mol Cell Biol* **34**: 2811-2821.

Valdez BC, Wang G, Murray D, Nieto Y, Li Y, Shah J, Turturro F, Wang M, Weber DM, Champlin RE et al. 2013. Mechanistic studies on the synergistic cytotoxicity of the nucleoside analogs gemcitabine and clofarabine in multiple myeloma: relevance of p53 and its clinical implications. *Exp Hematol* **41**: 719-730.

Vangipuram SD, Buck SA, Lyman WD. 2012. Wnt pathway activity confers chemoresistance to cancer stem-like cells in a neuroblastoma cell line. *Tumour Biol* **33**: 2173-2183.

Vellinga TT, Borovski T, de Boer VC, Fatrai S, van Schelven S, Trumpi K, Verheem A, Snoeren N, Emmink BL, Koster J et al. 2015. SIRT1/PGC1alpha-Dependent Increase in Oxidative Phosphorylation Supports Chemotherapy Resistance of Colon Cancer. *Clinical cancer research : an official journal of the American Association for Cancer Research* **21**: 2870-2879.

Watanabe R, Ui A, Kanno S, Ogiwara H, Nagase T, Kohno T, Yasui A. 2014. SWI/SNF factors required for cellular resistance to DNA damage include ARID1A and ARID1B and show interdependent protein stability. *Cancer research* **74**: 2465-2475.

Weisenberger DJ. 2014. Characterizing DNA methylation alterations from The Cancer Genome Atlas. *The Journal of clinical investigation* **124**: 17-23.

Wellner U, Schubert J, Burk UC, Schmalhofer O, Zhu F, Sonntag A, Waldvogel B, Vannier C, Darling D, zur Hausen A et al. 2009. The EMT-activator ZEB1 promotes tumorigenicity by repressing stemness-inhibiting microRNAs. *Nat Cell Biol* **11**: 1487-1495.

Xu N, Shen C, Luo Y, Xia L, Xue F, Xia Q, Zhang J. 2012. Upregulated miR-130a increases drug resistance by regulating RUNX3 and Wnt signaling in cisplatin-treated HCC cell. *Biochemical and biophysical research communications* **425**: 468-472.

Xu ZX, Timanova-Atanasova A, Zhao RX, Chang KS. 2003. PML colocalizes with and stabilizes the DNA damage response protein TopBP1. *Mol Cell Biol* **23**: 4247-4256.

Yamini B, Yu X, Dolan ME, Wu MH, Darga TE, Kufe DW, Weichselbaum RR. 2007. Inhibition of nuclear factor-kappaB activity by temozolomide involves O6-methylguanine induced inhibition of p65 DNA binding. *Cancer research* **67**: 6889-6898.

Yoshimura A, Seki M, Hayashi T, Kusa Y, Tada S, Ishii Y, Enomoto T. 2006. Functional relationships between Rad18 and WRNIP1 in vertebrate cells. *Biol Pharm Bull* **29**: 2192-2196.

Zaytseva YY, Valentino JD, Gulhati P, Evers BM. 2012. mTOR inhibitors in cancer therapy. *Cancer Lett* **319**: 1-7.

Zhang N, Wu X, Yang L, Xiao F, Zhang H, Zhou A, Huang Z, Huang S. 2012. FoxM1 inhibition sensitizes resistant glioblastoma cells to temozolomide by downregulating the expression of DNA-repair gene Rad51. *Clinical cancer research : an official journal of the American Association for Cancer Research* **18**: 5961-5971.

Zhou Y, Wan G, Spizzo R, Ivan C, Mathur R, Hu X, Ye X, Lu J, Fan F, Xia L et al. 2014. miR-203 induces oxaliplatin resistance in colorectal cancer cells by negatively regulating ATM kinase. *Mol Oncol* **8**: 83-92.
